# Transcranial Magnetic Stimulation and Transcranial Direct Current Stimulation Across Mental Disorders: A Systematic Review and Dose-Response Meta-Analysis

**DOI:** 10.1001/jamanetworkopen.2024.12616

**Published:** 2024-05-22

**Authors:** Michel Sabé, Joshua Hyde, Catharina Cramer, Antonia-Leonie Eberhard, Alessio Crippa, André Russowsky Brunoni, André Aleman, Stefan Kaiser, David S. Baldwin, Matthew Garner, Othman Sentissi, Jess G. Fiedorowicz, Valerie Brandt, Samuele Cortese, Marco Solmi

**Affiliations:** 1 Division of Adult Psychiatry, Department of Psychiatry, University Hospitals of Geneva, Thonex, Switzerland; 2 Faculty of Medicine, University of Geneva, Geneva, Switzerland; 3 Centre for Innovation in Mental Health, School of Psychology, University of Southampton, United Kingdom; 4 Clinic of Psychiatry, Social Psychiatry and Psychotherapy, Hannover Medical School, Hanover, Germany; 5 Department of Medical Epidemiology and Biostatistics, Karolinska Institutet, Stockholm, Sweden; 6 Departamento e Instituto de Psiquiatria da Faculdade de Medicina da Universidade de São Paulo, Universidade de São Paulo, Brazil; 7 Department of Biomedical Sciences of Cells and Systems, Section Cognitive Neurosciences, University Medical Center Groningen, University of Groningen, the Netherlands; 8 Clinical and Experimental Sciences, Faculty of Medicine, University of Southampton, United Kingdom; 9 University Department of Psychiatry and Mental Health, University of Cape Town, South Africa; 10 Clinical and Experimental Sciences (CNS and Psychiatry), Faculty of Medicine, University of Southampton, United Kingdom; 11 The Ottawa Hospital and Ottawa Hospital Research Institute, Ontario, Canada; 12 Department of Psychiatry, University of Ottawa, Ontario, Canada; 13 Hassenfeld Children’s Hospital at New York University Langone, New York University Child Study Center, New York, New York; 14 Division of Psychiatry and Applied Psychology, School of Medicine, University of Nottingham, United Kingdom; 15 DiMePRe-J-Department of Precision and Regenerative Medicine-Jonic Area, University of Bari "Aldo Moro", Bari, Italy; 16 School of Epidemiology and Public Health, Faculty of Medicine, University of Ottawa, Ontario, Canada; 17 Department of Child and Adolescent Psychiatry, Charité Universitätsmedizin, Berlin, Germany; 18 Department of Mental Health, The Ottawa Hospital, Ontario, Canada; 19 SIENCES Laboratory, Department of Psychiatry, University of Ottawa, Ontario, Canada

## Abstract

**Importance:**

Noninvasive brain stimulation (NIBS) interventions have been shown to be efficacious in several mental disorders, but the optimal dose stimulation parameters for each disorder are unknown.

**Objective:**

To define NIBS dose stimulation parameters associated with the greatest efficacy in symptom improvement across mental disorders.

**Data Sources:**

Studies were drawn from an updated (to April 30, 2023) previous systematic review based on a search of PubMed, OVID, and Web of Knowledge.

**Study Selection:**

Randomized clinical trials were selected that tested transcranial magnetic stimulation (TMS) or transcranial direct current stimulation (tDCS) for any mental disorder in adults aged 18 years or older.

**Data Extraction and Synthesis:**

Two authors independently extracted the data. A 1-stage dose-response meta-analysis using a random-effects model was performed. Sensitivity analyses were conducted to test robustness of the findings. This study followed the Preferred Reporting Items for Systematic Reviews and Meta-analyses (PRISMA) reporting guideline.

**Main Outcomes and Measures:**

The main outcome was the near-maximal effective doses of total pulses received for TMS and total current dose in coulombs for tDCS.

**Results:**

A total of 110 studies with 4820 participants (2659 men [61.4%]; mean [SD] age, 42.3 [8.8] years) were included. The following significant dose-response associations emerged with bell-shaped curves: (1) in schizophrenia, high-frequency (HF) TMS on the left dorsolateral prefrontal cortex (LDLPFC) for negative symptoms (χ^2^ = 9.35; *df* = 2; *P* = .009) and TMS on the left temporoparietal junction for resistant hallucinations (χ^2^ = 36.52; *df* = 2; *P* < .001); (2) in depression, HF-DLPFC TMS (χ^2^ = 14.49; *df* = 2; *P* < .001); (3) in treatment-resistant depression, LDLPFC tDCS (χ^2^ = 14.56; *df* = 2; *P* < .001); and (4) in substance use disorder, LDLPFC tDCS (χ^2^ = 33.63; *df* = 2; *P* < .001). The following significant dose-response associations emerged with plateaued or ascending curves: (1) in depression, low-frequency (LF) TMS on the right DLPFC (RDLPFC) with ascending curve (χ^2^ = 25.67; *df* = 2; *P* = .001); (2) for treatment-resistant depression, LF TMS on the bilateral DLPFC with ascending curve (χ^2^ = 5.86; *df* = 2; *P* = .004); (3) in obsessive-compulsive disorder, LF-RDLPFC TMS with ascending curve (χ^2^ = 20.65; *df* = 2; *P* < .001) and LF TMS on the orbitofrontal cortex with a plateaued curve (χ^2^ = 15.19; *df* = 2; *P* < .001); and (4) in posttraumatic stress disorder, LF-RDLPFC TMS with ascending curve (χ^2^ = 54.15; *df* = 2; *P* < .001). Sensitivity analyses confirmed the main findings.

**Conclusions and Relevance:**

The study findings suggest that NIBS yields specific outcomes based on dose parameters across various mental disorders and brain regions. Clinicians should consider these dose parameters when prescribing NIBS. Additional research is needed to prospectively validate the findings in randomized, sham-controlled trials and explore how other parameters contribute to the observed dose-response association.

## Key Points

**Question:**

What is the association between dose of transcranial magnetic stimulation (TMS) and transcranial direct current stimulation (tDCS) interventions and response with core symptom severity across mental disorders?

**Findings:**

This systematic review and dose-response meta-analysis of 110 studies in 4820 participants found that significant dose-response associations were observed for schizophrenia, depression, obsessive-compulsive disorder, and substance use disorders, with distinct curve shapes. Most of these curves exhibited a bell-shaped pattern, indicating that TMS and tDCS may have distinct near-maximal effective doses for each disorder and stimulation site.

**Meaning:**

These findings offer guidance for clinicians and researchers, emphasizing the need for further refinement of dose-response models in TMS and tDCS to enhance comprehension of their outcomes for symptom reduction in specific mental disorders.

## Teaser

This systematic review and meta-analysis investigates which dose stimulation parameters for transcranial magnetic stimulation and transcranial direct current stimulation are associated with greatest symptom improvement across mental disorders.

## Introduction

Evidence providing support for the efficacy of noninvasive brain stimulation (NIBS) techniques, including transcranial magnetic stimulation (TMS) and transcranial direct current stimulation (tDCS), in the treatment of mental disorders, such as major depression, schizophrenia, and obsessive-compulsive disorder (OCD), has accumulated steadily. Currently, there are different TMS and tDCS protocols and devices that have been approved by the US Food and Drug Administration for clinical practice across several disorders.^1,2^

A previous umbrella review summarizing the body of research on NIBS in mental health mapped meta-analytic evidence in support of both TMS and tDCS across mental disorders.^3^ The results showed that high-frequency (HF) TMS to the left dorsolateral prefrontal cortex (DLPFC) and tDCS had the highest quality of evidence in terms of response, remission, and continuous antidepressant outcomes compared with sham.

However, despite the increased use of NIBS interventions in clinical practice, a standard dose definition currently is not available.^4,5^ For instance, the dose of a TMS protocol has been investigated as the number of pulses per session, number of sessions, and frequency, while for tDCS, parameters such as current intensity, session duration, and number of sessions have been used. Moreover, results from randomized clinical trials (RCTs) and meta-analyses have been inconsistent in terms of the best dose-related parameters for NIBS.^6,7,8^ Lack of consistent terminology poses a challenge for both clinical practice and research, as trials have used different combinations of parameters, resulting in increased heterogeneity and limitations in establishing a robust evidence base for specific treatment strategies.

Dose-response meta-analyses are increasingly undertaken in evidence synthesis of psychopharmacologic treatments^9,10^ and allow for the estimation of doses at which 50% and 95% of the maximum treatment efficacy may be achieved. Moreover, dose-response meta-analyses allow the identification of nonefficacious doses and a maximum dose above which efficacy may not be improved or even reduced. Importantly, dose-response meta-analyses also allow for the estimation of potential treatment effects achieved by doses that have not yet been explored in RCTs, guiding future clinical research and implementation. Three different types of curves are usually found: ascending/descending curves, which suggest that higher doses are associated with further improvement or worsening of symptoms; plateau curves, which suggest the reaching of a threshold after a specific dose; and bell-shaped curves, which suggest that improvements are found up to certain doses, with a reduction of benefits at higher doses.^9^

Identifying and understanding the dose-response association of specific NIBS parameters is therefore key to inform clinical practice. To fill this gap, we conducted a series of dose-response meta-analyses for TMS and tDCS across several mental disorders and symptoms to investigate the size and shape of associations between changes in specific parameters and treatment response. Our primary objective was to determine the near-maximal effective doses (defined as the dose beyond which additional benefit would be unlikely to occur) of total pulses received per number of sessions for TMS and total coulombs for tDCS. We also explored the shape of the dose-response association obtained for each stimulation site. Therefore, we included all mental disorders with a number that we deemed sufficient RCTs (>3) with at least 2 different doses of stimulation to produce dose-response models. The secondary objective was to explore whether other parameters may influence dose-response associations.

## Methods

This systematic review and dose-response meta-analysis is based on an update of a previous systematic review registered with the International Prospective Register of Systematic Reviews (CRD42021250057). Considering the nature of the study design, no ethical review was needed. This study follows the Preferred Reporting Items for Systematic Reviews and Meta-Analyses (PRISMA) 2020 reporting guideline.^11^

### Inclusion Criteria

The inclusion criteria were similar to those in our previous systematic review^12^ (eFigure 1 in Supplement 1), albeit with the addition of the following crucial inclusion criterion for the present analyses: Trials using TMS (including intermittent theta-burst stimulation [iTBS]) or tDCS were required to use at least 2 different doses of stimulation for the same disorder in addition to a sham stimulation for a similar stimulation site. The total amount of pulse and of coulombs were chosen to quantify the dose of stimulation received, considering that these parameters are also closely linked to the total duration of stimulation.

### Search Strategy and Selection of Studies

We updated to April 30, 2023, using the same search strategy, the database of a systematic review that included studies up to April 26, 2021,^12^ based on a search in PubMed, OVID, and Web of Knowledge with no restrictions. Search terms were a combination of keywords and Medical Subject Heading terms using (random*) and (TMS or tDCS) and a list of mental health disorders from the *Diagnostic and Statistical Manual for Mental Disorders, 5th Edition*, or *International Classification of Diseases 11th Edition*. Reference lists of retrieved articles were also screened for additional articles. Two authors (J.H. and M.Sa.) examined reports independently. All data were extracted in duplicate and independently by authors M.Sa. and J.H. Any disagreement was resolved by consensus. We report the full list of included studies and of the excluded studies after checking the full text, with reasons for exclusion, in eAppendix 2 in Supplement 1. Risk of bias was assessed with version 2 of the Cochrane risk-of-bias tool for articles that were added to the database following the update.

### Statistical Analysis

R, version 4.2.2 statistical software (R Foundation for Statistical Computing) was used for all analyses. The standardized mean difference (Cohen *d*) was used as the effect size measure, and a random-effects model was used to account for between-study variability. We used the doresmeta package developed by Crippa and Orsini^13^ to conduct a 1-stage dose-response meta-analysis using a restricted cubic spline model (nonlinear model) with 3 knots located at the 25th, 50th, and 75th percentiles of the overall dose distribution.

Separate analyses were conducted to investigate the association between total pulses delivered (TMS) or the total coulombs received (tDCS) with symptoms change. These analyses were performed for each stimulation site and frequency (low vs high). Dose-response curves extracted from the data were examined to estimate the 95% effective dose (ED_95_) and median effective dose. The ED_95_ represents the near-maximal effective dose of the maximum effect compared with sham stimulation.

In the presence of a significant dose-response association, sensitivity analyses were conducted to exclude studies with a high risk of bias. Heterogeneity was assessed using a multivariable extension of the *I*^2^ statistic, the variance partition coefficient defined as the ratio of the between-study component to the total residual.^13^

## Results

### Included Studies

We included 110 studies, encompassing a total of 4820 participants.^14,15,16,17,18,19,20,21,22,23,24,25,26,27,28,29,30,31,32,33,34,35,36,37,38,39,40,41,42,43,44,45,46,47,48,49,50,51,52,53,54,55,56,57,58,59,60,61,62,63,64,65,66,67,68,69,70,71,72,73,74,75,76,77,78,79,80,81,82,83,84,85,86,87,88,89,90,91,92,93,94,95,96,97,98,99,100,101,102,103,104,105,106,107,108,109,110,111,112,113,114,115,116,117,118,119,120,121,122,123,124^ The majority of participants were male (2959 [61.4%] compared with 1861 females [38.6%]), and the mean (SD) age was 43 (8.8) years.

The details of all retained studies and the overall results for dose equivalents are reported in eTable 1 in Supplement 1 and the Table, respectively. Overall, considering the important heterogeneity found for borderline disorder and autism spectrum disorder, we included 33 studies on schizophrenia,^24,26,31,33,34,38,39,45,51,52,53,58,59,62,65,69,71,75,76,81,89,95,96,97,98,105,115,117,118,119,120,122,123^ 43 on mood disorders,^18,20,21,23,25,27,28,30,32,35,42,43,44,47,48,49,56,57,61,63,67,70,73,74,82,83,84,87,88,90,91,94,101,102,103,107,110,111,112,113,114,121,124^ 18 on OCD,^17,19,22,41,50,54,60,64,68,77,78,79,86,92,93,99,100,104^ 4 on posttraumatic stress disorder (PTSD),^16,29,85,116^ and 11 on substance use disorder (SUD).^14,15,36,37,40,46,55,66,80,108,109^ We also included 32 studies on treatment-resistant depression.^18,20,21,23,25,27,28,30,32,35,43,44,47,56,57,63,83,84,87,88,90,91,94,107,111,112,114,121,124^ and 5 on persistent auditory hallucinations.^24,33,52,89,118^

**Table.** Dose Equivalents for TMS and tDCS With Consideration of Near-Maximal Total Pulses or Total Coulombs Received

Stimulation site	No. of studies	No. of patients	Mean (SD)				Mean total pulses or coulombs delivered among included studies (range)	Total pulses or coulombs corresponding to the ED_50_	Total pulses or coulombs corresponding to the ED_95_ after exclusion of high-risk studies (*P* value)^a^	Heterogeneity, %^b^	Figure
			Age, y	No. of sessions	Duration of trials, wk	Frequency among all studies					
**TMS for patients with schizophrenia**											
TMS negative symptoms											
HF-LDLPFC	14^53,59,71,95,96,97,98,105,115,117,119,120,122,123^	909	48.1	19.1	3.85	16.6 Hz	30 675 (8000-64 000)	9051	21 695 (<.001); 16 720 (.05)^a^	82; >75	Figure 1A, eFigures 13 and 19 in Supplement 1
BLDLPFC	2^26,38^	57	40.4	25	3.5	15 Hz	45 000 (30 000-60 000)	7549	18 207 (>.99)	>95	eFigure 2 in Supplement 1
TMS positive symptoms											
HF-LDLPFC	14^53,59,71,95,96,97,98,105,115,117,119,120,122,123^	909	48.1	19.1	3.85	16.6 Hz	30 675 (8000-64 000)	NA	NA	>75	Figure 1B
BLDLPFC	2^26,38^	57	40.4	25	3.5	15 Hz	45 000 (30 000-60 000)	7537	18 159 (.34)	>95	eFigure 3 in Supplement 1
TMS-resistant hallucinations											
LF-LTPJ	5^24,33,52,89,118^	159	33.1	11.4	1.7	1 Hz	14 360 (9000-28 800)	2102	5000 (<.001)	>95	eFigure 2C in Supplement 1
**tDCS for patients with schizophrenia**											
tDCS negative symptoms											
LDLPFC	5^39,51,58,62,76^	142	39.4	15	1.8	2 mA	38.4 C (12-96 C)	19 C	35 C (.22)	>95	eFigure 4A in Supplement 1
tDCS positive symptoms											
LDLPFC	5^39,51,58,62,76^	142	39.4	15	1.8	2 mA	72 C (52-92 C)	48 C	72 C (.28)	>95	eFigure 4B in Supplement 1
tDCS-resistant hallucinations											
LDLPFC	7^31,33,45,65,69,75,81^	242	38.2	14.1	1.66	2 mA	34 C (12 – 96 C)	50 C	95 C (.34)	>95	eFigure 5 in Supplement 1
**TMS for patients with treatment-resistant depression**											
HF-LDLPFC	26^18,20,21,23,25,27,28,30,32,35,43,44,47,56,83,84,87,88,90,91,107,111,112,114,121,124^	1166	43.1	13.75	2.8	13 Hz	21 570 (1250-60 000)	4416	12 374 (<.001); 13 214 (.02)^a^	90; >90	Figure 2A, eFigures 14 and 20 in Supplement 1
BLDLPFC	4^28,44,83,94^	178	47.6	13.75	2.75	10 Hz	25 056 (16 000-3000)	23 879	34 773 (.04	>95	Figure 2B
LF-RDLPFC	4^43,57,63,111^	102	48.8	12.5	2.5	2 Hz	1850 (1200-3000)	354	889 (.95)	>95	eFigure 6 in Supplement 1
**TMS for patients with major depressive disorder**											
HF-LDLPFC	5^42,57,70,110,113^	209	48.5	15	2.8	10 Hz	29 600 (8000-60 000)	3824	14 054 (.48); 21 948 (.98)	>85	Figure 3A, eFigure 15 in Supplement 1
LF-RDLPFC	2^61,67^	97	53.6	13	3	1 Hz	1560 (1200-1950)	1058	1835 (.001)	>95	Figure 3B
**tDCS for patients with treatment-resistant depression**											
LDLPFC	6^28,73,74,82,101,102^	351	43.5	12	3.14	1.9 mA	82.4 C (50.4-192.1 C)	31.2 C	48.2 C (<.001); 49.2 C (<.001)^a^	>95	Figure 3C, eFigure 17 in Supplement 1
**TMS for patients with bipolar depression**											
HF-LDLPFC	2^48,49^	42	43.6	12.5	2.5	20 Hz	2300 (1600-3000)	2338	5845 (.13)	95	eFigure 7 in Supplement 1
**tDCS for patients with bipolar depression**											
LDLPFC	2^74,103^	95	46.8	16	5	2.25 mA	226 C (192-259 C)	NA	NA	NA	eFigure 8 in Supplement 1
**TMS for patients with OCD**											
LF-RDLPFC	5^17,41,64,68,93^	138	30.5	11.6	9.6	1 Hz	13 560 (7200-20 000)	9429	18 923 (<.001)^a^	60	Figure 4A
LF-OFC	3^68,86,99^	85	37.7	11.6	2	1 Hz	12 000 (9000-15 000)	5001	13 679 (<.001)^a^	60	Figure 4B
HF-RDLPFC	2^41,78^	57	33.8	8	8	10 Hz	40 000 (20 000-60 000)	7854	20 715 (.53)	>95	eFigure 9 in Supplement 1
HF-LDLPFC	2^22,100^	58	29.5	12.5	2.5	15 Hz	13 500 (12 000-15 000)	2355	5516 (.72)	NA	eFigure 10 in Supplement 1
LF-SMA	4^19,50,79,92^	116	36.3	17	3.25	1 Hz	39 900 (12 000-90 000)	4262	10 766 (.50)	>95	eFigure 11 in Supplement 1
BLDLPFC	4^54,60,77,104^	87	31.3	12.5	2.5	17.5 Hz	7525 (7500-7600)	NA	NA	NA	NA
**TMS for PTSD**											
LF-RDLPFC	3^29,85,116^	49	45.5	11.6	2.3	1 Hz	15 333 (12 000-18 000)	12 811	17 495 (<.001)^a^	45	Figure 4C
HF-RDLPFC	2^16,29^	53	47.8	10	3	20 Hz	20 000 (16 000-24 000)	4484	11 234 ( .12)	95	eFigure 12 in Supplement 1
**tDCS for SUD**											
LDLPFC (CUD and MUD)	7^14,15,40,46,55,66,80^	242	35.8	7.3	2.7	1.87 mA	17.4 C (2.4-36.0 C)	3.4 C	9.6 C (<.001); 9.8 C (<.001)^a^	60	Figure 4D, eFigure 18 in Supplement 1
**iTBS for SUD**											
LDLPFC	4^36,37,108,109^	186	32.9	17.5	3.5	50 Hz	15 725 (9000-18 000)	3592	9724 (<.001)	70	Figure 4E

Abbreviations: BLDLPFC, bilateral dorsolateral prefrontal cortex; CUD, cocaine use disorder; ED_50_, median effective dose; ED_95_, 95% effective dose (or near-maximal effective dose); HF, high frequency; iTBS, intermittent theta-burst stimulation; LDLPFC, left dorsolateral prefrontal cortex; LF, low frequency; LTPJ, left temporoparietal junction; MUD, methamphetamine use disorder; OCD, obsessive-compulsive disorder; OFC, orbitofrontal cortex; PTSD, posttraumatic stress disorder; RDLPFC, right dorsolateral prefrontal cortex; SMA, supplementary motor area; SUD, substance use disorder; tDCS, transcranial direct current stimulation; TMS, transcranial magnetic stimulation.

^a^ Sensitivity analyses were conducted for these statistically significant results.

^b^ eFigure 9 in Supplement 1.

The characteristics of each study, including condition treated, stimulation type and site, and treatment strategy, are detailed in eTable 1 in Supplement 1 and briefly summarized here. Magnetic resonance imaging–guided neuronavigation was used in 6 studies on schizophrenia (17%), 6 studies in patients with a current depressive episode (12%), and 2 studies in patients with OCD (11%). For TMS studies, figure-eight coils were used in 76 (95%), and for tDCS studies, 29 used electrodes of 35 cm^2^ (79%). Furthermore, while mixed methods were used in studies of patients with schizophrenia to determine stimulation site, the 10-20 electroencephalographic system was mostly used for tDCS studies (29 [96%]), and the 5-cm rule in TMS studies for patients with depression (61 [77%]). The treatment strategy was augmentation or mixed for most studies, albeit some studies on depression and almost all studies on SUD.

### Dose-Response Meta-Analyses

#### Schizophrenia

**Figure 1.**
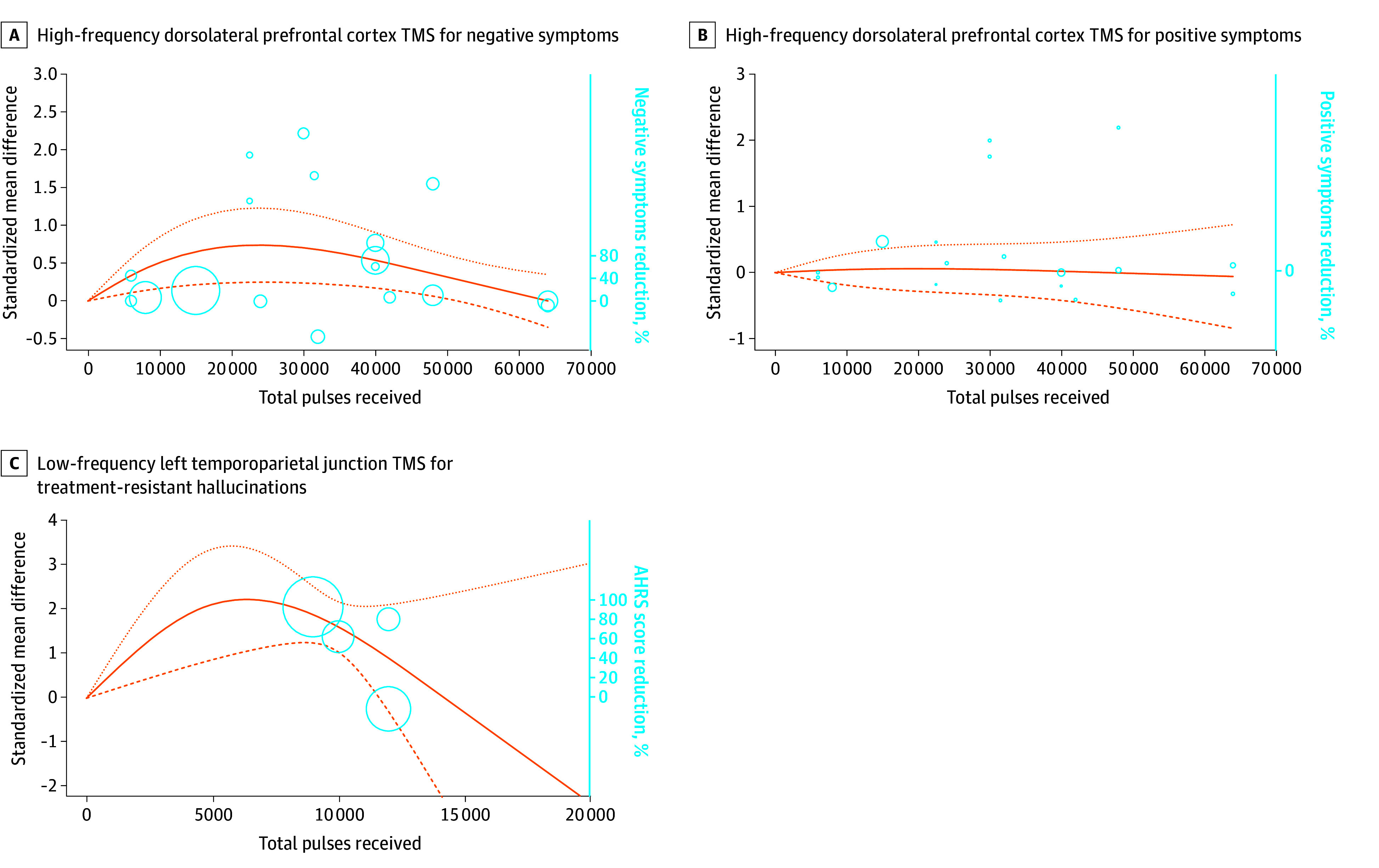
Dose-Response Curves of Transcranial Magnetic Stimulation (TMS) for Treating Schizophrenia The dose-response curves represent the standardized mean difference reduction of symptoms for the treatment arm compared with the sham arm. Circles represent the number of individuals per dose included. The dotted lines are 95% CIs. Knot locations are at the 25th, 50th, and 75th percentiles to anchor the curves. A. The maximum reduction of negative symptoms (95% effective dose) was reached at 21 695 total pulses (95% CI, 10 071-23 531 total pulses; mean [SD] duration, 3.9 [2.0] weeks; 14 studies^53,59,71,95,96,97,98,105,115,117,119,120,122,123^; 909 participants; χ^2^ = 9.35; *df* = 2; *P* = .009; *I*^2^ = 83%). B. Although with very high uncertainty, this safety analysis found no effect on positive symptoms (mean [SD] duration, 3.9 [2.0] weeks; 14 studies^53,59,71,95,96,97,98,105,115,117,119,120,122,123^; 909 participants; χ^2^ = 0.05; *df* = 2; *P* = .98; *I*^2^ = 75%). C. Although with very high uncertainty for high dose, a bell-shape curve was obtained (mean [SD] duration, of 1.7 [0.5] weeks; 5 studies^24,34, 53,91,122^; 159 participants; χ^2^ = 36.52 *df* = 2; *P* < .001; *I*^2^ = 95%). AHRS indicates Auditory Hallucination Rating Scale.

Fourteen sham-controlled studies delivered HF-LDLPFC TMS for schizophrenia for 909 participants over a mean (SD) duration of 3.9 (2.0) weeks.^53,59,71,95,96,97,98,105,115,117,119,120,122,123^ A significant dose-response association with a bell-shaped dose-response curve was obtained (χ^2^ = 9.35; *df* = 2; *P* = .009) (Figure 1A), peaking at 21 695 total pulses (95% CI, 19 971-23 531 total pulses), with considerable heterogeneity (*I*^2^ = 83%). Such bell-shaped curves suggest that a higher amount of total pulse stimulation is associated with less improvement of negative symptoms in the short term.

We conducted a safety analysis regarding outcomes associated with positive symptoms (Figure 1B). A flat curve was obtained, suggesting the absence of an effect on positive symptoms (χ^2^ = 0.05; *df* = 2; *P* = .98; *I*^2^ = 75%). For all analyses, the exploration of heterogeneity is reported in eTable 2 in Supplement 1.

Nonsignificant associations were found for 2 studies of BLDLPFC TMS for both positive and negative symptoms^26,38^ (eAppendix 1, eFigures 2 and 3 in Supplement 1). Nonsignificant associations were also found for LDLPFC tDCS for negative symptoms (eAppendix 1, eFigure 4A and B in Supplement 1).

We included 5 sham-controlled studies delivering low-frequency (LF) left temporoparietal junction (LTPJ) TMS for 159 participants over a mean (SD) duration of 1.7 (0.5) weeks.^24,33,52,89,118^A significant dose-response association was found with a bell-shaped curve (χ^2^ = 36.52; *df* = 2; *P* < .001) (Figure 1C) in the presence of considerable heterogeneity (*I*^2^ = 95%). The ED_95_ was reached at 5000 pulses. Nonsignificant associations were found for 7 studies of LDLPFC tDCS for treatment-resistant hallucinations^31,33,45,65,69,75,81^ (eAppendix 1, eFigure 5 in Supplement 1).

#### Current Depressive Episode

**Figure 2.**
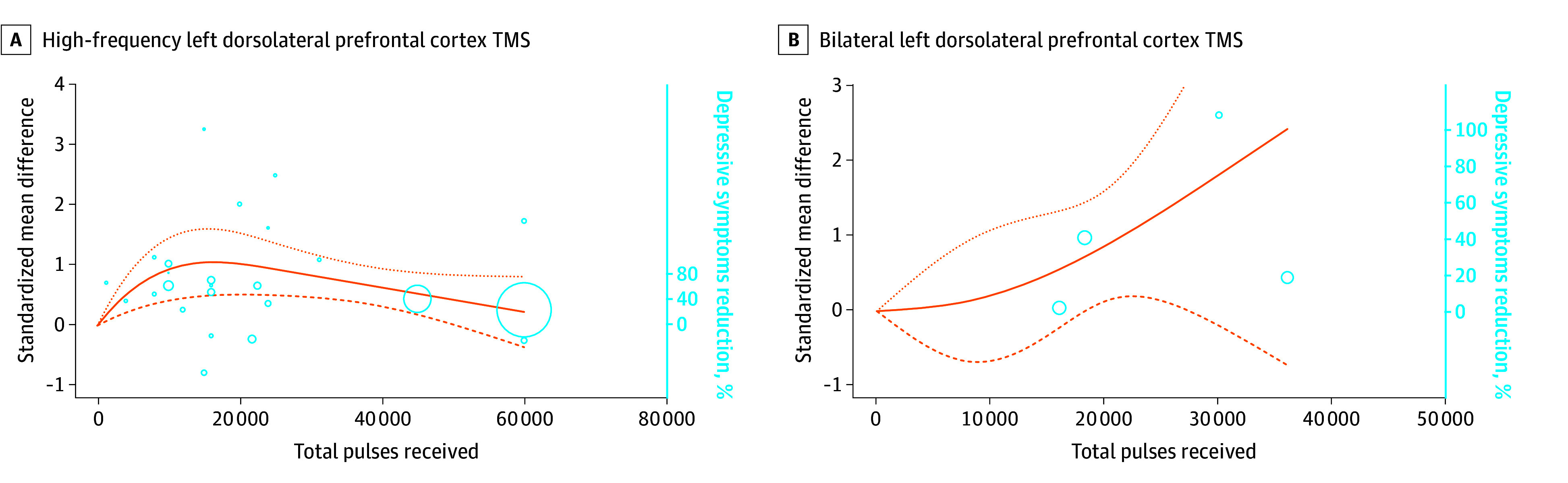
Dose-Response Curves of Transcranial Magnetic Stimulation (TMS) for Treating Treatment-Resistant Depression The dose-response curves represent the standardized mean difference reduction of symptoms for the treatment arm compared with the sham arm. Circles represent the number of individuals per dose included. The dotted lines are 95% CIs. Knot locations are at the 25th, 50th, and 75th percentiles to anchor the curves. A. Although with a moderate effect size, the maximum reduction of negative symptoms (95% effective dose) was reached at 12 374 total pulses (95% CI, 11 185-15 026 total pulses; mean [SD] duration, 2.8 [1.0] weeks; 26 studies^18,20,21,23,25,27,28,30,32,35,43,44,47,56,83,84,87,88,90,91,107,111,112,114,121,124^; 1166 participants; χ^2^ = 14.49; *df* = 2; *P* < .001; *I*^2^ = 90%). B. The maximum reduction of depressive symptoms (95% effective dose) was reached at 34 773 total pulses (95% CI, 32 256-36 521 total pulses; mean [SD] duration, 2.8 [0.5] weeks; 4 studies^28,44,83,94^; 178 participants; χ^2^ = 5.86; *df* = 2; *P* = .004; I^2^ = 95%).

Twenty-six sham-controlled studies delivered HF-LDLPFC TMS for 1096 participants with treatment-resistant depression^18,20,21,23,25,27,28,30,32,35,43,44,47,56,83,84,87,88,90,91,107,111,112,114,121,124^ The mean (SD) duration of trials was 2.8 (1.0) weeks. A significant dose-response association was found (χ^2^ = 14.49; *df* = 2; *P* < .001), with a bell-shaped curve suggesting that a higher dose than the ED_95_ is associated with less improvement of depressive symptoms (Figure 2A). The ED_95_ was reached at 12 374 total pulses (95% CI, 11 185-15 026 total pulses) in the presence of considerable heterogeneity (*I*^2^ = 90%).

Four studies delivered LF-BLDLPFC TMS for 178 patients with treatment-resistant depression.^28,44,83,94^ The mean (SD) duration of trials was 2.8 (0.5) weeks. A significant dose-response association was found (χ^2^ = 5.86; *df* = 2; *P* = .004) with an ascending curve (Figure 2B) and considerable heterogeneity (*I*^2^ = 95%). The ED_95_ was reached at 34 773 total pulses (95% CI, 32 256-36 521 total pulses). Nonsignificant associations were found in 4 studies of LF-RDLPFC TMS for treatment-resistant depression^43,57,63,111^ (eAppendix 1, eFigure 6 in Supplement 1).

**Figure 3.**
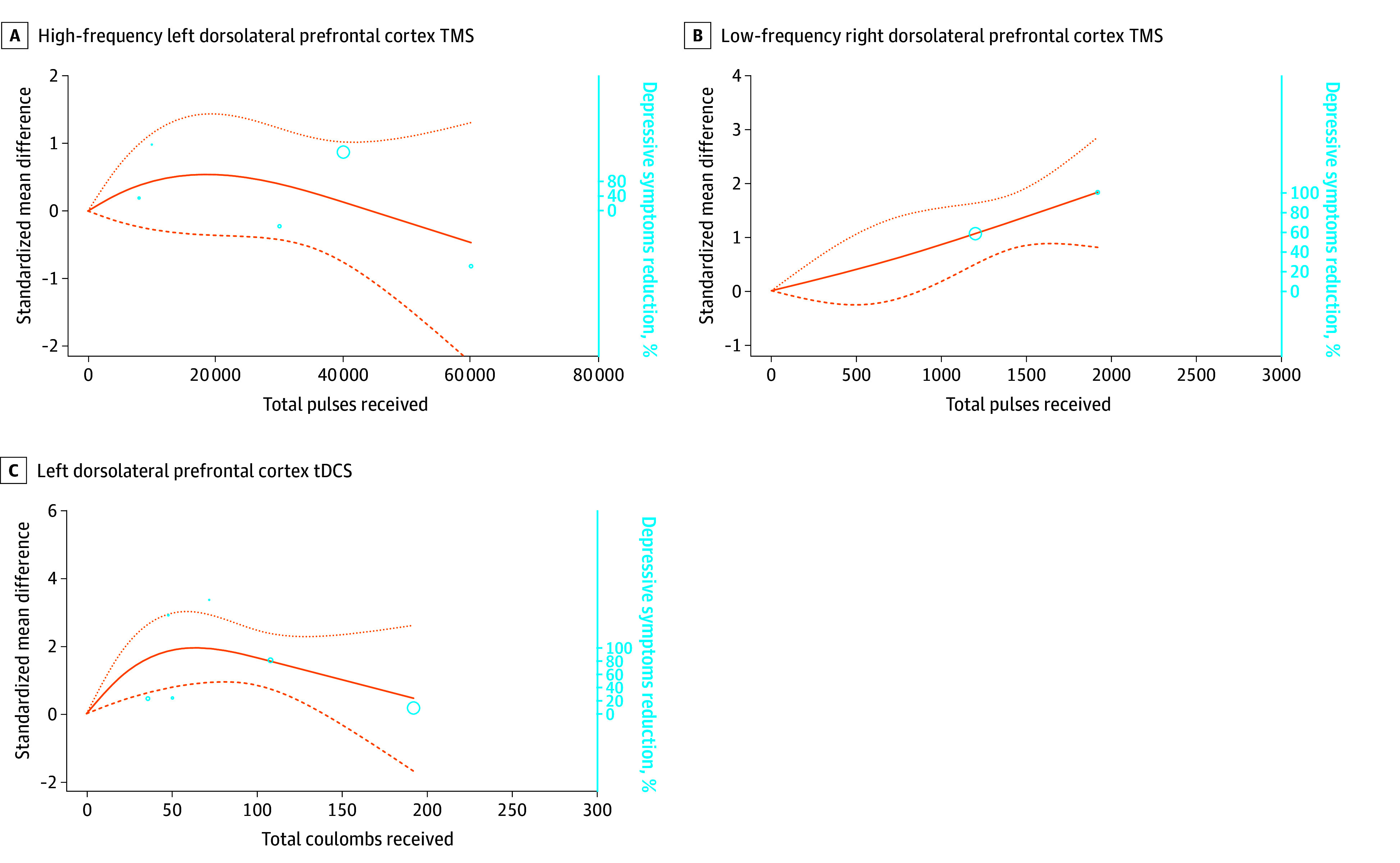
Dose-Response Curves of Transcranial Magnetic Stimulation (TMS) and Transcranial Direct Current Stimulation (tDCS) for Treating Major Depressive Disorder The dose-response curves represent the standardized mean difference reduction of symptoms for the treatment arm compared with the sham arm. Circles represent the number of individuals per dose included. The dotted lines are 95% CIs. Knot locations are at the 25th, 50th, and 75th percentiles to anchor the curves. A. The maximum improvement of depressive symptoms (95% effective dose [ED_95_]) was reached at 14 054 total pulses (95% CI, 9522-19 235 total pulses; mean [SD] duration, 2.8 [0.5] weeks; 5 studies^42,57,70,110,113^; 209 participants; χ^2^ = 1.46; *df* = 2; *P* = .48; *I*^2^ = 85%). B. The maximum improvement of depressive symptoms (ED_95_) was reached at 1835 total pulses (95% CI, 1721-1919 total pulses; mean [SD] duration, 2.8 [1.0] weeks; 2 studies^61,67^; 97 participants; χ^2^ = 25.67; *df* = 2; *P* = .001; *I*^2^ = 95%). C. The maximum improvement of depressive symptoms (ED_95_) was reached for a total of 48.2 coulombs (C) (95% CI, 35-55 C; mean [SD] duration, 3.1 [0.9] weeks; 6 studies^28,73,74,82,101,102^; 265 participants; χ^2^ = 14.56; *df* = 2; *P* < .001; *I*^2^ = 96%).

Five studies delivered HF-LDLPFC TMS for 209 participants with depression^42,57,70,110,113^ over a mean (SD) duration of 2.5 (0.5) weeks. A bell-shaped curve was obtained, with coherent results. However, since the curve passes through the abscissa line, the dose-response association was not statistically significant (Figure 3A). The ED_95_ was reached at 14 054 total pulses (95% CI, 9522-19 235 total pulses).

Two studies delivered LF-RDLPFC TMS for 97 participants with depression.^61,67^ The mean (SD) duration of trials was 2.8 (1.0) weeks. The dose-response association was statistically significant (χ^2^ = 25.67; *df* = 2; *P* = .001), with a straight ascending curve. Such curves suggest that the higher total amount of pulse was associated with a further decrease of symptoms (Figure 3B). The ED_95_ was reached for 1835 total pulses (95% CI, 1721-1919 total pulses) in the presence of considerable heterogeneity (*I*^2^ = 95%).

Six studies delivered LDLPFC tDCS for 351 participants with treatment-resistant depression.^28,73,74,82,101,102^ The mean (SD) trial duration was 3.1 (0.9) weeks. A significant association was found with a bell-shaped dose-response curve (χ^2^ = 14.56; *df* = 2; *P* < .001) (Figure 3C), with considerable heterogeneity (*I*^2^ = 96%), suggesting that the ED_95_ was reached for a total of 48 coulombs (C) (95% CI, 35-55 C).

Nonsignificant results were found for 2 studies that delivered HF-LDLPFC TMS^48,49^ for bipolar depression (eAppendix 1, eFigure 7 in Supplement 1). Nonsignificant results also were found for 2 studies that delivered LDLPFC tDCS^74,103^ for bipolar depression (eAppendix 1, eFigure 8 in Supplement 1).

#### Obsessive-Compulsive Disorder

**Figure 4.**
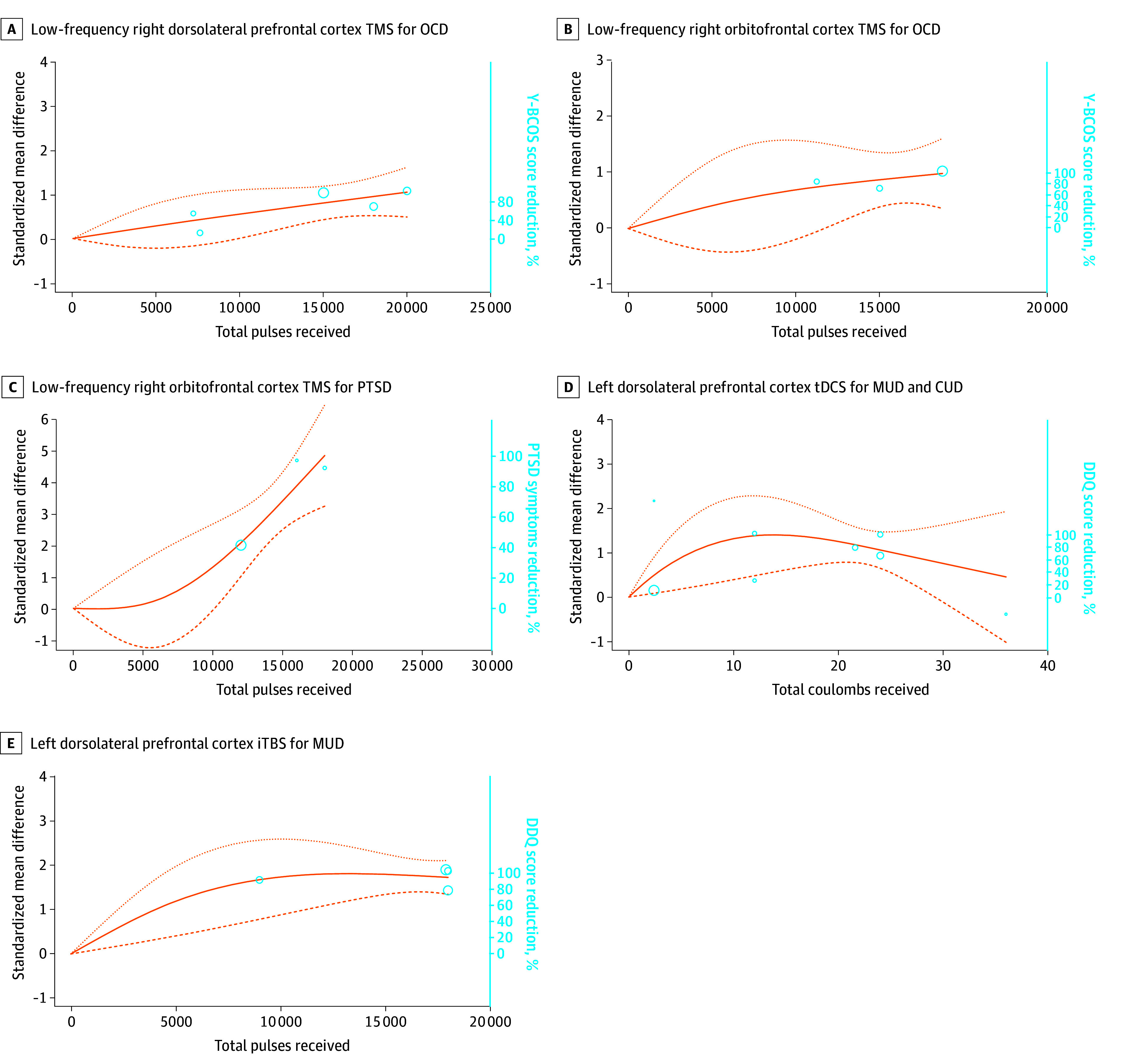
Dose-Response Curves for Transcranial Magnetic Stimulation (TMS) for Treating Obsessive-Compulsive Disorder (OCD) and Posttraumatic Stress Disorder (PTSD), Transcranial Direct Current Stimulation (tDCS) for Treating Methamphetamine Disorder (MUD) and Cocaine Use Disorder (CUD), and Intermittent Theta-Burst Stimulation (iTBS) for Treating MUD The dose-response curves represent the standardized mean difference reduction of symptoms for the treatment arm compared with the sham arm. Circles represent the number of individuals per dose included. The dotted lines are 95% CIs. Knot locations are at the 25th, 50th, and 75th percentiles to anchor the curves. A. The maximum improvement of OCD symptoms (95% effective dose [ED_95_]) was reached at 18 923 total pulses (95% CI, 7837-19 543 total pulses; mean [SD] duration, 9.6 [1.8] weeks; 5 studies^17,41,64,68,93^; 138 participants; χ^2^ = 20.65; *df* = 2l *P* < .001, *I*^2^ = 60%). B. The maximum improvement in Yale-Brown Obsessive-Compulsive Scale (Y-BCOS) scores (ED_95_) was reached at 13 679 total pulses (95% CI, 7117-14 734 total pulses; mean [SD] duration, 2.0 [1.0] weeks; 3 studies^68,86,99^; 85 participants; χ^2^ = 15.19; *df* = 2; *P* < .001; *I*^2^ = 60%). C. The maximum improvement in Desires for Drug Questionnaire (DDQ) scores (ED_95_) was reached at 17 495 total pulses (95% CI, 16 596-18 523 total pulses; mean [SD] duration, 2.3 [1.3] weeks; 3 studies^29,85,116^; 49 participants; χ^2^ = 54.15; *df* = 2; *P* < .001; *I*^2^ = 45%). D. The maximum improvement in DDQ scores (ED_95_) was reached for a total of 9.61 coulombs (C) (95% CI, 8.9-13.2 C; mean [SD] duration, 4.0 [2.1] weeks; 3 studies for MUD^14,15,40^ [151 participants], 3 studies for CUD^46,66,80^ [50 participants], and 1 study for alcohol use disorder^55^ [21 participants]; χ^2^ = 33.63; *df* = 2; *P* < .001; *I*^2^ = 60%). E. The maximum improvement DDQ scores (ED_95_) was reached at 9724 total pulses (95% CI, 7464-17 423 total pulses; mean [SD] duration, 3.5 [1.0] weeks; 4 studies^36,37,108,109^; 186 participants; χ^2^ = 92.82; *df* = 2; *P* < .001; *I*^2^ = 70%).

Five studies delivered LF-RDLPFC TMS for 138 participants with OCD,^17,41,64,68,93^ with a mean (SD) duration of 9.6 (1.8) weeks. A significant dose-response association (χ^2^ = 20.65; *df* = 2; *P* < .001) with an ascending curve was obtained (Figure 4A) in the presence of moderate heterogeneity (*I*^2^ = 60%). The ED_95_ was reached for 19 117 total pulses (95% CI, 7837-19 543 total pulses).

Three studies delivered LF TMS of the orbitofrontal cortex for 85 participants with OCD,^68,86,99^ with a mean (SD) duration of 2.0 (1.0) weeks. A significant dose-response association (χ^2^ = 15.19; *df* = 2; *P* < .001) with an ascending dose-response curve was obtained in the presence of moderate heterogeneity (*I*^2^ = 60%) (Figure 4B). The ED_95_ was reached at 13 679 total pulses (95% CI, 7117-14 734 total pulses).

Nonsignificant associations were found for 2 studies^41,78^ that delivered HF-RDLPFC TMS (eAppendix 1, eFigure 9 in Supplement 1) and 2 studies^22,100^ that delivered HF-LDLPFC TMS (eAppendix 1, eFigure 10 in Supplement 1) for OCD. Nonsignificant associations also were found for 4 studies that delivered LF supplementary motor area stimulation for OCD^19,50,79,92^ (eAppendix 1, eFigure 11 in Supplement 1).

#### Posttraumatic Stress Disorder

Three studies delivered LF-RDLPFC TMS for 49 participants with PTSD,^29,85,116^ with a mean (SD) duration of 2.3 (1.3) weeks. A significant dose-response association was found (χ^2^ = 54.15; *df* = 2; *P* < .001), with an ascending curve (Figure 4C). The ED_95_ was reached at 17 495 total pulses (95% CI, 16 596-18 523 total pulses) in the presence of moderate heterogeneity (*I*^2^ = 45%). Nonsignificant associations were found in 2 studies that delivered HF-RDLPFC TMS for PTSD^16,29^ (eAppendix 1, eFigure 12 in Supplement 1).

#### Substance Use Disorder

Seven studies delivered LDLPFC tDCS for 151 participants with methamphetamine use disorder,^14,15,40^ 50 participants with cocaine use disorder,^46,66,80^ and 21 participants with alcohol use disorder,^55^ with a mean (SD) duration of 2.7 (1.0) weeks. A bell-shaped curve showed a significant dose-response association (χ^2^ = 33.63; *df* = 2; *P* < .001) (Figure 4D). The ED_95_ was reached at 9.6 C (95% CI, 8.9-13.2 C) in the presence of moderate heterogeneity (*I*^2^ = 60%).

Four studies delivered LDLPFC iTBS for 186 participants with methamphetamine use disorders,^36,37,108,109^ for a mean (SD) duration of 3.5 (1.0) weeks. A significant dose-response association was found (χ^2^ = 92.82; *df* = 2; *P* < .001), with a curve that plateaued (Figure 4E). Such a curve suggests that the optimal reduction of craving score was reached, with an ED_95_ of 9724 total pulses (95% CI, 7464-17 423 total pulses), although in the presence of considerable heterogeneity (*I*^2^ = 70%) mostly due to low-dose studies (eAppendix 1, eTable 2 in Supplement 1).

### Sensitivity Analyses Excluding Studies Deemed at High Risk of Bias

We conducted sensitivity analyses excluding studies with an overall risk-of-bias assessment rated as high for curves with significant association (eTable 3 in Supplement 1). The results remained unchanged when excluding studies with high risk of bias (eAppendix 3, eFigures 13-18 in Supplement 1). Finally, we conducted an additional analysis of the frequency used in TMS studies, which is reported in eAppendix 4 and eFigures 19 and 20 in Supplement 1.

## Discussion

To our knowledge, this series of dose-response meta-analyses is the first to investigate the association of treatment using different doses (total pulses or coulombs) of TMS and tDCS, compared with sham, with core symptoms within a broad range of mental disorders. We discuss here the significant dose-response associations of TMS and tDCS protocols by disorder and, where possible, compare them with associations observed for pharmacologic treatments.

For TMS protocols targeting symptoms of schizophrenia, we observed bell-shaped curves for HF-LDLPFC TMS targeting negative symptoms and for LF-LTPJ TMS targeting treatment-resistant hallucinations. The findings suggest that beyond a certain threshold, further stimulation was associated with diminished symptom reduction. Notably, these observed dose-response associations were similar to those found in a recent series of dose-response meta-analyses for antipsychotic medications targeting schizophrenia symptoms.^9^

For protocols targeting symptoms of depression in patients with treatment-resistant depression, we observed bell-shaped curves for HF-LDLPFC TMS and LDLPFC tDCS, suggesting that beyond a certain threshold, further stimulation was associated with diminished symptom reduction. These curves were similar to those recently observed in a series of dose-response meta-analyses for selective serotonin reuptake inhibitors for depressive symptoms.^125^ By contrast, we observed ascending curves for BLDLPFC TMS and LF-RDLPFC TMS protocols for patients with treatment-resistant depression and patients with depression, respectively, with protocols delivering a greater number of total pulses associated with an increased reduction of depressive symptoms. We also observed an ascending curve for LF-RDLPFC TMS targeting symptoms of PTSD. Future studies could therefore explore the feasibility and efficacy of TMS protocols for these mental disorders with a greater number of total pulses than were included in the current analysis. Nevertheless, a recent study in 7215 patients with depression observed that in clinical practice, patients with longer than standard courses typically show less initial improvement and a more gradual trajectory and that meaningful benefit accrues with treatment beyond 36 sessions.^126^

For protocols targeting symptoms of OCD, we observed curves starting to plateau for LF-RDLPFC TMS and LF-OFC TMS. These curves suggest that beyond a certain threshold, further stimulation may not lead to an improved reduction in symptoms. This finding is different from the dose-response association found in a previous meta-analysis investigating selective serotonin reuptake inhibitors for OCD symptoms, which reported an ascending curve, albeit with higher doses associated with an increased side effect burden.^127^

Finally, for protocols targeting craving symptoms in methamphetamine use disorder, we observed a bell-shaped curve for LDLPFC tDCS, with stimulation beyond a certain threshold resulting in diminished symptom reduction. For LDLPFC iTBS, we observed a plateauing curve, indicating that beyond a certain threshold, further stimulation may not lead to more reductions in cravings.

While the exact mechanisms of NIBS are still unknown, recent research has suggested that increasing the number of TMS pulses beyond a certain point may saturate structural network reorganization,^128^ which may provide some explanation for the bell-shaped and plateauing dose-response associations we observed in some TMS protocols. However, TMS protocols that deliver stimulation in intermittent patterns (eg, iTBS) may still show additive outcomes for symptom reduction when delivering a high number of pulses.^128^ Thus, more studies are needed to understand the treatment mechanisms of NIBS and how they might explain dose-response associations.

### Limitations

This study has several limitations. First, some analyses had a limited participant pool, resulting in nonsignificant dose-response curves for specific protocols, such as LF-LTPJ TMS for treatment-resistant hallucinations or LF-RDLPFC TMS for major depressive disorder. This limitation stemmed from a restricted number of enrolled participants. Our analysis suggests a minimum of 150 participants is necessary to establish reliable dose-response models for each stimulation type.^13^ While we included a substantial number of trials and participants for treatment-resistant depression and schizophrenia, particularly in TMS studies (2075 participants), robust dose-response models for other mental disorders necessitate additional trials.

Another limitation is the omission of various NIBS parameters in the included trials (eg, trial duration, stimulation intensity, initial symptom severity). Participant-specific factors such as age, sex,^129^ duration of episode,^130^ or previous response to electroconvulsive therapy may also be associated with treatment response. Specific symptoms, such as cognitive symptoms of schizophrenia, were also not analyzed. Indeed, due to the recommendation of having a minimum of 10 studies per regressor in meta-regression analyses^131^ and considering the inconsistent reporting of data on potential regressors across studies, we were unable to investigate the intended regressors. Furthermore, selective participant enrollment was lacking in most trials, as exemplified by only 2 studies focusing on negative symptoms of schizophrenia that selected participants with predominantly negative symptoms.^117,120^

Inconsistencies in reporting inclusion criteria and definitions of treatment resistance further contribute to the limitations, except for trials involving patients with treatment-resistant hallucinations. The absence of magnetic resonance imaging–based neuronavigation, used in only a few trials with conflicting results, is another limitation.^89,118^ Additionally, the lack of available data in the retrieved studies prevented the establishment of a dose-response association for adverse effects. Finally, while there is preliminary evidence on the outcomes of NIBS in autism spectrum disorder, borderline personality, attention-deficit/hyperactivity disorder, insomnia, and specific SUDs, the limited number of retrieved publications and the important heterogeneity within protocols limited us in approaching optimal efficacy for symptom improvement. Future RCTs exploring multiple doses of neurostimulation will be needed to address these gaps and expand our understanding across disorders.

## Conclusions

The findings of this systematic review and dose-response meta-analysis contribute to the understanding of optimal stimulation parameters across disorders and brain areas in the field of neuromodulation. Future research should address the identified limitations and further explore the optimal dose and potential adverse effects in longer-term trials.

## Notes

Supplement 1. **eAppendix 1.** Nonsignificant Findings **eFigure 1.** PRISMA Flow Diagram **eAppendix 2.** List of Excluded Studies **eFigure 2**. Dose-Response Curve of BLDLPFC TMS for Negative Symptoms **eFigure 3.** Dose-Response Curve of BLDLPFC TMS for Positive Symptoms **eFigure 4.** Dose-Response Curve of LDLPFC tDCS Effect on Negative and Positive Symptoms of Schizophrenia **eFigure 5.** Dose-response Curve of LDLPFC tDCS Treatment-Resistant Hallucinations in Patients With Schizophrenia **eFigure 6**. Dose-Response Curve of LF-RDLPFC TMS for Patients Treatment-Resistant Depression **eFigure 7.** Dose-response Curve of HF-LDLPFC TMS for Patients With Bipolar Depression **eFigure 8.** Dose-Response Curve of LDLPFC tDCS for Patients With Bipolar Depression **eFigure 9.** Dose-Response Curve of HF-RDLPFC TMS for Patients With OCD **eFigure 10.** Dose-Response Curve of HF-LDLPFC rTMS for Patients With OCD **eFigure 11.** Dose-Response Curve of LF-SMA rTMS for Patients With OCD **eFigure 12.** Dose-Response Curve of HF-RDLPFC rTMS for Patients With PTSD **eAppendix 3**. Overall Summary of Sensitivity Analyses **eFigure 13.** Sensitivity Analysis Excluding High-Risk Bias Studies Focusing on HF-LDLPFC TMS Total Pulses Received for Patients With Schizophrenia **eFigure 14.** Sensitivity Analysis Excluding High-Risk Bias Studies Focusing on HF-LDLPFC TMS Total Pulses Received for Patients With Treatment-Resistant Depression **eFigure 15.** Sensitivity Analysis Excluding High-Risk Bias Studies Focusing on HF-LDLPFC TMS Total Pulses Received for Patients With Major Depressive Disorder **eFigure 16.** Sensitivity Analysis Excluding High-Risk Bias Studies Focusing on LDLPFC tDCS for Patients With SUD **eFigure 17.** Sensitivity Analysis on LDLPFC tDCS for Patients With Resistant Depression **eFigure 18.** Sensitivity Analysis for Patients Focusing on RDLPFC tDCS Received for Patients With SUD (MUD and CUD) **eAppendix 4.** Additional Analyses Regarding the Frequency Used in TMS Studies **eFigure 19.** Sensitivity Analysis on HF-LDLPFC TMS Frequencies for Patients With Schizophrenia **eFigure 20.** Sensitivity Analysis on HF-LDLPFC TMS Frequencies Received for Patients With Resistant Depression **eTable 1.** Study Characteristics of Included Randomized Controlled Trials **eTable 2.** Heterogeneity Assessments With the Variance-Partition-Coefficient (VPC) for the Primary Outcome **eTable 3.** Risk-of-Bias Assessments **eReferences.** List of All Included Studies

Supplement 2.
Data Sharing Statement
