# Errors in Byline and Author Affiliations

**DOI:** 10.1001/jamanetworkopen.2024.24292

**Published:** 2024-07-03

In the Original Investigation “Transcranial Magnetic Stimulation and Transcranial Direct Current Stimulation Across Mental Disorders: A Systematic Review and Dose-Response Meta-Analysis,”^1^ published May 22, 2024, the affiliations for Michel Sabé and Matthew Garner were incorrect. The correct affiliations for Dr Sabé are the Division of Adult Psychiatry, Department of Psychiatry, University Hospitals of Geneva, Thonex, Switzerland, and Faculty of Medicine, University of Geneva, Geneva, Switzerland; the correct affiliations for Dr Garner are the Centre for Innovation in Mental Health, School of Psychology, University of Southampton, Southampton, United Kingdom, and Clinical and Experimental Sciences (CNS and Psychiatry), Faculty of Medicine, University of Southampton, Southampton, United Kingdom. Additionally, in the byline, Joshua Hyde’s degree has been corrected from PhD to BSc, and Catharina Cramer’s degree has been corrected from MD to MSc. Finally, in the byline, Dr Eberhard’s name and degree have been corrected from Antonia Eberhard, MD, to Antonia-Leonie Eberhard, MSc. This article has been corrected.^1^
