# Supplementary material for: Transcranial Magnetic Stimulation and Transcranial Direct Current Stimulation Across Mental Disorders: A Systematic Review and Dose-Response Meta-Analysis
Source: JAMA Netw Open. 2024 May 22;7(5):e2412616. doi: 10.1001/jamanetworkopen.2024.12616 (PMC11112448; doi:10.1001/jamanetworkopen.2024.12616)
Supplement: Supplement 1. — eAppendix 1. Nonsignificant Findings eFigure 1. PRISMA Flow Diagram eAppendix 2. List of Excluded Studies eFigure 2. Dose-Response Curve of BLDLPFC TMS for Negative Symptoms eFigure 3. Dose-Response Curve of BLDLPFC TMS for Positive Symptoms eFigure 4. Dose-Response Curve of LDLPFC tDCS Effect on Negative and Positive Symptoms of Schizophrenia eFigure 5. Dose-response Curve of LDLPFC tDCS Treatment-Resistant Hallucinations in Patients With Schizophrenia eFigure 6. Dose-Response Curve of LF-RDLPFC TMS for Patients Treatment-Resistant Depression eFigure 7. Dose-response Curve of HF-LDLPFC TMS for Patients With Bipolar Depression eFigure 8. Dose-Response Curve of LDLPFC tDCS for Patients With Bipolar Depression eFigure 9. Dose-Response Curve of HF-RDLPFC TMS for Patients With OCD eFigure 10. Dose-Response Curve of HF-LDLPFC rTMS for Patients With OCD eFigure 11. Dose-Response Curve of LF-SMA rTMS for Patients With OCD eFigure 12. Dose-Response Curve of HF-RDLPFC rTMS for Patients With PTSD eAppendix 3. Overall Summary of Sensitivity Analyses eFigure 13. Sensitivity Analysis Excluding High-Risk Bias Studies Focusing on HF-LDLPFC TMS Total Pulses Received for Patients With Schizophrenia eFigure 14. Sensitivity Analysis Excluding High-Risk Bias Studies Focusing on HF-LDLPFC TMS Total Pulses Received for Patients With Treatment-Resistant Depression eFigure 15. Sensitivity Analysis Excluding High-Risk Bias Studies Focusing on HF-LDLPFC TMS Total Pulses Received for Patients With Major Depressive Disorder eFigure 16. Sensitivity Analysis Excluding High-Risk Bias Studies Focusing on LDLPFC tDCS for Patients With SUD eFigure 17. Sensitivity Analysis on LDLPFC tDCS for Patients With Resistant Depression eFigure 18. Sensitivity Analysis for Patients Focusing on RDLPFC tDCS Received for Patients With SUD (MUD and CUD) eAppendix 4. Additional Analyses Regarding the Frequency Used in TMS Studies eFigure 19. Sensitivity Analysis on HF-LDLPFC TMS Frequencies for Patients With Schizop [file jamanetwopen-e2412616-s001.pdf]

Supplemental Online Content

Sabé M, Hyde J, Cramer C, et al. Transcranial magnetic stimulation and transcrantial direct current stimulation across mental disorders: a systematic review and dose-response meta-analysis. *JAMA Netw Open*. 2024;7(5):e2412616. doi:10.1001/jamanetworkopen.2024.12616

eAppendix 1. Nonsignificant Findings

eFigure 1. PRISMA Flow Diagram

eAppendix 2. List of Excluded Studies

eFigure 2. Dose-Response Curve of BLDLPFC TMS for Negative Symptoms

eFigure 3. Dose-Response Curve of BLDLPFC TMS for Positive Symptoms

eFigure 4. Dose-Response Curve of LDLPFC tDCS Effect on Negative and Positive Symptoms of Schizophrenia

eFigure 5. Dose-Response Curve of LDLPFC tDCS Treatment-Resistant Hallucinations in Patients With Schizophrenia

eFigure 6. Dose-Response Curve of LF-RDLPFC TMS for Patients Treatment-Resistant Depression

eFigure 7. Dose-Response Curve of HF-LDLPFC rTMS for Patients With Bipolar Depression

eFigure 8. Dose-Response Curve of LDLPFC tDCS for Patients With Bipolar Depression

eFigure 9. Dose-Response Curve of HF-RDLPFC rTMS for Patients With OCD

eFigure 10. Dose-Response Curve of HF-LDLPFC rTMS for Patients With OCD

eFigure 11. Dose-Response Curve of LF-SMA rTMS for Patients With OCD

eFigure 12. Dose-Response Curve of HF-RDLPFC rTMS for Patients With PTSD

eAppendix 3. Overall Summary of Sensitivity Analyses

eFigure 13. Sensitivity Analysis Excluding High-Risk Bias Studies Focusing on HF-LDLPFC TMS Total Pulses Received for Negative Symptoms Score of Patients With Schizophrenia

eFigure 14. Sensitivity Analysis Excluding High-Risk Bias Studies on HF-LDLPFC TMS Total Pulses Received for Patients With Treatment-Resistant Depression

eFigure 15. Sensitivity Analysis Excluding High-Risk Bias Studies Focusing on HF-LDLPFC TMS Total Pulses Received for Patients With Major Depressive Disorder

eFigure 16. Sensitivity Analysis Excluding High-Risk Bias Studies on LDLPFC tDCS for Patients With SUD

eFigure 17. Sensitivity Analysis on LDLPFC tDCS for Patients With Resistant Depression

eFigure 18. Sensitivity Analysis for Patients Focusing on RDLPFC tDCS Received for Patients With SUD (MUD and CUD)

eAppendix 4. Additional Analyses Regarding the Frequency Used in TMS Studies

eFigure 19. Sensitivity Analysis on HF-LDLPFC TMS Frequencies for Patients With Schizophrenia

© 2024 Sabé M et al. *JAMA Network Open.*

eFigure 20. Sensitivity Analysis on HF-LDLPFC TMS Frequencies for Patients With Resistant Depression

eTable 1. Study Characteristics of Included Randomized Controlled Trials

eTable 2. Heterogeneity Assessments With the Variance-Partition-Coefficient (VPC) for the Primary Outcome

eTable 3. Risk-of-Bias Assessments

eReferences. List of All Included Studies

This supplemental material has been provided by the authors to give readers additional information about their work.

eAppendix 1. Nonsignificant Findings

1. Studies for patients with schizophrenia

1.1. BLDLPFC TMS for negative symptoms of schizophrenia

Two sham-controlled studies delivered BLDLPFC TMS (n= 57), over a mean duration of 3.5 weeks. The confidence interval was wide with no dose-response association for both analysis on negative and positive symptoms (p>0.93) (eFigure 1, eFigure 2, eTable 2).

1.2. LDLPFC tDCS for negative symptoms of patients with schizophrenia

Five sham-controlled studies delivering LDLPFC tDCS were included (n=142), with a mean duration of 1.8 weeks. No dose-response association was found for both negative symptoms (p=0.218)(eFigure 3.a) and the safety analysis on positive symptoms (p=0.331)(eFigure 3.b). Both curves plateaued in presence of a considerable heterogeneity (I^2>95%)(eTable 2).

1.3. LDLPFC tDCS for patients with treatment-resistant hallucinations

LDLPFC tDCS for treatment-resistant hallucinations in patients with schizophrenia were found in 6 sham-controlled studies (n=242). The mean duration of trial was 2 weeks. The dose-response association was not statically significant (p=0.336)(eFigure 4).

2. Studies for patients with depression

2.1. LF-RDLPFC TMS for patients with treatment-resistant depression

Furthermore, 4 sham-controlled studies delivered LF-RDLPFC TMS for patients with treatment-resistant depression (n=102). The mean duration of trial was 2.5 weeks. In presence of a high uncertainty and a considerable heterogeneity (I^2=95%)(eTable 2), statistical significance was not reached (p=0.944)(eFigure 5). The curve obtained was flat.

3.3. Studies for patients with bipolar depression

3.1. BLDLPFC TMS for patients with bipolar depression

Three studies included patients with bipolar depression. One study delivered stimulation to BLDLPFC TMS and two studies from a same team to the HF-LDLPFC TMS (n=42). For HF-LDLPFC TMS, the mean duration of trial was 2.5 weeks. The obtained curve was bell-shaped (eFigure 6), suggesting higher dose are not effective on symptoms reduction. However, the dose-response association was not significant, and a considerable uncertainty and heterogeneity (I^2=95%). The ED95 was reached for a total amount of pulse of 5845.

3.2. LDLPFC tDCS for patients with bipolar depression

Two studies delivered LDLPFC tDCS for patients with bipolar depression. The mean duration of trial was 5 weeks (n=95). The inversed bell-shaped dose-response curve obtained was not significant, however, the results could suggest that higher total coulomb dose received could impact depressive symptoms (eFigure 7).

4. Studies for patients with OCD

4.1. HF-RDLPFC TMS for patients with OCD

Two studies applied HF-RDLPFC TMS for patients with OCD (n=57), with a mean duration of 8 weeks. Although a bell-shaped curve was obtained (eFigure 8) with a ED95% at 20715 total pulses, no dose response association was found (p=0.528), as both uncertainty and heterogeneity were considerable (I^2>95%).

4.2. BLDLPFC and HF-LDLPFC for patients with OCD

For BLDLPFC, although 4 studies were available, all studies used the same total amount of pulse, therefore no dose-response curve was possible. For HF-LDLPFC, only 2 studies were retained, with no effect for one study, which prevent to obtain a coherent curve (eFigure 9).

© 2024 Sabé M et al. *JAMA Network Open.*

4.3. LF-SMA TMS for patients with OCD

Four studies applied LF-SMA TMS for patients with OCD (n=116) with a mean duration of 3.25 weeks. The dose-response curve was not interpretable (eFigure 10) since one study (P. V. O. Gomes et al., 2012) presented an important effect size on a very limited sample size of patients (22 patients). The exclusion of this study showed a flat curve with no effect of TMS over LF-SMA for OCD (Fig S9.p)

5. Studies for patients with PTSD

5.1. HF-RDLPFC for PTSD

Two studies applied HF-RDLPFC for PTSD (n=53), with a mean duration of 3 weeks. No significant dose-response association was found (p=0.115)(I^2=95%)(eFigure 11).

eFigure 1. PRISMA Flow Diagram

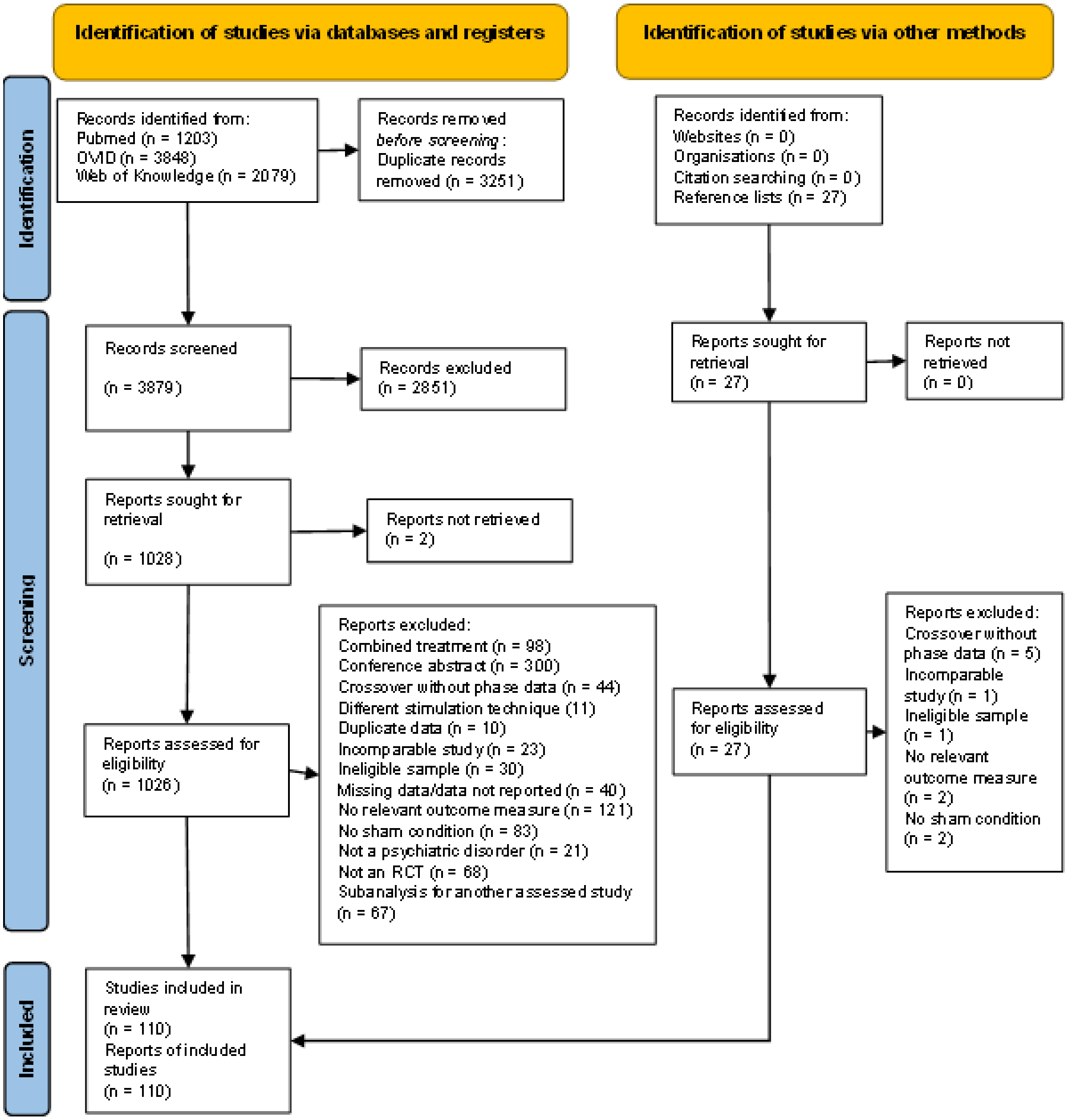

eAppendix 2. List of Excluded Studies

© 2024 Sabé M et al. *JAMA Network Open.*

Of importance, studies on patients with austism spectrum disorder, borderline disorder, ADHD, and patients with insomina were not included in our work. The paucity of studies, the variety of stimulation site, and the heterogenity of used scales was among the limitations that contibuted to this decision.

Moreover, several studies could not be included due to the absence of mention of the total pulse, or the total coulomb received, and the paucity of similar stimulated sites.

1.Retained studies presenting some particularity:

Studies including patients with predominant negative symptoms: (Wobrock et al. 2015)(Zaho et al. 2014)
Studies including patients with acute symptoms: (O'Reardon et al. 2007)
Studies including patients that are medication free: (Januel et al. 2006)
Studies only including female subjects: (Asl et al. 2022)
Studies only including war veterans: (Guan et al. 2020)
Studies including patients with ultra-resitants schizophrenia: (Lindemayer at al. 2019)

All of these studies were subjects to a leave-one-out analyses, that showed no signficant impact on our analyses.

2.Studies not retained (N=28):
Asku et al. (2022) doi: 10.1111/pcn.13378
Reason: Only study applying DLPFC tDCS for panic disorder

Balzus et al. (2022) doi: 10.1016/j.nicl.2022.103113
Reason: No report of our outcomes

Blumberger et al. (2012) doi : 10.3389/fpsyt.2012.00074
Reason: Unsufficient data to conduct meta-analysis

Cheng et al. (2022) doi: 10.1007/s00406-022-01523-4
Reason : Only study applying bilateral dorsomedial prefrontal cortex i TBS stimulation for patients with magnetic-stimulation-resistant major depression

Chou et al. (2022) doi: /10.1111/pcn.13524
Reason : Only study applying DLPFC bilateral theta-burst stimulation for patients with major depressive disorder

Hawken et al. (2016) doi: 10.3390/ijms17030420
Reason: Study applying rTMS stimulation on the SMA in patinets with OCD. The stimulation parameters of this study are particular. The population differs between both study sites. The sample of included patients are limited. Furtermore, many limitations are found with high risks of bias. We decided not to include this study.

Fineberg et al. (2023) doi: 10.1016/j.comppsych.2023.152371
Reason: Only study applying tDCS stimulation for patients with OCD at the SMA

Lee et al. (2022) doi : 10.3389/fpsyt.2022.969199
Reason : Only stdy applying home-based tDCS for patients with bipolar depressive episodes

Li et al. (2022) doi : 10.1503/jpn.210134
Reason: Only study applying DLPFC theta-burst stimulation for generalized anxiety disorder

Li et al. (2022) doi : 10.3389/fpsyt.2022.851908

© 2024 Sabé M et al. *JAMA Network Open.*

Reason: Insufficient data to conduct a meta-analysis

Liang et al. (2018) doi: 10.1007/s12264-018-0205-y
Reason: there is no precise data on the craving score in this study

Jabanbaksh et al. (2023) doi: 10.1016/j.ajp.2022.103384
Reason: Only study applying LF-LDLPFC rTMS stimulation for patients with resistant OCD

Kumar et al. (2022) doi: 10.1097/YCT.0000000000000820
Reason : Only study applying DLPFC high-definition tDCS for patients with opiod use-disorder

Mallik et al. (2022) doi: 10.1097/YCT.0000000000000870
Reason : Only study applying continuous theta-burst ''intensive'' stimulation in patinets with acute-phase bipolar depression

Mak et al. 2022 (2022) doi: 10.1186/ s40345-021-00245-1.
Reason: Only study applying LF in medication-resistant patients with bipolar depression

Moeller et al. (2022) doi: 10.1038/s41537-022-00224-0
Reason: Only study for this stimulation site that include patients with nicotine addiciton

Mukherjee et al. (2022) doi: /10.1017/S1092852922000980
Reason: Only study proposing theta-burst stimulation for OCD patients at the SMA

Tyagi et al. (2022) doi: 10.1016/j.ajp.2022.103176
Reason: Only study applying bilateral TPC theta-nurst stimulation for patients with resistant hallucinations

Oh et al. (2022) doi : 10.9758/cpn.2022.20.1.87
Reason: Only study where patients with Major depressive disorder self-administered tDCS treatment

Soleimani et al. (2022) doi: 10.3389/fnsys.2022.956315.
Reason: Insufficient data to conduct a meta-analysis

Su, et al. (2022) doi: 10.3389/fpsyt.2022.873057
Reason: this study include a specific population of war veterans that are also long-term hospitalized patients. Futhermore, the mean change observed is in favor of the placebo group, which is not seen in any of the other 14 studies retained in the studies in patients with schizophrenia.

Tavares et al. (2021) doi : 10.1038/s41386-021-01080-9
Reason : Only study applying theta-burst stimulation for patients with mixed depression

Yu et al. (2022) doi: 10.1016/j.jpsychires.2022.05.014
Reason: Insufficient data to conduct a meta-analysis

Yuan et al. (2020) doi: 10.1001/jamanetworkopen.2020.0910
Reason: Only LF stimulation study for this site of stimulation and with patients with Methamphetamine addiction

Yuan et al. (2020) doi: 10.3389/fpsyt.2020.00210
Reason: Only iTBS stimulation study for patients with Alcohol use disorder

© 2024 Sabé M et al. *JAMA Network Open.*

Zeng et al. (2022) doi: 10.1016/j.comppsych.2022.152332
Reason : Only infralow-frequency TMS study for generalized anxiety disorder

Zengin et al. (2022) doi: 10.1016/j.biopsych.2007.01.018
Reason: Insufficient data to conduct a meta-analysis

Zhang et al. (2023) doi: 10.1016/j.jad.2022.12.007
Reason : Only study applying high-definition tDCS stimulation for patients with bipolar depression

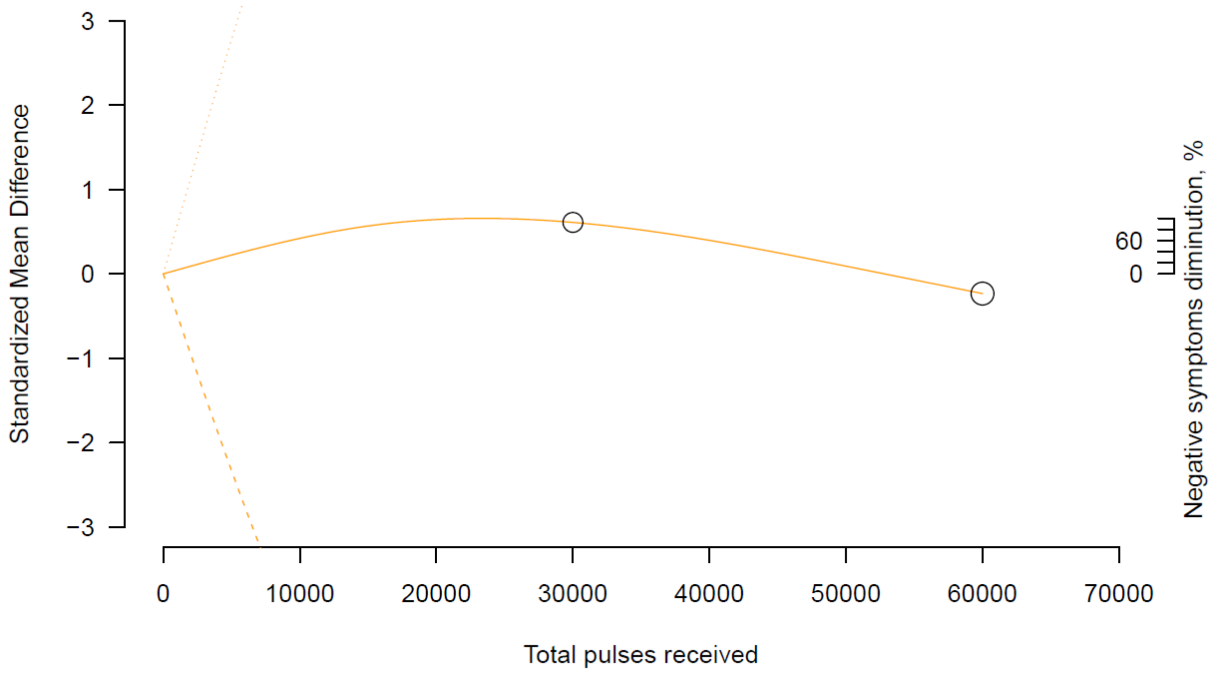

eFigure 2. Dose-Response Curve of BLDLPFC TMS for Negative Symptoms ($\chi 2$= 0.032 (df= 2), p= 0.984, I^2= 95%). Although with a low effect size and a very high uncertainty, the maximum reduction of negative symptoms (95%) was reached for a total pulse of 18207 (95CI%: n.a.), N=2, n=57; mean duration of 3.5 weeks.

© 2024 Sabé M et al. *JAMA Network Open.*

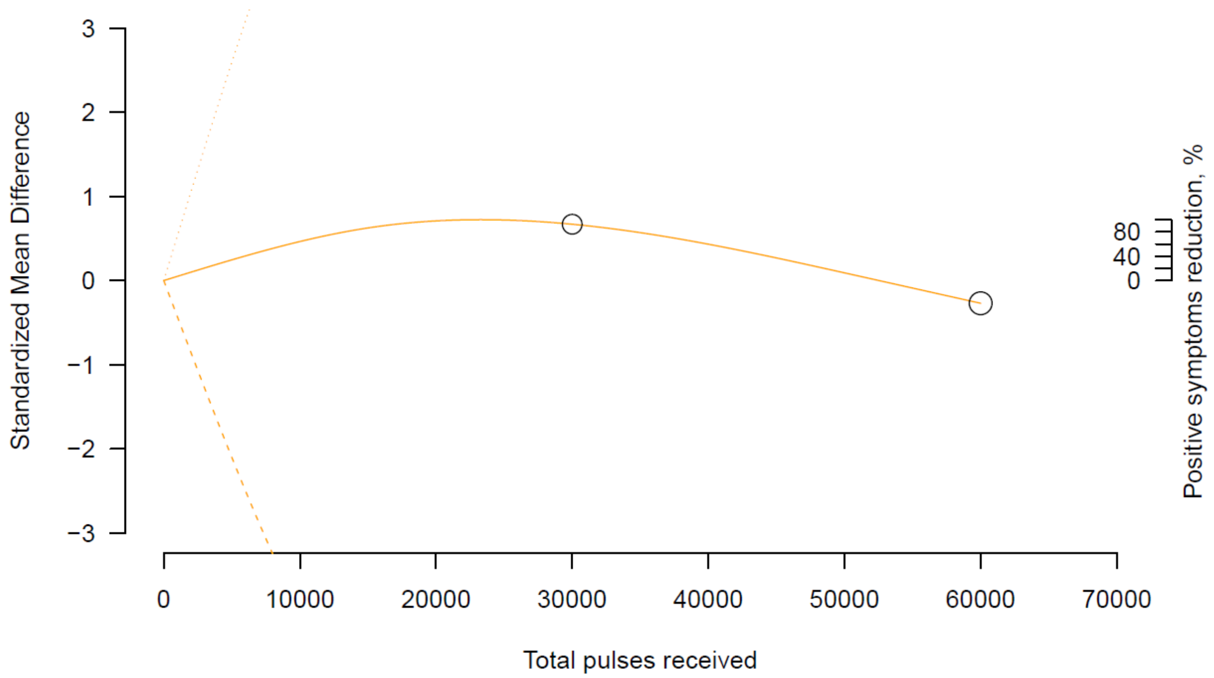

eFigure 3. Dose-Response Curve of BLDLPFC TMS for Positive Symptoms (χ2= 0.043 (df = 2), p= 0.973, I^2= 95%).
Although with a low effect size and a very high uncertainty, the maximum reduction of negative symptoms (95%) was reached for a total pulse of 18159 (95CI%: n.a.), N=2, n=57; mean duration of 3.5 weeks.

© 2024 Sabé M et al. *JAMA Network Open.*

eFigure 4.a

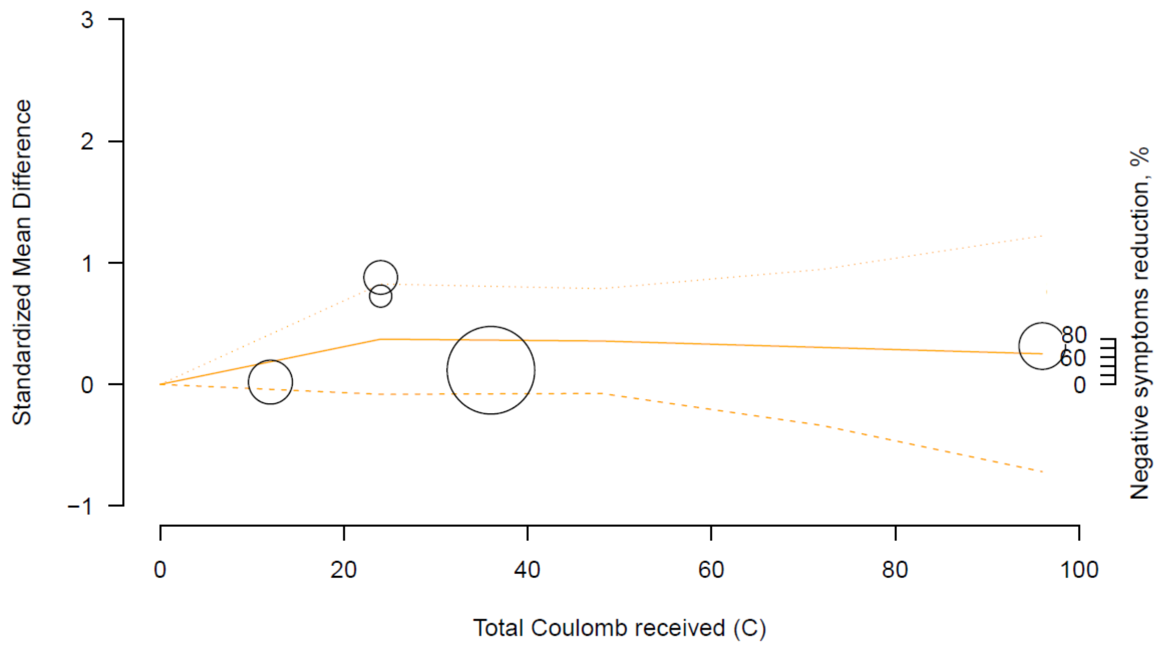

eFigure 4.b

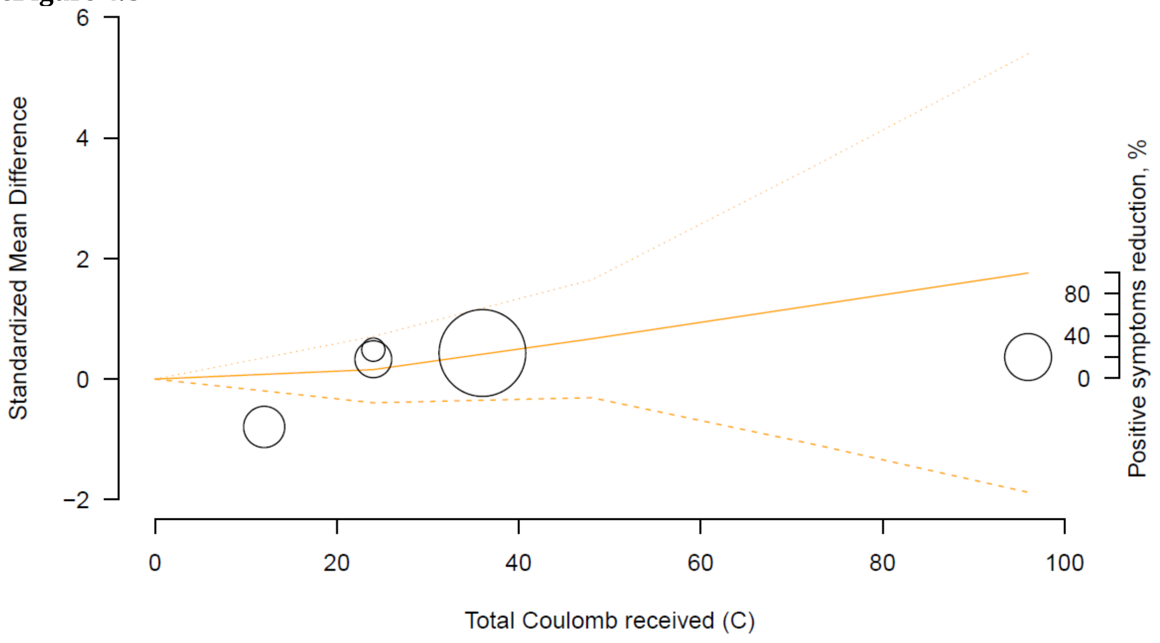

eFigure 4. Dose-Response Curve of LDLPFC tDCS Effect on Negative and Positive Sympotms of Schizophrenia

eFigure 4.a Dose-response curve of LDLPFC tDCS effect on negative symptoms schizophrenia ($\chi 2$= 3.042 (df = 2), p= 0.218, I^2= 95%).
The maximum reduction of negative symptoms (ED95%) was reached for a total Coulomb of 35 (95CI%: 32-37), N=5, n=142; mean duration of 1.8 weeks.
eFigure 4.b Dose-response curve of LDLPFC tDCS effect on positive symptoms schizophrenia ($\chi 2$= 2.563 (df = 2), p= 0.277, I^2> 95%).

© 2024 Sabé M et al. *JAMA Network Open.*

The maximum reduction of positive symptoms (ED95%) was reached for a total Coloumb of 72 (95CI%: 52-92), N=5, n=142; mean duration of 1.8 weeks.

© 2024 Sabé M et al. *JAMA Network Open.*

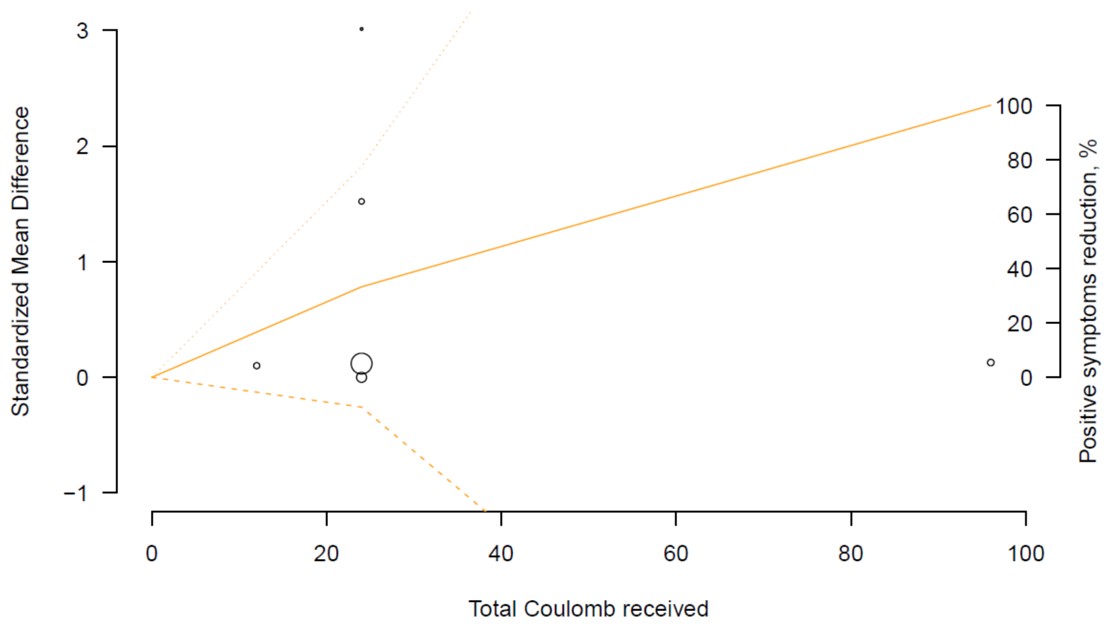

eFigure 5. Dose-Response Curve of LDLPFC tDCS Treatment-Resistant Hallucinations in Patients With Schizophrenia (χ2= 2.179 (df = 2), p= 0.336, I^2> 95%). The maximum reduction of treatment-resistant hallucinations (ED95%) was reached for a total Coulomb of 75 (95CI%: 72-78), N=6, n=242; mean duration of 1.66 weeks.

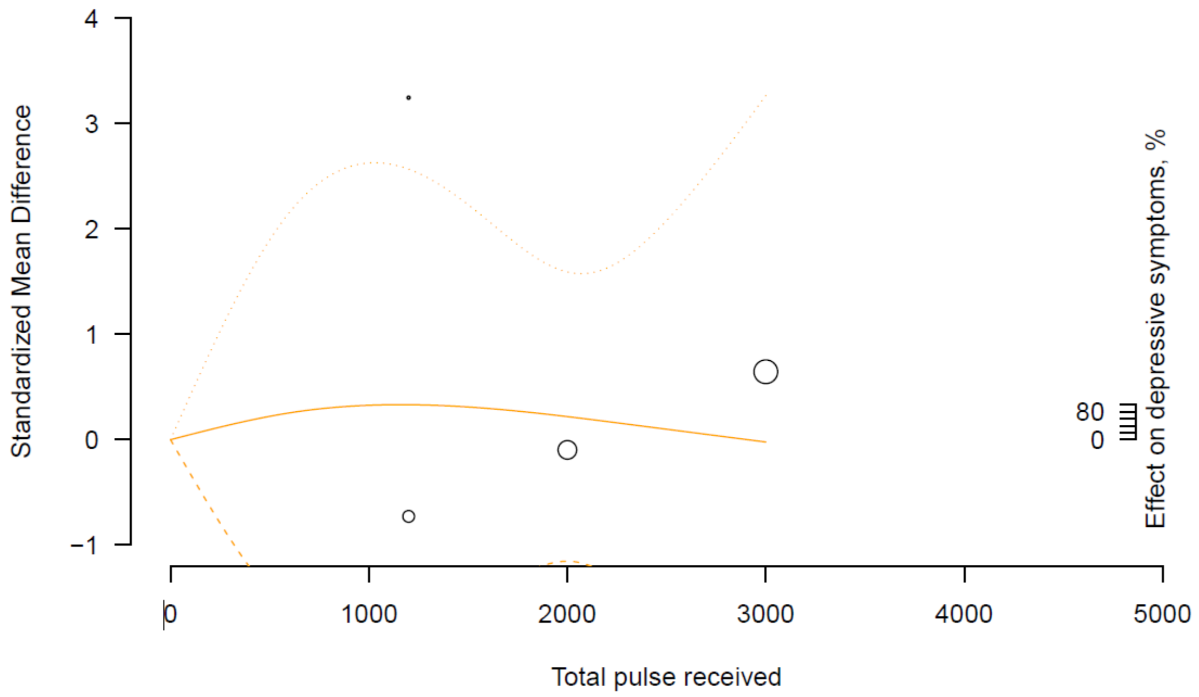

eFigure 6. Dose-Response Curve of LF-RDLPFC TMS for Patients Treatment-Resistant Depression (χ2= 0.113 (df = 2), p=0.945, I^2= 95%).
The maximum effect on depressive symptoms (ED95%) was reached for a total pulse of 889.7(95CI%: 782-994), N=4, n=102; mean duration of 2.5 weeks.

© 2024 Sabé M et al. *JAMA Network Open.*

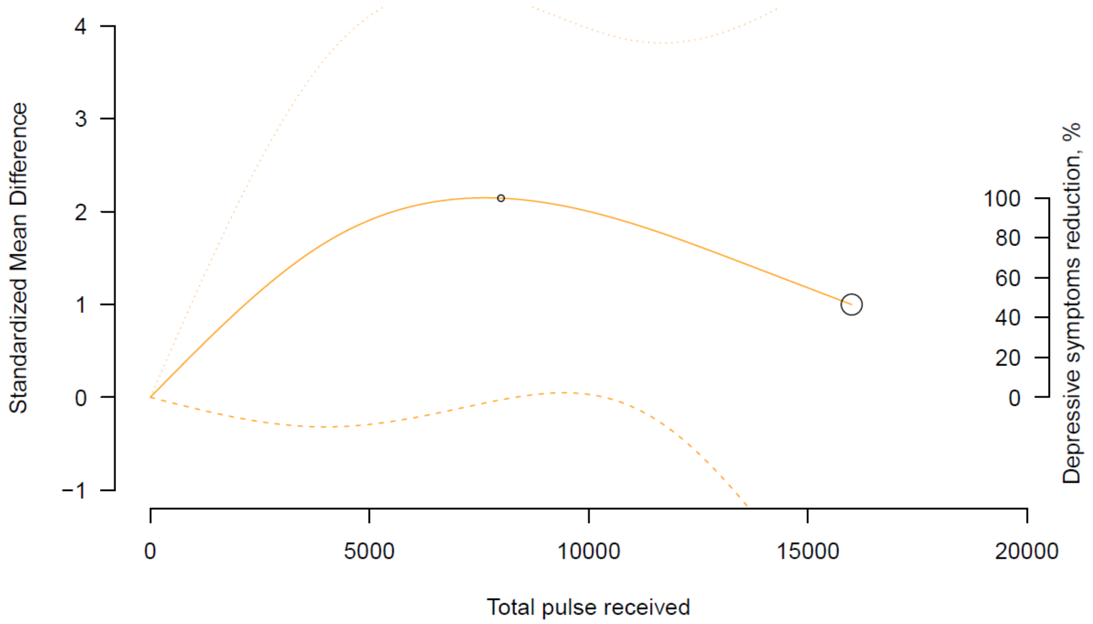

eFigure 7. Dose-Response Curve of HF-LDLPFC rTMS for Patients With Bipolar Depression. Although the dose-response association was not significant (p=0.133), the maximum reduction of depressive symptoms (ED95%) was reached for a total pulse of 5845(95CI%: 210-300), N=2, n=42; mean duration of 2.2 weeks.

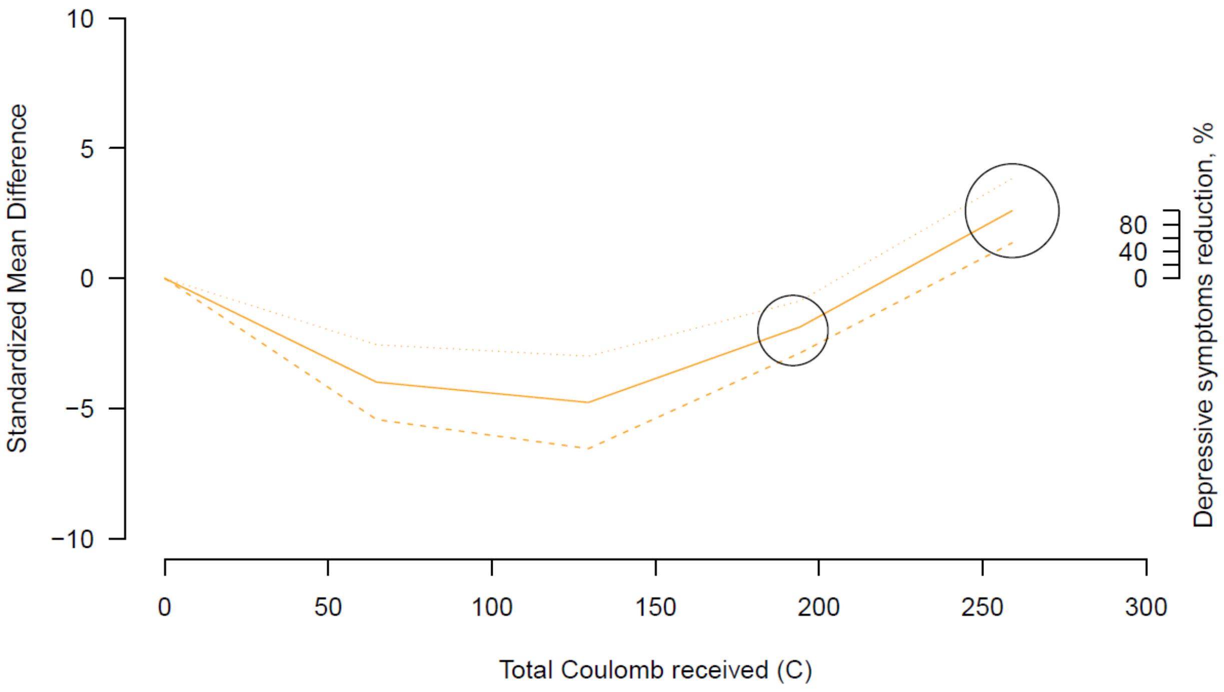

eFigure 8. Dose-Response Curve of LDLPFC tDCS for Patients With Bipolar Depression. Although the dose-response association was not significant (p=0.568), the maximum reduction of depressive symptoms (ED95%) was reached for a total Coulomb of 255(95CI%: 210-300), N=2, n=95; mean duration of 5 weeks.

© 2024 Sabé M et al. *JAMA Network Open.*

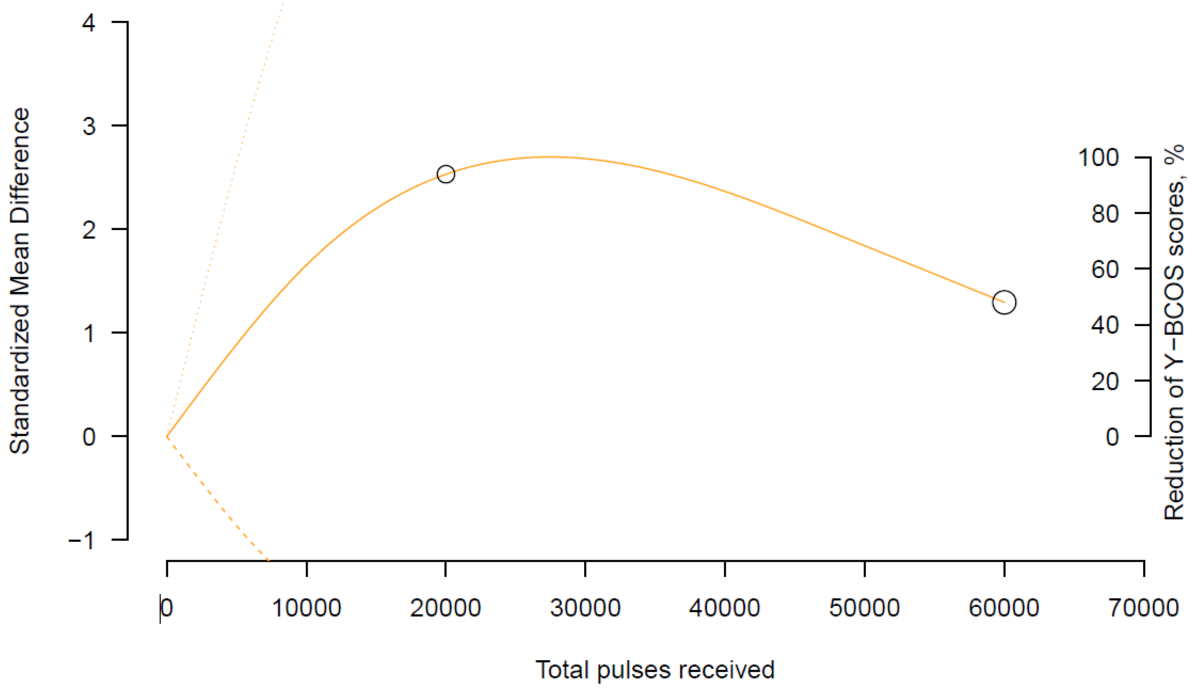

eFigure 9. Dose-Response Curve of HF-RDLPFC rTMS for Patients With OCD (χ2= 1.275(df = 2), p= 0.528, I^2> 95%).

The maximum improvement of bipolar depression symptoms (ED95%) was reached for a total pulse of 20715(95CI%: 17825-23495), N=2, n=57; mean duration of 8 weeks.

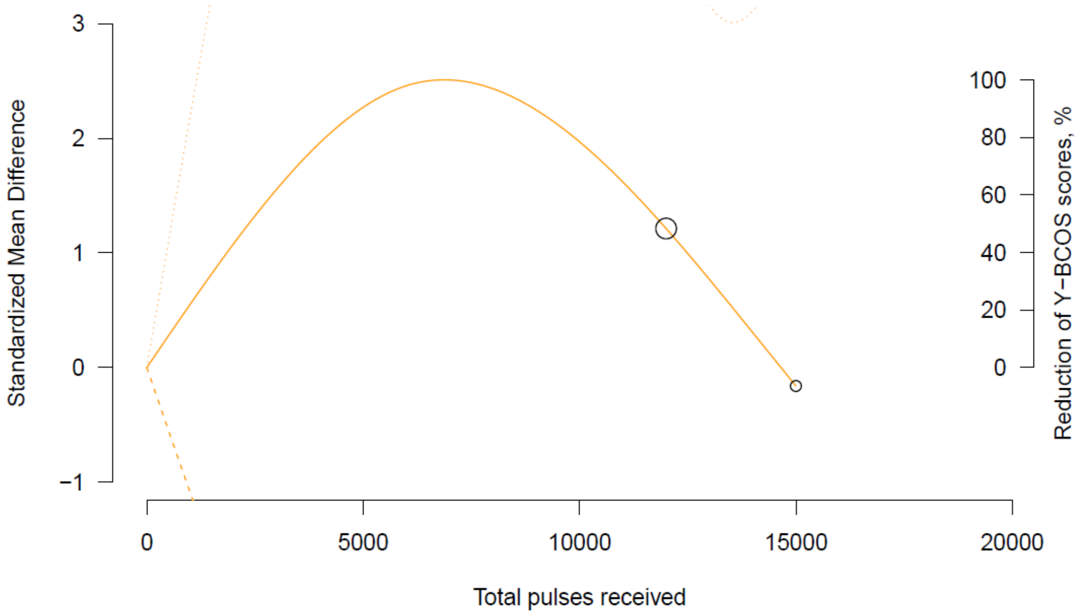

eFigure 10. Dose-Response Curve of HF-LDLPFC rTMS for Patients With OCD (χ2= 0.556(df = 2), p=0.72, I^2= 95%). The maximum improvement of the Y-BCOS scores (ED95%) was reached for a total pulse of 5516(95CI%: 2652-3625), N=2, n=58; mean duration of 2.5 weeks.

© 2024 Sabé M et al. *JAMA Network Open.*

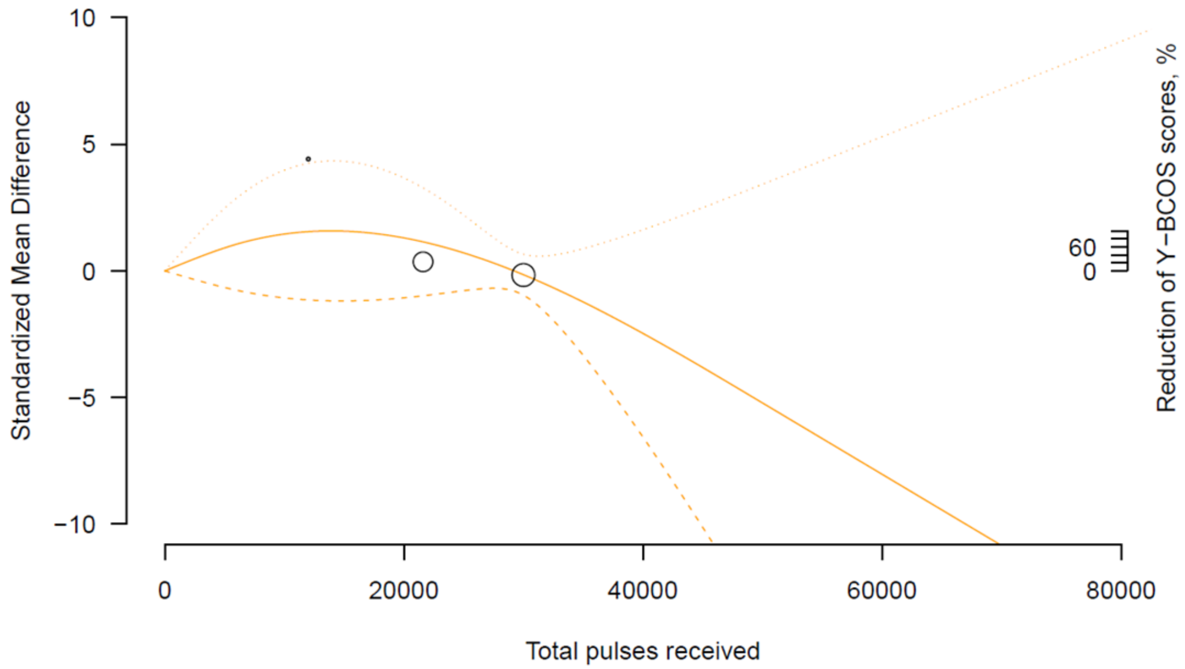

eFigure 11. Dose-Response Curve of LF-SMA rTMS for Patients With OCD (χ2= 1.275(df = 2), p=0.497, I^2> 95%). The maximum reduction of Y-BCOS (ED95%) was reached for a total pulse of 20715 (95CI%: 17825-23495), N=4, n=116; mean duration of 3.25 weeks.

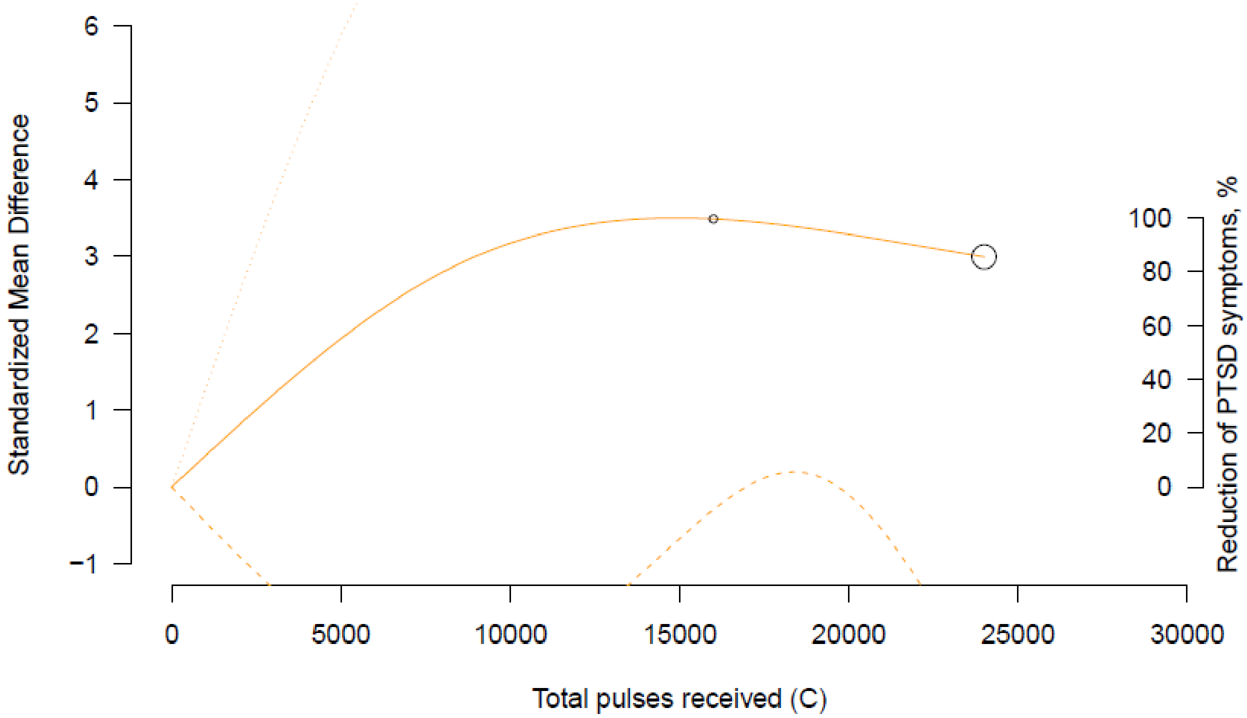

eFigure 12. Dose-Response Curve of HF-RDLPFC rTMS for Patients With PTSD (χ2= 4.322(df = 2), p=0.115, I^2=95%). The maximum reduction of DDQ scores (ED95%) was reached for a total pulse of 11234(95CI%: 10154-12546), N=2, n=53; mean duration of 3 weeks.

© 2024 Sabé M et al. *JAMA Network Open.*

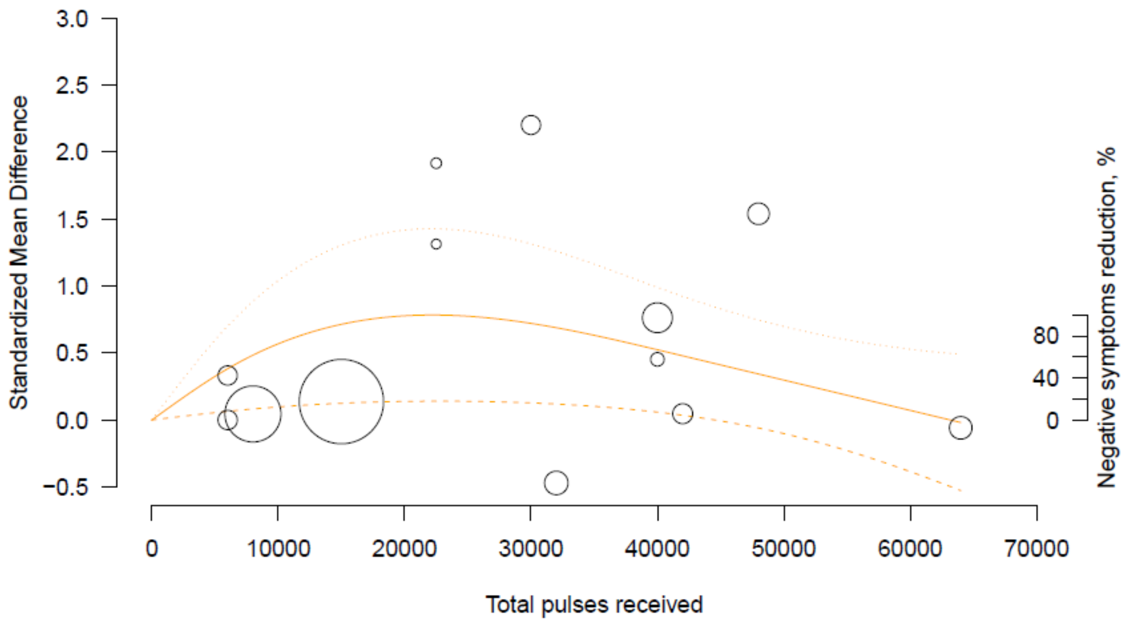

eFigure 13. Sensitivity Analysis Excluding High-Risk Bias Studies Focusing on HF-LDLPFC TMS total Pulses Received for negative symptoms score of patients with schizophrenia (N=13; n=684). A significant dose-response association with a bell-shape curve was obtained (χ2=5.713; p=0.05) in presence of a considerable heterogeneity (I^2=95%), suggesting that higher frequencies were not associated with improved reduction of negative symptoms scores. The ED95% was 16720(11523; 22652).

eAppendix 3. Overall summary of sensitivity analyses (eFigure 13 to eFigure 18)

For HF-LDLPFC TMS delivered to patients with schizophrenia, 4 studies with high risk of bias were excluded. Nevertheless, the association remained significant with a similar bell-shape curve (eFigure 13). The ED95% was now lower at 16720 total pulses.

For HF-LDLPFC TMS for patients with treatment-resistant depression, 5 studies with high risk of bias were excluded. Results were unchanged. The ED95% was similar - at 13314 total pulses (eFigure 14). For LF-RDLPFC and BLDLPFC TMS for patients with treatment resistant depression, no high bias studies were found, as for LF-RDLPFC for patients with depression.

Regarding HF-LDLPFC TMS for patients with depression, the exclusion of 3 studies with risk of bias did not change results (eFigure 15).

For LDLPFC tDCS for patient with SUD, one study with a high risk of bias was excluded. The results were even more significant (p<0.001) with an ED95% at 18.4 C (I^2=60%)(eFigure 16). For HF-RDLPFC TMS

© 2024 Sabé M et al. *JAMA Network Open.*

for PTSD patients no risk of bias was present among retained studies. Moreover, LDLPFC tDCS studies for patients with depression, results were not impacted (eFigure 17).

Finally, for RDLPFC TMS for SUD, we excluded one high-risk studies, however results were unchanged (eFigure 18).

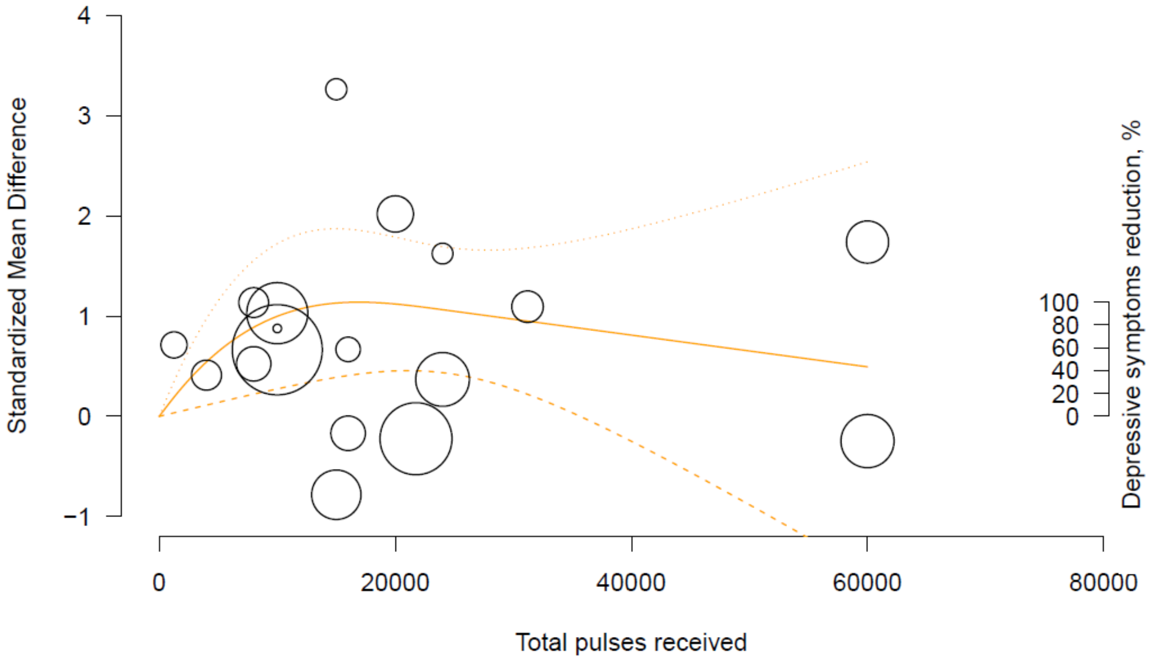

eFigure 14. Sensitivity Analysis Excluding High-Risk Bias Studies on HF-LDLPFC TMS total pulses received for patients with treatment-resistant depression (N=19; n=513). A significant dose-response association with a bell-shape curve was obtained ($\chi 2$=11.226; p=0.003) in presence of a considerable heterogeneity (I^2=95%), suggesting that higher frequencies were not associated with further improved reduction of depression scores. The ED95% was 12314(9553;12734).

© 2024 Sabé M et al. *JAMA Network Open.*

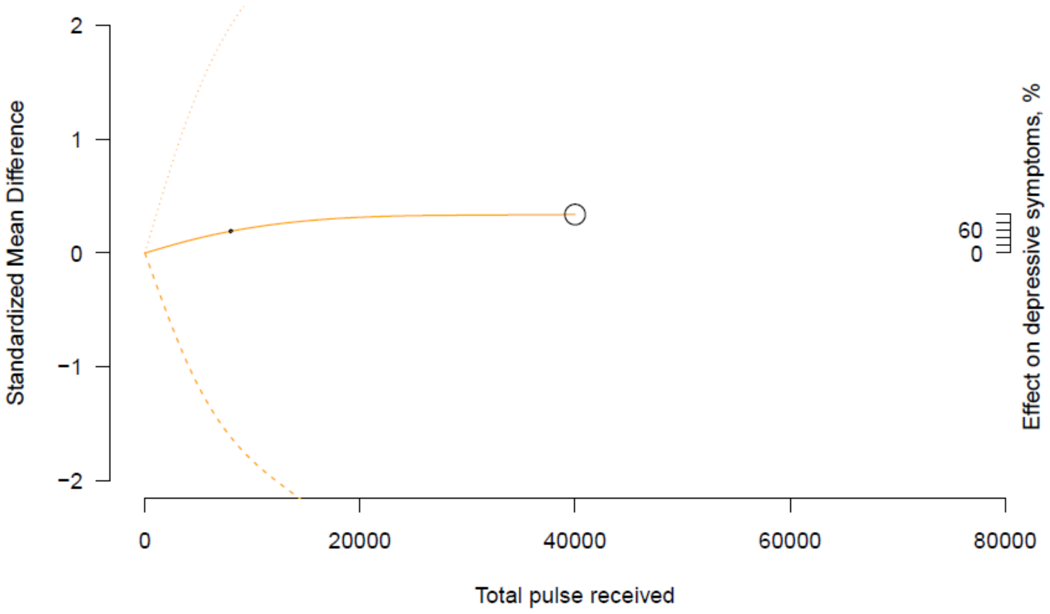

eFigure 15. Sensitivity Analysis Excluding High-Risk Bias Studies on HF-LDLPFC TMS Total Pulses Received for Patients With Major Depressive Disorder (N=2; n=139). No significant dose-association was obtained ($\chi 2$= 0.087; p=0.957). The visual inspection of the curve reveals a flat curve. These results are found in presence of a considerable heterogeneity (I^2=95%), with an important uncertainty. The ED95% was 21948(8452; 27232).

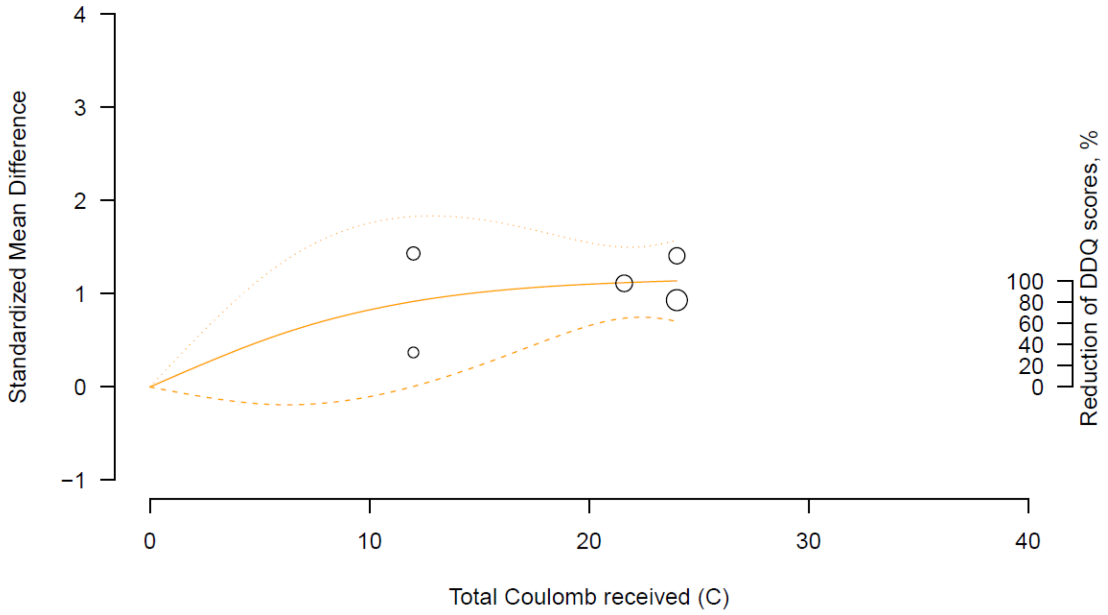

eFigure 16. Sensitivity Analysis Excluding High-Risk Bias Studies on LDLPFC tDCS for Patients With SUD (N=5; n=174). A significant dose-response association with a curve that plateaued was obtained ($\chi 2$= 34.20; p<0.001) in presence of a moderate heterogeneity (I^2=60%), suggesting that higher frequencies were not associated with further improved reduction of depression scores. The ED95% was 18.42(15.9; 22.1).

© 2024 Sabé M et al. *JAMA Network Open.*

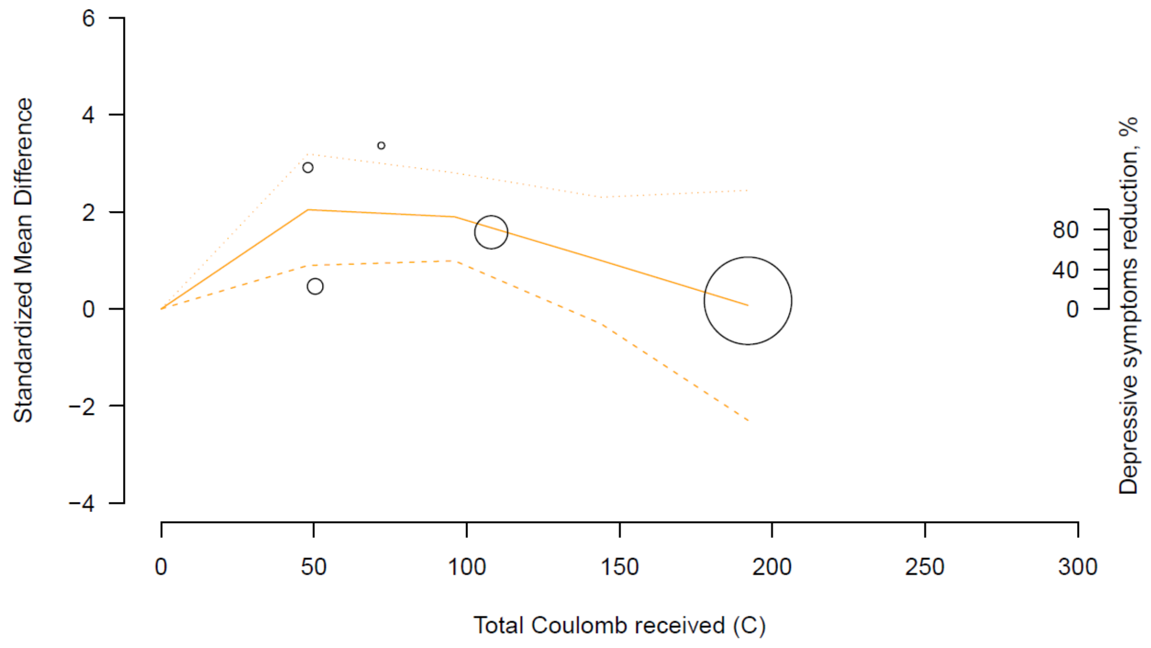

eFigure 17. Sensitivity Analysis on LDLPFC tDCS for Patients With Resistant Depression. A significant dose-response association with a bell-shape curve was obtained (p<0.001) in presence of a considerable heterogeneity (I^2=95%), suggesting that higher frequencies were not associated with improved reduction of depressive symptoms scores. The ED95% was 49(41.2; 57.6).

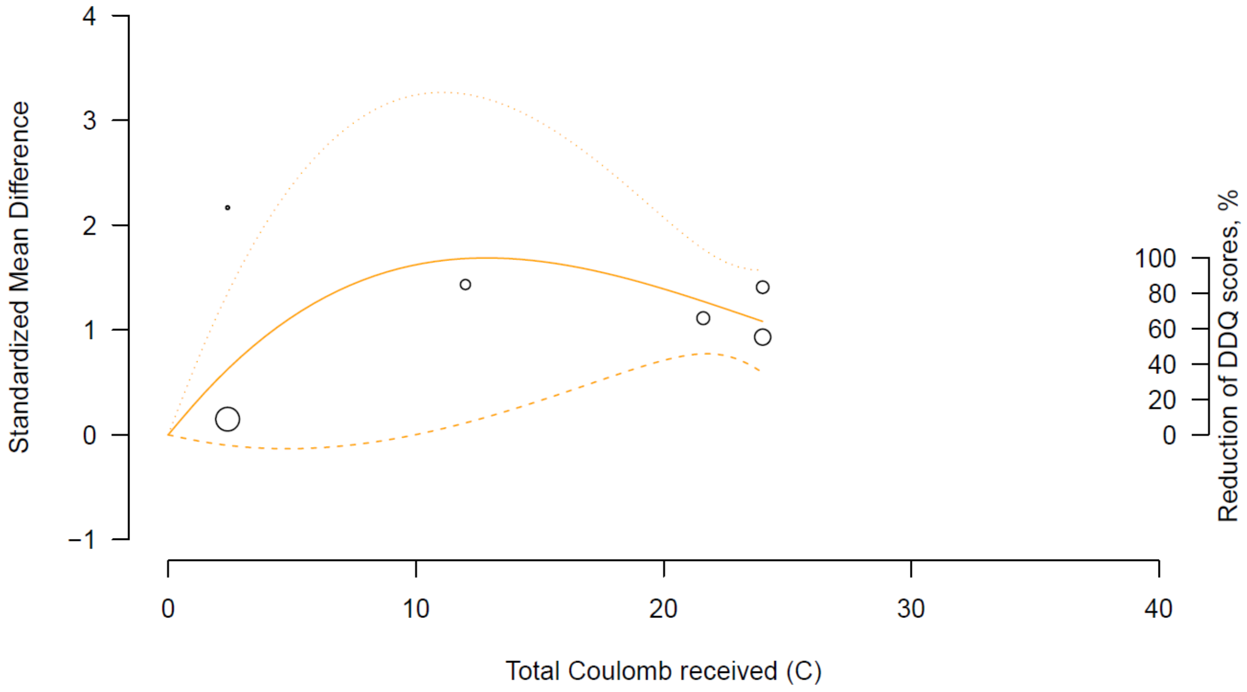

eFigure 18. Sensitivity Analysis for Patients Focusing on RDLPFC tDCS Received for Patients With SUD (MUD and CUD). A significant dose-response with bell-shape curve was obtained (p<0.001) in presence of a considerable heterogeneity (I^2=60%). The ED95% was 9.80(8.33; 23.51).

eAppendix 4. Additional analyses regarding the frequency used in TMS studies

© 2024 Sabé M et al. *JAMA Network Open.*

We conducted an additional analysis regarding the frequency used rather than the dose of stimulation. HF-LDLPFC TMS for patients with schizophrenia presented a bell-shape curve with a significant dose-response association (p=0.01) in presence of considerable heterogeneity (I^2=95%) suggesting that frequencies superior to 11Hz were less effective for negative symptoms reduction (eFigure 19).

HF-LDLPFC for resistant-depression patients showed a curve that plateaued with an ED95% at 9.8 in presence of a considerable heterogeneity (I^2=95%), suggesting that higher frequencies were not associated to further decrease of depressive symptoms (eFigure 20). Since only 10 Hz frequencies were used for HF-LDLPFC TMS for patients with depression, no sensitivity analysis was possible.

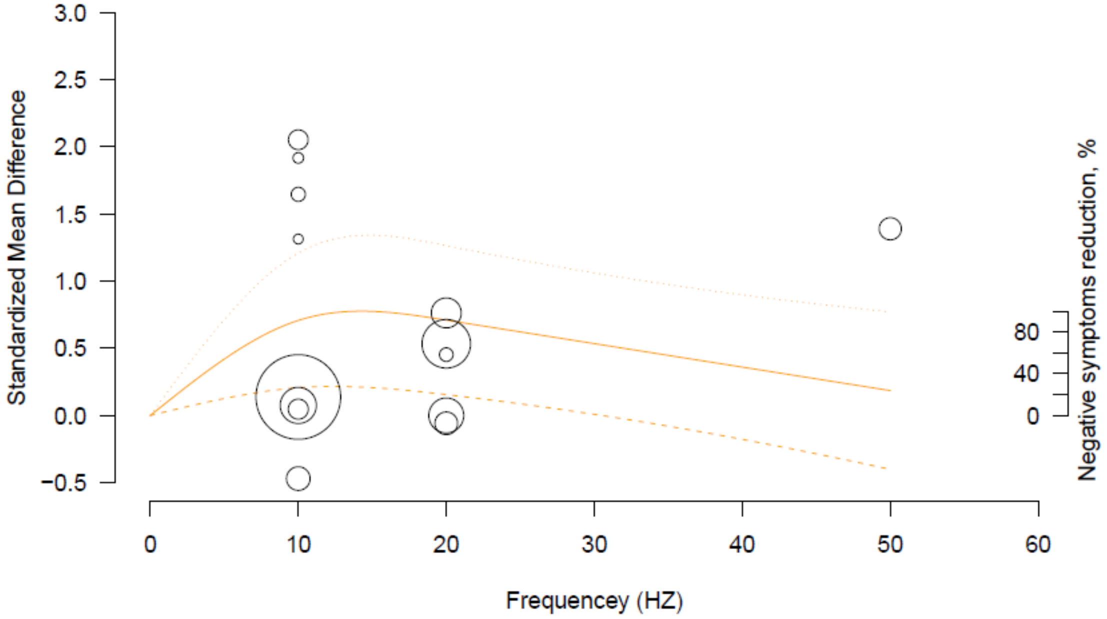

eFigure 19. Sensitivity Analysis on HF-LDLPFC TMS Frequencies for Patient With Schizophrenia. A significant dose-response association with a bell-shape curve was obtained (p=0.01) in presence of a considerable heterogeneity (I^2=95%), suggesting that higher frequencies were not associated with improved reduction of negative symptoms scores. The ED95% was 11.1 (9.6; 14.4).

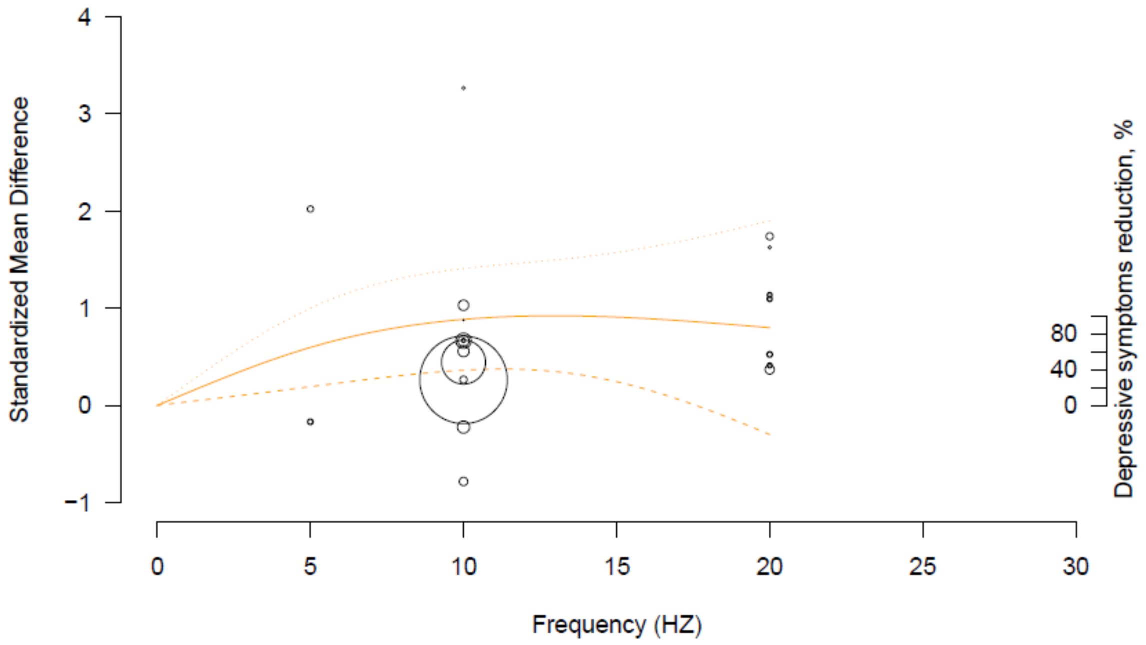

eFigure 20. Sensitivity Analysis on HF-LDLPFC TMS Frequencies Received for Patients With Resistant Depression. A significant dose-response association with a bell-shape curve was obtained ($p<0.01$) in presence of a considerable heterogeneity (I^2= 95%), suggesting that higher frequencies were not associated with improved reduction of depressive symptoms scores. The ED95% was 9.80(6.21; 19.18).

© 2024 Sabé M et al. *JAMA Network Open.*

eTable 1. Study Characteristics of Included Randomized Controlled Trials

Study	Included study arms	Total study arms	Arm	N	Age (SD)	Coil type/ electrode sponge size	Cortical target	Method of locating target	TR	Hz/mA (% MT)	Treatment strategy	Number of sessions (weeks)	Included scales/ tasks	Source of outcome data
**Depression**														
Anderson, et al. (2007)	2	2	rTMS	13	48 (8)	Figure-of-eight coil	LDLPFC	5cm rule	Mixed	10 Hz (110)	Mixed	12 (4-6w)	MADRS	Previous analysis[5]
			Sham	16	46 (12)									
Avery, et al. (1999)	2	2	rTMS	4	44.3 (10.1)	NI	LDLPFC	5cm rule	Yes	10 Hz (80)	Mixed	10 (3w)	HDRS-21	Previous analysis[5]
			Sham	2	45 (7.1)									
Asl et al. (2022)	2	2	rTMS	14	36.25 (8.7)	Figure-of-eight coil	LDLPFC	5cm rule	Yes	20 Hz (85)	Augmentation	10 (2w)	BDI	Original article
			Sham	14	34.5 (9.5)									
Baeken, et al. (2013)	2	2	rTMS	10	51.8 (12.1)	Figure-of-eight coil	LDLPFC	3D-MRI	Yes	20 Hz (110)	Monotherapy	20 (1w)	HDRS-17	Previous analysis[5]
			Sham	11	47.3 (13.7)									
Bakim, et al. (2012)	3[a]	3	rTMS	23	40.8 (9)	Figure-of-eight coil	LDLPFC	5cm rule	Yes	20 Hz (80,110)	Augmentation	30 (6w)	HDRS-17	Previous analysis[5]
			Sham	12	44.4 (10.2)									
Berman, et al. (2000)	2	2	rTMS	10	45.2 (9.5)	Figure-of-eight coil	LDLPFC	5cm rule	Yes	20 Hz (80)	Monotherapy	10 (2w)	MADRS	Previous analysis[5]
			Sham	10	39.4 (10.8)									
Blumberger, et al. (2012)A	3	3	rTMS	28	58 (12.5)	70mm Figure-of-eight coil	BL	5cm rule	Yes	1 Hz R, 10 Hz LDLPFC (100,120)	Mixed	15 (3w)	HDRS-17	Previous analysis[5]
			rTMS	24	48.9 (13.4)		LDLPFC			10 Hz (100, 120)		15 (3w)		
			Sham	22	45.8 (13.4)									
Blumberger et al. (2012) B	2	2	rTMS	17	36.6 (8.2)	70mm Figure-of-eight coil	LDLPFC	5cm rule	Yes	6Hz	Mixed	20	PSYRATS	
			rTMS	17	43.8 (11.7)									
			Sham	17	40.8 (12.1)									

© 2024 Sabé M et al. *JAMA Network Open.*

Study	Included study arms	Total study arms	Arm	N	Age (SD)	Coil type/ electrode sponge size	Cortical target	Method of locating target	TR	Hz/mA (% MT)	Treatment strategy	Number of sessions (weeks)	Included scales/ tasks	Source of outcome data
**Depression**														
Bortolomasi, et al. (2007)	2	2	rTMS	12	55.6 (15.4)	90mm circular coil	LDLPFC	5cm rule	Yes	20 Hz (90)	Mixed	5 (1w)	HDRS-24	Previous analysis[5]
			Sham	7										
Boutros, et al. (2002)	2	2	rTMS	12	49.5 (8)	70mm Figure-of-eight coil	LDLPFC	5cm rule	Yes	20 Hz (80)	Mixed	10 (2w)	HDRS-25	Previous analysis[5]
			Sham	10	52 (7)									
Chen, et al. (2013)	2	2	rTMS	10	44.1 (4.4)	Figure-of-eight coil	LDLPFC	5cm rule	Yes	20 Hz (90)	Augmentation	10 (4w)	HDRS-21	Previous analysis[5]
			Sham	11	47.3 (3.5)									
Eschweiler et al. (2000)	3	3	rTMS	5	57 (8)	Figure-of-eight coil	LDLPFC	5cm rule		10 Hz (90)		20(4w)		
			Sham	5	57 (8)									
Fitzgerald, et al. (2003)	3	3	rTMS	20	45.6 (11.5)	70mm Figure-of-eight coil	RDLPFC	5cm rule	Yes	1 Hz (100)	Mixed	10 (2w)	MADRS	Previous analysis[5]
			rTMS	20	42.2 (9.8)		LDLPFC			10 Hz (100)		10 (2w)		
			Sham	20	49.2 (14.2)									
Fitzgerald, et al. (2012)	3	3	rTMS	24	43.4 (12.7)	70mm Figure-of-eight coil	LDLPFC	MRI	Yes	10 Hz (120)	Mixed	15 (3w)	MADRS	Previous analysis[5]
			rTMS	22	40.5 (15.5)		BL			10 Hz (120)		15 (3w)		
			Sham	20	44.9 (15.7)									
George, et al. (1997)	2	2	rTMS	7	42.4 (15.5)	Figure-of-eight coil	LDLPFC	5cm rule	No	20 Hz (80%)	Mixed	10 (2w)	HDRS-21	Previous analysis[5]
			Sham	5	41 (8.3)									
George, et al. (2000)	3[2]	3	rTMS	22	42.4 (10.8)	Figure-of-eight coil	LDLPFC	5cm rule	Mixed	20 Hz (100%)	Monotherapy	10 (2w)	HDRS-21	Previous analysis[5]
			Sham	10	48.5 (8)									
George, et al. (2010)	2	2	rTMS	92	47.7 (10.6)	Figure-of-eight coil	LDLPFC	5cm rule	Yes	10 Hz (120%)	Monotherapy	15 (3w)	HDRS	Previous analysis[5]
			Sham	98	46.5 (12.3)									
Holtzheimer, et al. (2004)	2	2	rTMS	7	40.4 (8.5)	Figure-of-eight coil	LDLPFC	5cm rule	Yes	10 Hz (110%)	Monotherapy	10 (2w)	HDRS-17	Previous analysis[5]
			Sham	8	45.4 (4.9)									

© 2024 Sabé M et al. *JAMA Network Open.*

Study	Included study arms	Total study arms	Arm	N	Age (SD)	Coil type/ electrode sponge size	Cortical target	Method of locating target	TR	Hz/mA (% MT)	Treatment strategy	Number of sessions (weeks)	Included scales/ tasks	Source of outcome data
**Depression**														
Hoppner, et al. (2003)	3	3	rTMS	10	60.4 (7.1)	Figure-of-eight coil	LDLPFC	5cm rule	NA	20 Hz (90)	Augmentation	10 (2w)	HDRS-21	Previous analysis[5]
			rTMS	10	52 (11.7)		RDLPFC			1 Hz (110)		10 (2w)		
			Sham	10	56.4 (13.2)									
Januel, et al. (2006)	2	2	rTMS	11	38.6 (11.2)	Figure-of - eight coil	RDLPFC	5cm rule	No	1 Hz (90)	Monotherapy	16 (4w)	HDRS-17	Previous analysis[5]
			Sham	16	37.2 (11.7)									
Kauffmann, et al. (2004)	2	2	rTMS	7	51.7 (17.2)	90mm circular coil	RDLPFC	5cm rule	Yes	1 Hz (110)	Augmentation	10 (2w)	HDRS-21	Previous analysis[5]
			Sham	5										
Klein, et al. (1999)A	2	2	rTMS	36	60.5 (15.1)	90mm circular coil	RDLPFC	6cm rule	No	1 Hz (110)	Mixed	10 (2w)	HDRS-17	Previous analysis[5]
			Sham	34	58.9 (18.3)									
Kreuzer, et al. (2015)	2	2	rTMS	15	46.1 (9.5)	Figure-of-eight coil	LDLPFC	6cm rule	Mixed	10 Hz (110)	Mixed	15 (3w)	HDRS-21	Previous analysis[5]
			Sham	15	43.8 (10.5)									
Loo, et al. (2010)	2	2	tDCS	20	49 (10)	$35cm^2$	Anode: LDLPFC, Cathode: F8[3]	10-20 EEG system	Mixed	1mA	Mixed	10 (2w)	HDRS-17, TMT-A, TMT-B, digit span	Original article
			Sham	20	45.6 (12.5)									
Loo, et al. (2012)	2	2	tDCS	33	47.8 (12.5)	$35cm^2$	Anode: LDLPFC, Cathode: F8[3]	10-20 EEG system	Yes	2mA	Mixed	15 (3w)	MADRS, stroop interference test, digit span,	Original article
			Sham	31	48.6 (12.6)									
Loo, et al. (2018)	2	2	tDCS	66	18-81	$35cm^2$	Anode: LDLPFC, Cathode: F8[3]	10-20 EEG system	Mixed	2.5mA	Mixed	20 (4w)	MADRS	Previous analysis[5]
			Sham	64										

© 2024 Sabé M et al. *JAMA Network Open.*

Study	Included study arms	Total study arms	Arm	N	Age (SD)	Coil type/ electrode sponge size	Cortical target	Method of locating target	TR	Hz/mA (% MT)	Treatment strategy	Number of sessions (weeks)	Included scales/ tasks	Source of outcome data
**Depression**														
McDonald, et al. (2006)	3[2]	3	rTMS	50	NR	Figure-of-eight coil	BL	5cm rule	Yes	10 Hz L, 1 Hz RDLPFC (110)	Monotherapy	10 (2w)	HDRS-21, RBANS attention	Original article
			Sham	12										
Moirand et al. 2022	2	2	tDCS	21	51.5 (9.7)	35cm2	Anode:L DLPFC; Cathode: rDLPFC	10-20 EEG system	Yes	2 mA	Augmentation	10 (2w)	MADRS	Original article
			Sham	18	48.1 (9.3)									
Mogg, et al. (2008)	2	2	rTMS	29	55 (18)	Figure-of-eight coil	LDLPFC	5cm rule	Yes	10 Hz (110)	Mixed	10 (2w)	HDRS-17	Previous analysis[5]
			Sham	30	52 (15.5)									
O'Reardon, et al. (2007)	2	2	rTMS	165	47.9 (11)	NI	LDLPFC	5cm rule	Yes	10 Hz (120)	Monotherapy	20 (4-6w)	HDRS-17	Previous analysis[5]
			Sham	160	48.7 (10.6)									
Padberg, et al. (1999)	3	3	rTMS	6	46.7 (14.7)	Figure-of-eight coil	LDLPFC	5cm rule	Yes	0.3 Hz (90)	Mixed	5 (1w)	HDRS-21	Original article
			rTMS	6	63.5 (15.8)		LDLPFC			10 Hz (90)		5 (1w)		
			Sham	6	43.3 (11.6)									
Padberg, et al. (2002)	3[1]	3	rTMS	20	61.2 (13.8)	70mm Figure-of-eight coil	LDLPFC	5cm rule	Yes	10 Hz (100)	Augmentation	10 (2w)	HDRS-21	Previous analysis[5]
			Sham	10	52.7 (18)									
Paillère-Martinot, et al. (2010)	2	3	rTMS	19	48.2 (7.8)	Figure-of-eight coil	LDLPFC	MRI	Yes	10 Hz (90)	Augmentation	10 (2w)	HDRS-21	Previous analysis[5]
			Sham	14	46.6 (10.3)									
Prasser, et al. (2015)	3	3	rTMS	18	50.4 (9.9)	Figure-of-eight coil	BL	6cm rule	Mixed	1 Hz R, 10 Hz L (110, 100)	Augmentation	15 (3w)	HDRS-21	Contacted authors
			TBS	20	48.2 (10.9)		BL			TBS (80)		15 (3w)		
			Sham	18	42.6 (12.4)									
Salehinejad, et al. (2015)	2	2	tDCS	15	28.7 (5.9)	35cm^2 electrodes	Anode: LDLPFC, Cathode: RDLPFC	10-20 EEG system	Yes	2mA	Monotherapy	10 (2w)	HDRS-24	Previous analysis[5]
			Sham	15	27.9 (5.8)									

© 2024 Sabé M et al. *JAMA Network Open.*

Study	Included study arms	Total study arms	Arm	N	Age (SD)	Coil type/ electrode sponge size	Cortical target	Method of locating target	TR	Hz/mA (% MT)	Treatment strategy	Number of sessions (weeks)	Included scales/ tasks	Source of outcome data
**Depression**														
Salehinejad, et al. (2017)	2	2	tDCS	12	26.8 (7.1)	$35cm^2$	Anode: LDLPFC, Cathode: RDLPFC	10-20 EEG system	Yes	2mA	Monotherapy	10 (2w)	HDRS-24	Previous analysis[5]
			Sham	12	25.5 (4.6)									
Sampaio Junior, et al. (2018)	2	2	tDCS	30	46.2 (11.8)	$25cm^2$	Anode: LDLPFC, Cathode: RDLPFC	10-20 EEG system	Mixed	2mA	Augmentation	12 (6w)	HDRS-17	Previous analysis[5]
			Sham	29	45.7 (10.3)									
Speer, et al. (2014)	3	3	rTMS	8	39.6 (9)	Figure-of-eight coil	LDLPFC	5cm rule	Yes	1 Hz (110)	Monotherapy	15 (3w)	HDRS-28	Previous analysis[5]
			rTMS	8	41.3 (14.5)		LDLPFC			20 Hz (110)		15 (3w)		
			Sham	8	44.9 (9.1)									
Su, et al. (2005)	3[1]	3	rTMS	22	43.4 (11)	Figure-of-eight coil	LDLPFC	5cm rule	Yes	20 Hz (100)	Augmentation	10 (2w)	HDRS-21	Previous analysis[5]
			Sham	11	42.6 (11)									
Taylor, et al. (2018)	2	2	rTMS	20	46.9 (10.7)		LDLPFC	MRI	Yes	10 Hz (120)	Mixed	20 (4w)	HDRS-17	Previous analysis[5]
			Sham	20	44.1 (11.1)									
Triggs, et al. (2010)	4[1]	4	rTMS	16	48.5 (10.8)	70mm Figure-of-eight coil	RDLPFC	5cm rule	Yes	5 Hz (100)	Augmentation	10 (2w)	HDRS-24	Previous analysis[5]
			rTMS	18	46.7 (15.3)		LDLPFC			5 Hz (100)		10 (2w)		
			Sham	14	44.3 (17.4)									
Tong et al. 2021	2	2	rTMS	55	48.65 (14.04)	70mm Figure-of-eight coil	LDLPFC	5cm rule	No	10 Hz (120)	Augmentation	20(4 w)		Original article
			Sham	53	47.7 (13.4)									
Tsai et al. 2021	3	3	rTMS	20	49.1	70mm Figure-of-eight coil	LDLPFC	MRI	Yes	10 Hz	Augementation	10(2w)		Original article
			iTBS	19	48.74	70mm Figure-of-eight coil	LDLPFC	MRI	Yes	5 Hz	Augementation	10(2w)		
			Sham	22	48.5									
van Eijndhoven, et al. (2020)	2	2	rTMS	15	47.3 (11.5)	70mm Figure-of-eight coil	LDLPFC	10-20 EEG system	Yes	10 Hz (110)	Mixed	20 (4w)	HDRS-17	Original article
			Sham	16	49.7 (11)									

© 2024 Sabé M et al. *JAMA Network Open.*

Study	Included study arms	Total study arms	Arm	N	Age (SD)	Coil type/ electrode sponge size	Cortical target	Method of locating target	TR	Hz/mA (% MT)	Treatment strategy	Number of sessions (weeks)	Included scales/ tasks	Source of outcome data
**Depression**														
Zheng, et al. (2010)	2	2	rTMS	19	26.9 (6.2)	Figure-of-eight coil	LDLPFC	5cm rule	Yes	15 Hz (110)	Augmentation	20 (4w)	HDRS-17	Previous analysis[5]
			Sham	15	26.7 (4.3)									

Study	Included study arms	Total study arms	Arm	N	Age (SD)	Coil type/ electrode sponge size	Cortical target	Method of locating target	TR	Hz/mA (% MT)	Treatment strategy	Number of sessions (weeks)	Included scales/ tasks	Source of outcome data
**OCD**														
Alonso, et al. (2001)	2	2	rTMS	10	39.2 (13)	70mm circular coil	RDLPFC	MRI	Yes	1 Hz (110)	Mixed	18 (6w)	Y-BOCS	Previous analysis[6]
			Sham	8	30.3 (9.5)									
Arumugham, et al. (2018)	2	2	rTMS	19	27.74 (7.88)	Figure-of-eight coil	SMA	10-20 EEG system	Yes	1 Hz (100)	Augmentation	18 (3w)	Y-BOCS	Previous analysis[6]
			Sham	17	30.7 (10.43)									
Badawy, et al. (2010)	2	2	rTMS	20	27.7 (7.83)	NI	LDLPFC	NI	Yes	20 Hz (NR)	Monotherapy	(3w)	Y-BOCS	Previous analysis[6]
			Sham	20	28.9 (5.7)									
Elbeh, et al. (2016)	3	3	rTMS	15	28.9 (3.9)	70mm Figure-of-eight	RDLPFC	5cm rule	NI	10 Hz (100%)	Mixed	10 (2w)	Y-BOCS	Previous analysis[6]
			rTMS	15	26.8 (5.2)		RDLPFC			1 Hz (100%)		10 (2w)		
			Sham	15	25.5 (4)									
Gomes, et al. (2012)	2	2	rTMS	12	35.5 (7.5)	70mm Figure-of-eight coil	SMA	10-20 EEG system	Yes	1 Hz (100)	Augmentation	10 (2w)	Y-BOCS	Previous analysis[6]
			Sham	10	37.5 (16)									

© 2024 Sabé M et al. *JAMA Network Open.*

Study	Included study arms	Total study arms	Arm	N	Age (SD)	Coil type/ electrode sponge size	Cortical target	Method of locating target	TR	Hz/mA (% MT)	Treatment strategy	Number of sessions (weeks)	Included scales/ tasks	Source of outcome data
**OCD**														
Haghighi, et al. (2015)	2	2	rTMS	10	34.9 (5.91)	70mm Figure-of-eight coil	BL	5cm rule	Yes	20 Hz (100)	Mixed	20 (4w)	Y-BOCS	Previous analysis[6]
			Sham	11	36.6 (3.95)									
Jahangard, et al. (2016)	2	2	rTMS	5	32.4 (8.97)	70mm Figure-of-eight coil	BL	5cm rule	Yes	20 Hz (100)	Augmentation	10 (2w)	Y-BOCS	Previous analysis[6]
			Sham	5	33.8 (5.81)									
Kang, et al. (2009)	2	2	rTMS	10	28.6 (12.66)	70mm Figure-of-eight coil	RDLPFC	5cm rule, 10-20 EEG system	Yes	1 Hz (110)	Mixed	10 (2w)	Y-BOCS	Previous analysis[6]
			Sham	10	26.2 (10.52)									
Khedr et al. (2022)	3	3	rTMS	20	36.9(11.5)	70mm Figure-of-eight coil	RDLPFC	5cm rule	Yes	1 Hz (120)	Augmentation	10 (2w)	Y-BOCS	Original article
			rTMS	20	34(10.2)		OFC							
			Sham	20	35.3(9.4)		Sham							
Ma, et al. (2014)	2	2	rTMS	25	27.12 (8.97)	90mm circular coil	BL	10-20 EEG system	Yes	8-12 Hz (80)	Augmentation	10 (2w)	Y-BOCS	Previous analysis[6]
			Sham	21	29.86 (9.42)									
Mansur, et al. (2011)	2	2	rTMS	13	42.1 (11.9)	Figure-of-eight coil	RDLPFC	5cm rule	Yes	10 Hz (110)	Mixed	30 (6w)	Y-BOCS	Previous analysis[6]
			Sham	14	39.3 (13.9)									
Mantovani, et al. (2010)	2	2	rTMS	9	39.7 (8.6)	70mm Figure-of-eight coil	SMA	10-20 EEG system	Yes	1 Hz (100)	Mixed	20 (4w)	Y-BOCS	Previous analysis[6]
			Sham	9	39.4 (10.2)									
Nauczyciel et al. (2014)	2	2	rTMS	11	39	Figure-of-eight coil	OFC	10-20 EEG system	Yes	1 Hz (110)	Augmentation	10 (1w)	Y-BOCS	Original article
			Sham	11	40									
Pelissolo, et al. (2016)	2	2	rTMS	20	39.1 (10.4)	70mm Figure-of-eight	SMA	MRI	Yes	1 Hz (100)	Mixed	20 (4w)	Y-BOCS	Previous analysis[6]
			Sham	16	42.3 (10.6)									
Prasko, et al. (2006)	2	2	rTMS	18	28.9 (7.7)	70mm Figure-of-eight coil	RDLPFC	5cm rule	Yes	1 Hz (110)	Augmentation	10 (2w)	Y-BOCS	Previous analysis[6]

© 2024 Sabé M et al. *JAMA Network Open.*

			Sham	12	33.4 (8.7)									

Study	Included study arms	Total study arms	Arm	N	Age (SD)	Coil type/ electrode sponge size	Cortical target	Method of locating target	TR	Hz/mA (% MT)	Treatment strategy	Number of sessions (weeks)	Included scales/ tasks	Source of outcome data
**PTSD**														
Ruffini et al. (2009)	2	2	rTMS	16	n.a.	70mm Figure-of-eight coil	OFC	5cm rule	Yes	1 Hz (80)	Augmentation	15(3w)	Y-BOCS	Original article
			Sham	7	n.a.									
Sachdev, et al. (2007)	2	2	rTMS	10	29.5 (9.9)	70mm Figure-of-eight coil	LDLPFC	5cm rule	Yes	10 Hz (110)	Mixed	10 (2w)	Y-BOCS	Previous analysis[6]
			Sham	8	35.8 (8.2)									
Shayganfard, et al. (2016)	2	2	rTMS	5	33.8 (9.55)	70mm Figure-of-eight coil	BL	5cm rule	NA	20 Hz (100)	Augmentation	10 (2w)	Y-BOCS	Previous analysis[6]
			Sham	5	33.2 (7.86)									
Ahmadizadeh and Rezaei (2018)	3	3	rTMS	19	52.1 (7.62)	Figure-of-eight coil	BL	5cm rule	No	20 Hz (100)	Mixed	10 (4w)	PCL-M	Previous analysis[7]
			rTMS	19	51.89 (7.93)		RDLPFC			20 Hz (100)		10 (4w)		
			Sham	20	47.5 (5.61)									
Boggio et al. (2010)	3	3	rTMS	7	47.1	Figure-of-eight coil	LDLPFC	5cm rule	No	20 Hz (100)	Mixed	10 (2w)	PCL-M	
			rTMS	6	40.7		RDLPFC			20 Hz (100)		10 (2w)		
			Sham	8	45.9									
Nam et al. (2013)	2	2	rTMS	8	36.29 (8.79)	Figure-of-eight coil	RDLPFC	5cm rule	No	1 Hz (100)	Mixed	15 (3w)	CAPS	
			Sham	9	32.78 (6.89)									
Watts et al. (2012)	2	2	rTMS	10	54 (12.3)	Figure-of-eight coil	RDLPFC	5cm rule	No	1 Hz (100)	Mixed	10 (2w)	**CAPS**	
			Sham	10	57.8 (11.8)									

© 2024 Sabé M et al. *JAMA Network Open.*

Study	Included study arms	Total study arms	Arm	N	Age (SD)	Coil type/ electrode sponge size	Cortical target	Method of locating target	TR	Hz/mA (% MT)	Treatment strategy	Number of sessions (weeks)	Included scales/ tasks	Source of outcome data
**Schizophrenia**														
Bais et al. (2014)	2	2	rTMS	16	37.2(14.9)	70mm figure-of eight coil	LTPJ	EEG reference points	Yes	1 Hz (90)	Augmentation	12(2w)	AHRS	Original article
			Sham	16	37.3(11.6)									
Barr, et al. (2012)	2	2	rTMS	13	40.46 (12.21)	70mm figure-of eight coil	BL	MINIBIRD system based on MRI	NI	20 Hz (90)	Augmentation	20 (4w)	PANSS	Original article
			Sham	14	47.92 (12.78)									
Bose, et al. (2018)	2	2	tDCS	12	31.25 (8.32)	35cm^2	Anode: LDLPFC, Cathode: LTPJ	EEG reference points	Yes	2 mA	Augmentation	10 (1w)	AHRS, SANS, SAPS	Original article
			Sham	14	31.38 (7.56)									
Brunelin et al. (2006)	2	2	rTMS	14	34.9(8)	70mm figure-of eight coil	LTPJ	EEG reference points	Yes	1 Hz (90)	Augmentation	10(1w)	AHRS	Original article
			Sham	10	34(7)									
Brunelin, et al. (2012)	2	2	tDCS	15	40.4 (9.9)	35cm^2	Anode: LDLPFC, Cathode: LTPJ	EEG reference points	Yes	2mA	Augmentation	10 (1w)	AHRS, PANSS	Original article
			Sham	15	35.1 (7)									
Dharani et al. 2021	2	2	tDCS	8	39.14 (3.76)	35cm^2	Anode: LDLPFC Cathode: FC1, F7, FC5, AF33	EEG reference points	Yes PNS	2mA	Augmentation	10 (1w)	CDSS	Original article
			Sham	7	33.85 (6.81)									
Dlabac-de Lange, et al. (2015)	2	2	rTMS	16	41.8 (11.6)	75mm figure-of eights coil	BL	EEG reference points	No	10 Hz (90)	Augmentation	30 (3w)	PANSS	Original article
			Sham	14	32.3 (9.7)									
Fröhlich, et al. (2016)	2	2	tDCS	13	43.38 (12.64)	35cm^2	Anode: LDLPFC, Cathode: LTPJ	EEG reference points	Yes	2mA	Augmentation	5 (1w)	AHRS, PANSS	Original article
			Sham	13	40 (10.74)									
Gomes, et al. (2018)	2	2	tDCS	12	39.17 (9.34)	25cm^2	Anode: LDLPFC, Cathode: RDLPFC	EEG reference points	No	2mA	NI	10 (1w)	PANSS, NAB mazes, MCCB	Original article
			Sham	12	33.75 (12.08)									

© 2024 Sabé M et al. *JAMA Network Open.*

Study	Included study arms	Total study arms	Arm	N	Age (SD)	Coil type/ electrode sponge size	Cortical target	Method of locating target	TR	Hz/mA (% MT)	Treatment strategy	Number of sessions (weeks)	Included scales/ tasks	Source of outcome data
**Schizophrenia**														
Guan, et al. (2020)	2	2	rTMS	28	55.5 (7.3)	NI	LDLPFC	MRI	No	20 Hz (110)	Augmentation	40 (8w)	PANSS, RBANS attention	Original article
			Sham	28	49.3 (10.2)									
Gonerva et al. (2022)	2	2	rTMS	31	31(27-42)	Figure-of-eight coil	LTPJ	MRI	Yes	1 HZ (100)	Augmentation	10(1.3w)	PANSS, AHRS	Original article
			Sham	34	34(28-41)									
Huang, et al. (2016)	2	2	rTMS	21	40.58 (3.01)	Figure-of-eight coil	LDLPFC	Located based on motor stimulation	No	10 Hz (110)	Augmentation	21 (3w)	PANSS	Original article
			Sham	20	39.39 (3.03)									
Hoy et al. (2022)	2	2	tDCS	15	45.6 (2.34)	Figure-of-eight coil	LDLPFC	Located based on motor stimulation	No	2 mA	Augmentation	10 (1w)	PANSS	Original article
			Sham	15										
Jeon, et al. (2018)	2	2	tDCS	26	40 (9.41)	$25cm^2$	Anode: LDLPFC, Cathode: RDLPFC	10-20 EEG system	No	2mA	Augmentation	10 (2w)	PANSS, MCCB	Original article
			Sham	30	39.86 (12.42)									
Kantrowitz, et al. (2019)	2	2	tDCS	47	38.2 (9.9)	$39cm^2$	Anode: LDLPFC, Cathode: LTPJ	10-20 EEG system	Yes	2mA	Mixed	10 (2w)	AHRS	Original article
			Sham	42	40.1 (8.6)									
Koops, et al. (2018)	2	2	tDCS	30	44 (11)	$35cm^2$	Anode: LDLPFC, C: LTPJ	EEG reference points	Yes	2mA	Mixed	10 (1w)	AHRS	Original article
			Sham	34	44 (12)									
Kumar, et al. (2020)	2	2	rTMS	50	32.4 (9.2)	Figure-of-eight coil	LDLPFC	5cm rule	No	20 Hz (100)	Mixed	20 (4w)	PANSS	Original article
			Sham	50	30.8 (9.34)									
Lindenmayer, et al. (2019)	2	2	tDCS	15	40.2 (10.69)	$35cm^2$	Anode: LDLPFC, Cathode: LTPJ	EEG reference points	Yes	2mA	Augmentation		PANSS, MCCB	Original article
			Sham	13										

© 2024 Sabé M et al. *JAMA Network Open.*

Study	Included study arms	Total study arms	Arm	N	Age (SD)	Coil type/ electrode sponge size	Cortical target	Method of locating target	TR	Hz/mA (% MT)	Treatment strategy	Number of sessions (weeks)	Included scales/ tasks	Source of outcome data
**Schizophrenia**														
Lisoni et al. (2022)	2	2	tDCS	25	40.96 (13.37)	35cm2	Anode: LDLPFC, Cathode: ROFregion	EEG reference points	No	2mA	Augmentation	15 (3w)	PANSS	Original article
Lisoni et al. (2022)			Sham	25	44.44 (10.97)									
Marquardt et al. (2022)	2	2	tDCS	11	38 (13)	35cm^2	Anode: LDLPFC, Cathode: LTPC	EEG reference points	Yes	2mA	Augmentation	2mA	AHRS	Original article
				10	33(10)									
Paillière-Martinot et al. (2017)	2	2	rTMS	15	21.1(6.79)	Figure-of-eight coil	LTPJ	EEG reference points	Yes	1 Hz (100)	Augmentation	10(2w)	AHRS	Original article
				12	31.25(7.78)									
Prikryl, et al. (2007)	2	2	rTMS	11	31.36 (8.43)	NI	LDLPFC	NI	No	10 Hz (110)	Augmentation	15 (3w)	PANSS	Original article
			Sham	11	36.46 (10.74)									
Prikryl, et al. (2012)	2	2	rTMS	19	30.47 (9.19)	NI	LDLPFC	NI	No	10 Hz (110)	Augmentation	15 (3w)	PANSS	Original article
			Sham	11	34.55 (10.57)									
Prikryl, et al. (2014)	2	2	rTMS	18	30.4 (6.56)	Figure-of-eight coil	LDLPFC	Located based on motor stimulation	No	10 Hz (110)	Augmentation	21 (3w)	PANSS	Original article
			Sham	17	34.58 (10.66)									
Quan, et al. (2015)	2	2	rTMS	78	46.87 (7.87)	90mm circular coil	LDLPFC	Located based on motor stimulation	No	10 Hz (80)	Augmentation	10 (2w)	PANSS	Original article
			Sham	39	46.87 (9.07)									
Singh, et al. (2020)	2	2	rTMS	15	33.3 (9.8)	70mm Figure-of-eight coil	LDLPFC	Located based on motor stimulation	No	20 Hz (100)	Augmentation	20 (4w)	PANSS	Original article
			Sham	15	29.8 (5.7)									
Wen 2021	2	2	rTMS	26	41.4 (7.5)	Figure-of-eight coil	LDLPFC	Located based on motor stimulation	No	10 Hz (110)	Augmentation	20 (4w)	PANSS	Original article
			Sham	26	38.8 (9.1)									

© 2024 Sabé M et al. *JAMA Network Open.*

Study	Included study arms	Total study arms	Arm	N	Age (SD)	Coil type/ electrode sponge size	Cortical target	Method of locating target	TR	Hz/mA (% MT)	Treatment strategy	Number of sessions (weeks)	Included scales/ tasks	Source of outcome data
**Schizophrenia**														
Wobrock, et al. (2015)	2	2	rTMS	76	36.2 (10.5)	Figure-of-eight coil	LDLPFC	EEG reference points	No	10 Hz (110)	Augmentation	15 (3w)	PANSS	Original article
			Sham	81	34.9 (9.1)									
Xiu, et al. (2020)	3	3	rTMS	40	50.7 (9)	Figure-of-eight coil	LDLPFC	MRI	No	10 Hz (110)	Augmentation	40 (8w)	PANSS	Original article
			rTMS	40	52 (10.1)		LDLPFC			20 Hz (110)		40 (8w)		
			Sham	40	54.7 (6.4)									
Xie et al. (2022)	2	2	rTMS	25	31.4(4.46)	Figure-of-eight coil	LTPJ	MRI	Yes	1 hz (110)	Augmentation	15(2.1w)	PANSS, AHRS	Original article
			Sham	30	30.3(6.35)									
Zhao, et al. (2014) (patients with prominent NS)	4	4	rTMS	24	48 (12.2)	Figure-of-eight coil	LDLPFC	Located based on motor stimulation	No	10 Hz (80-110)	Mixed	20 (4w)	PANSS	Original article
			rTMS	23	49.1 (10.6)	Figure-of-eight coil	LDLPFC		No	20 Hz (80-110)	Mixed	20 (4w)		
			rTMS	24	47.7 (11.8)	MF-125 round coil	LDLPFC		No	TBS (80-110)	Mixed	20 (4w)		
			Sham	22	46.7 (13.1)									
Zheng, et al. (2012)	4	4	rTMS	19	56.5 (7.4)	Circular coil	LDLPFC	NI	No	10 Hz (80)	Augmentation	5 (1w)	PANSS, visual spatial working memory	Original article
			rTMS	19	56.8 (5.4)		LDLPFC			20 Hz (80)		5 (1w)		
			iTBS	18	56.4 (9.3)		LDLPFC			50 Hz (80)		5 (1w)		
			Sham	17	55.6 (5.8)									
Zhuo, et al. (2019)	2	2	rTMS	35	28.97 (7.4)	Figure-of-eight coil	LDLPFC	EEG reference points	No	20 Hz (90)	Augmentation	20 (4w)	NAB mazes, WMS: working memory	Original article
			Sham	35	30.63 (8.25)									

© 2024 Sabé M et al. *JAMA Network Open.*

Study	Included study arms	Total study arms	Arm	N	Age (SD)	Coil type/ electrode sponge size	Cortical target	Method of locating target	TR	Hz/mA (% MT)	Treatment strategy	Number of sessions (weeks)	Included scales/ tasks	Source of outcome data
**SUD (tDCS)**														
Alizadehgoradel, et al. (2020)	2	2	tDCS	19	34.31 (9.62)	35cm^2	Anode: LDLPFC Cathode: RDLPFC	10-20 EEG system	No	2mA	Monotherapy	10 (5w)	DDQ	Original article
			Sham	20	35.35 (8.71)									
Alizadehgoradel, et al. (2021)	2	2	tDCS	20	19.43 (1.2)	35cm2	Anode: LDLPFC Cathode: RDLPFC	10-20 EEG system	No	2mA	Monotherapy	12 (6w)	DDQ	Original article
			Sham	19	19.43 (1.15)									
Gaudreault et al. 2021	2	2	tDCS	9	40.4 (10.2)	35cm2	Anode: RDLPFC Cathode: LDLPFC	Pre-measured strap	No	2mA	Monotherapy	15 (5w)	OCDS	
			Sham	8	46.7 (13.9)									
Holla, et al. (2020)	2	2	tDCS	11	38.6 (7.1)	35cm^2	Anode: LDLPFC Cathode: RDLPFC	10-20 EEG system	No	2mA	Monotherapy	5 (1w)	ACQ-SF-R	Original article
			Sham	10	39.4 (7.9)									
Klauss et al. (2018) B	2	2	tDCS	19	35.1 (8.2)	35cm^2	Anode: RDLPFC Cathode: LDLPFC	10-20 EEG system	No	2mA	Monotherapy	19 (2w)	OCDS	Original article
			Sham	16	35 (9.6)									
Martinotti, et al. (2019)	2	2	tDCS	18	40.3 (10.1)	25cm^2	Anode: LDLPFC Cathode: RDLPFC	10-20 EEG system	No	1.5mA	Mixed	5 (1w)	Craving score	Original article
			Sham	16	37.6 (10.9)									
Ekhtiari et al. (2022)	2	2	tDCS	30	34.96	35cm^2	anode/cathode over the F4/Fp1	10-20 EEG system	No	2mA	Monotherapy	1 session	Craving score	Original article
			Sham	30	37.5									

© 2024 Sabé M et al. *JAMA Network Open.*

Study	Included study arms	Total study arms	Arm	N	Age (SD)	Coil type/ electrode sponge size	Cortical target	Method of locating target	TR	Hz/mA (% MT)	Treatment strategy	Number of sessions (weeks)	Included scales/ tasks	Source of outcome data
**SUD (iTBS)**														
Chen et al. (2020)	2	2	iTBS	18	37.72	Figure-of-eight coil	LDLPFC	10–20 EEG system.	No	50 Hz (110)	Augmentation	10 (2w)	DDQ	Original article
			Sham	19	34.95									
Su et al. (2020)	2	2	iTBS	30	31.3	Figure-of-eight coil	LDLPFC	10–20 EEG system.	No	50 Hz (110)	Augmentation	20 (4w)	DDQ	Original article
			Sham	30	33.9									
Chen et al. (2021)	2	2	iTBS	30	29.6	Figure-of-eight coil	LDLPFC	10–20 EEG system.	No	50 Hz (110)	Augmentation	20 (4w)	DDQ	Original article
			Sham	19	30.7									
Su et al. (2022)	2	2	iTBS	20	32.7	Figure-of-eight coil	LDLPFC	10–20 EEG system.	No	50 Hz (110)	Augmentation	20 (4w)	DDQ	Original article
			Sham	20	35									

Abbreviations:

TR = Lifetime treatment resistance. Stimulation techniques; cTBS = Continuous theta-burst stimulation; dTMS = Deep transcranial magnetic stimulation; iTBS = Intermittent theta-burst stimulation; piTBS = Prolonged intermittent theta-burst stimulation; rTMS = repetitive transcranial magnetic stimulation; sTMS = Synchronised transcranial magnetic stimulation; TDCS = Transcranial direct current stimulation. Stimulation sites; BL = Bilateral prefrontal cortex: DLPFC = Dorsolateral prefrontal cortex; DMPFC = Dorsomedial prefrontal cortex; MPFC = Medial prefrontal cortex; OFC = Orbitofrontal cortex; PL = Parietal lobe; SMA = Supplementary motor area; SOA = Supraorbital area; TPJ = Temporal parietal junction; VC = Visual Cortex; VLPFC = Ventrolateral prefrontal cortex. Outcome scales/tasks; ACQ-SF-R = Alcohol Craving Questionnaire – Short Form – Revised; AHRS = Auditory Hallucination Rating Scale; ASRS: Adult ADHD Scale Symptom Checklist; BACS = Brief Assessment of Cognition; CAARS = Conner's Adult ADHD Rating Scale; CAPS = Clinician Administered PTSD Scale for DSM-5; CDRS = Cognitive Drug Computerised Assessment System; Conner's CPT = Conner's Continuous Performance Task; COWAT = Controlled Oral Word Association Task; DDQ = Desires for Drug Questionnaire; HDRS = Hamilton Depression Rating Scale; HRSA = Hamilton Anxiety Rating Scale; MADRS = Montgomery Asberg Depression Rating Scale; MCCB = MATRICS Consensus Cognitive Battery; NAB = Neuropsychological Assessment Battery; OCCS = Obsessive-Compulsive Cocaine Scale; OCDS = Obsessive-Compulsive Drinking Scale; PANSS = Positive and Negative Symptom Scale; PCL-C = Posttraumatic Checklist – Civilian; PCL-M = Posttraumatic Checklist – Military; RBANS = Repeatable Battery for the Assessment of Neuropsychological Status; SANS = Scale for the Assessment of Negative Symptoms; SAPS = Scale for the Assessment of Positive Symptoms; TMT-A = Trail making task – part A; TMT-B = Trail making task – part B; TOL = Tower of London Task; TVA = Theory of visual attention; WMS = Weschler Memory Scale; Y-BOCS = Yale-Brown Obsessive Compulsive Scale. [1] treatment groups were combined. [2] sham groups were combined. [3] electrode placements based on 10-20 EEG system. [4] Cirillo P, Gold AK, Nardi AE, et al. Transcranial magnetic stimulation in anxiety and trauma-related disorders: A systematic review and meta-analysis. *Brain Behav*. 2019;9(6):e01284. [5] Mutz J, Vipulananthan V, Carter B,

© 2024 Sabé M et al. *JAMA Network Open.*

Hurlemann R, Fu CHY, Young AH. Comparative efficacy and acceptability of non-surgical brain stimulation for the acute treatment of major depressive episodes in adults: systematic review and network meta-analysis. *BMJ*. 2019;364:l1079. [6] Perera MPN, Mallawaarachchi S, Miljevic A, Bailey NW, Herring SE, Fitzgerald PB. Repetitive Transcranial Magnetic Stimulation for Obsessive-Compulsive Disorder: A Meta-analysis of Randomized, Sham-Controlled Trials. Biol Psychiatry Cogn Neurosci Neuroimaging. 2021;6(10):947-960. [7] Kan RLD, Zhang BBB, Zhang JJQ, Kranz GS. Non-invasive brain stimulation for posttraumatic stress disorder: a systematic review and meta-analysis. Transl Psychiatry. 2020;10(1):168. [8] Ma T, Sun Y, Ku Y. Effects of Non-invasive Brain Stimulation on Stimulant Craving in Users of Cocaine, Amphetamine, or Methamphetamine: A Systematic Review and Meta-Analysis. Front Neurosci. 2019;13:10

© 2024 Sabé M et al. *JAMA Network Open.*

eTable 2. Heterogeneity Assessments With the Variance-Partition-Coefficient (VPC) for the Primary Outcome

VPC are expressed as proportion [0-1]. The percentage of heterogeneity can be obtained by multiplying this coefficient by 100.

Schizophrenia

TMS for schizophrenia

Negative symptoms of schizophrenia

a. HF-LDLPFC, N=14, n=909

VPCs is over 75%, indicating high level of heterogeneity

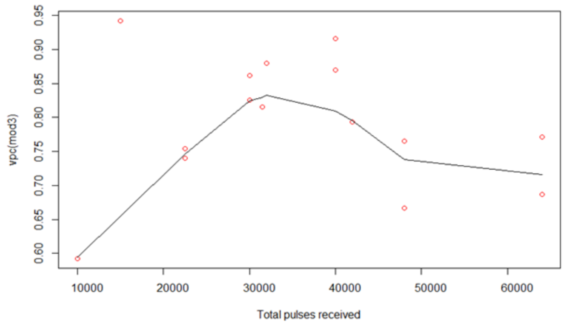

b. BLDLPFC, N=2, n=57

There is a considerable level of heterogeneity with VPCs up to 95%

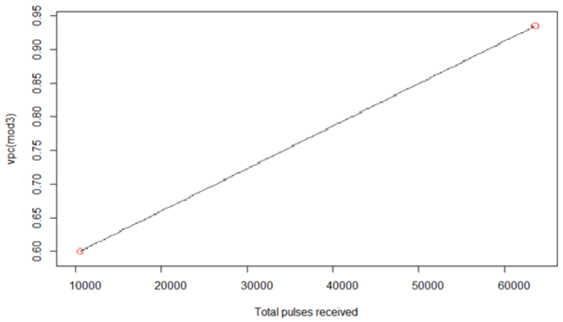

Positive symptoms of schizophrenia

c. HF-LDLPFS, N=14, n=909

There is a considerable level of heterogeneity with VPCs up to 85%

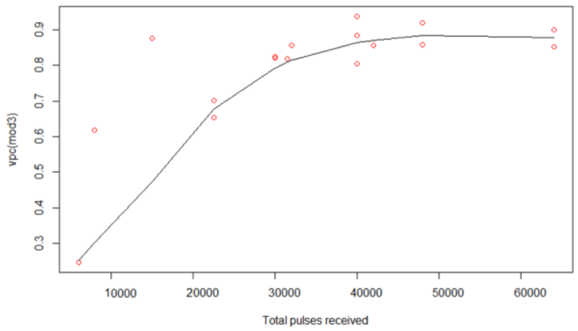

d. BLDLPFC, N=2, n=57

There is a considerable level of heterogeneity with VPCs up to 95%

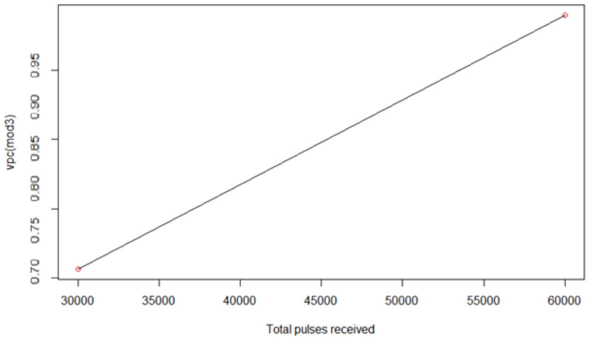

Treatment-resistant hallucinations of patients with schizophrenia (AHRS)

e. LF-LTPJ, N=6, n=209

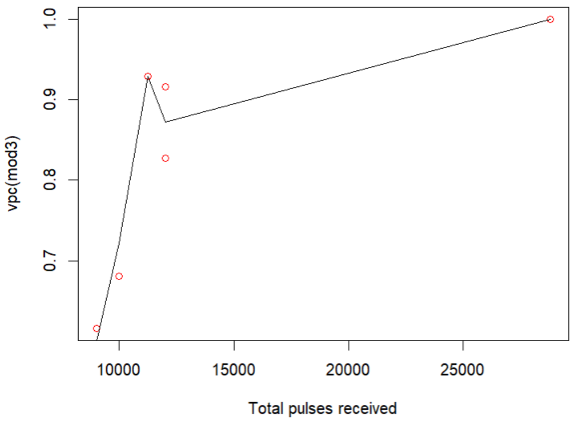

© 2024 Sabé M et al. *JAMA Network Open.*

tDCS for Schizophrenia

Negative symptoms of schizophrenia	Positive symptoms of schizophrenia
**f. LDLPFS,** N=5, n=142	**g. LDLPFS,** N=5, n=142

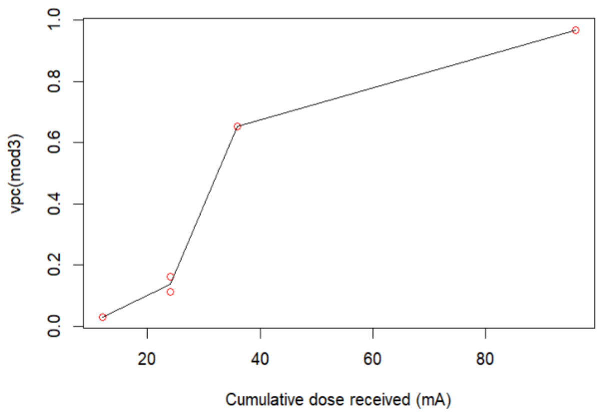

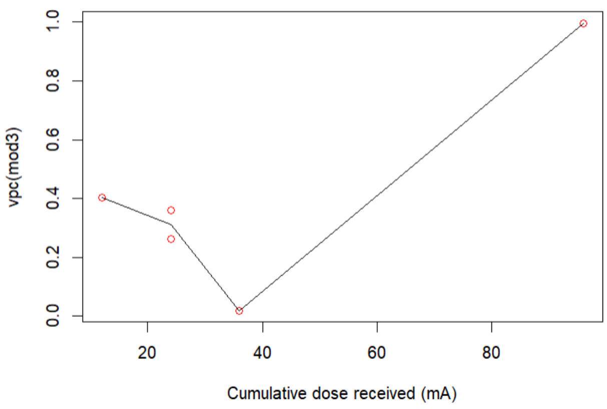

tDCS

Treatment-resistant hallucinations of patients with schizophrenia (AHRS)

h. HF-LDLPFC, N=6, n= 242

There is a considerable level of heterogeneity with VPCs up to 95%

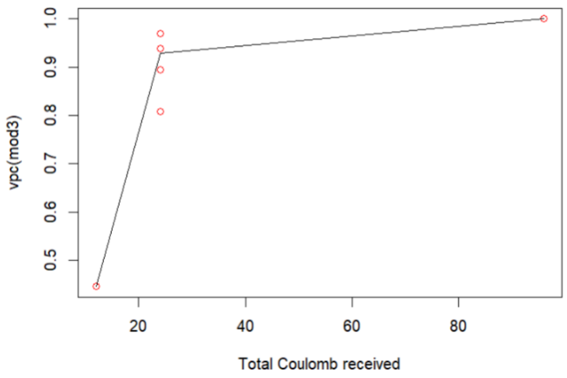

TMS

© 2024 Sabé M et al. *JAMA Network Open.*

Depressive symptoms reduction for treatment-resistant patients with depression

i. HF-LDLPFC, N=22, n=1047

There is a considerable level of heterogeneity with VPCs up to 90%

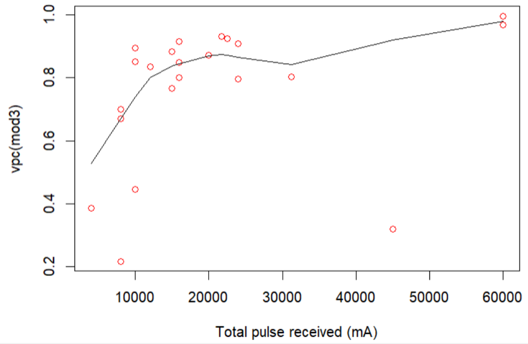

j. BDLPFC, N=4, n=178

There is a considerable level of heterogeneity with VPCs up to 95%

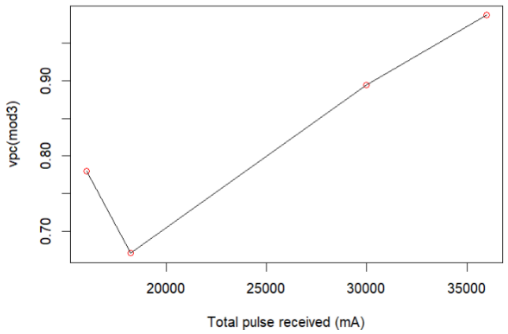

k. LF-RDLPFC, N=4, n=102

There is a considerable level of heterogeneity with VPCs up to 95%

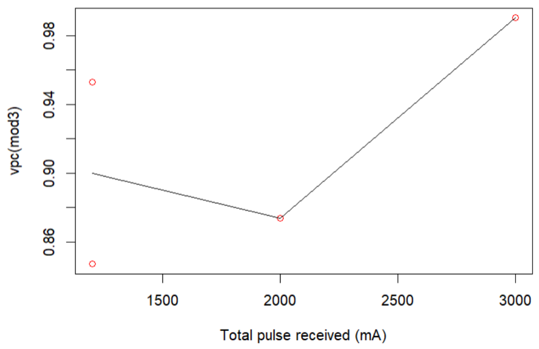

TMS for depression

l. HF-LDLPFC, N=4 , n=89

There is a considerable level of heterogeneity with VPCs up to 85%

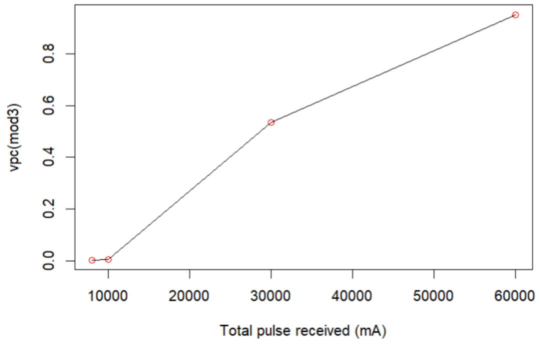

m. HF-RDLPFC, N=2, n=97

There is a considerable level of heterogeneity with VPCs up to 95%

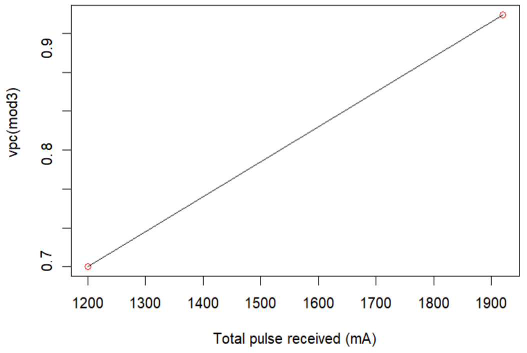

TMS for bipolar depression

n. LDLPFC, N=2, n=42

© 2024 Sabé M et al. *JAMA Network Open.*

There is a considerable level of heterogeneity with VPCs up to 95%

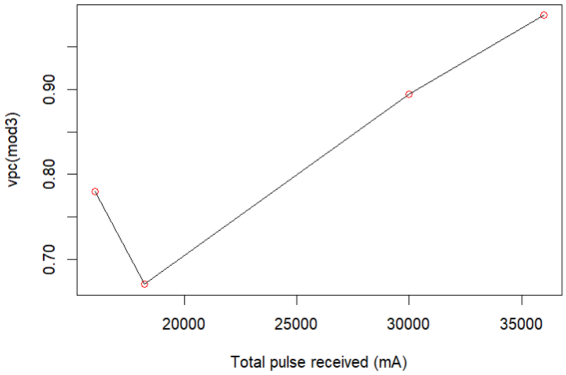

tDCS for depression

o. LDLPFC, N=6, n=265

There is a considerable level of heterogeneity with VPCs up to 95%

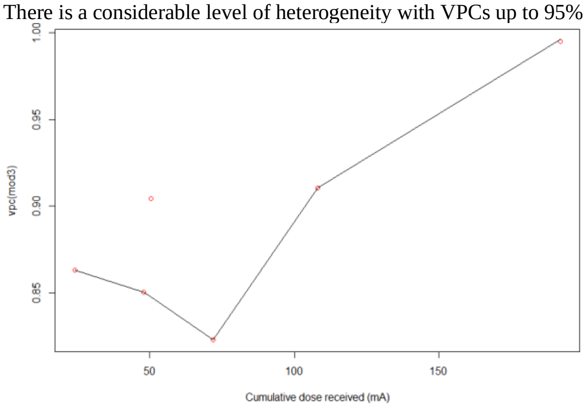

TMS for OCD

p. LF-RDLPFC, N=5, n=138

There is a moderate level of heterogeneity with VPCs up to 60%

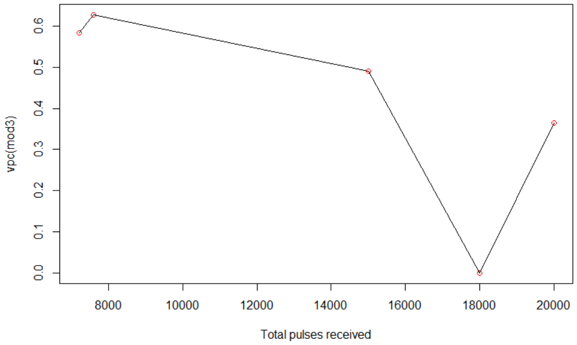

q. HF-RDLPFC, N=2, n=57

There is a considerable level of heterogeneity with VPCs up to 95%

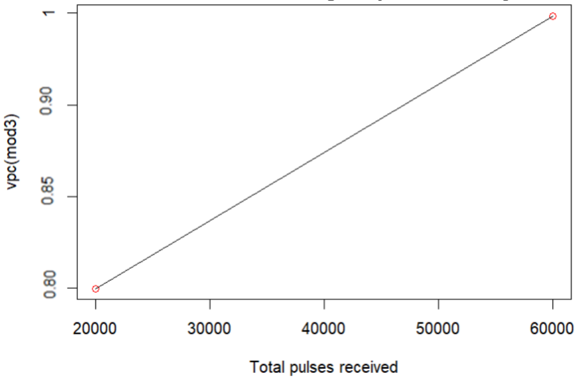

r. LF-SMD, N=4, n=116

There is a considerable level of heterogeneity with VPCs up to 95%

s. LF-OFC, N=3, n=85

There is a moderate level of heterogeneity with VPCs up to 60%

© 2024 Sabé M et al. *JAMA Network Open.*

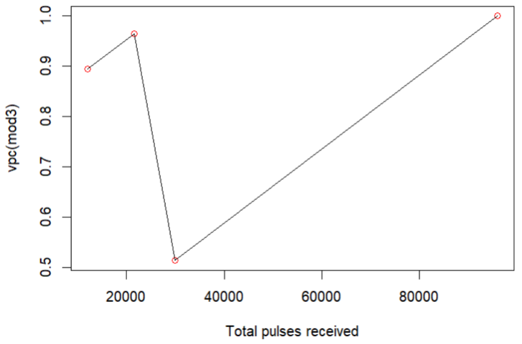

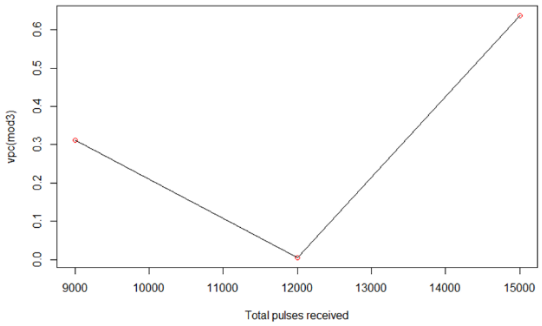

tDCS for SUD

t. LDLPFC, N=4, n=116

There is a moderate level of heterogeneity with VPCs up to 60%

v. LDLFPC ; N=7, n=242

There is a moderate level of heterogeneity with VPCs up to 60%

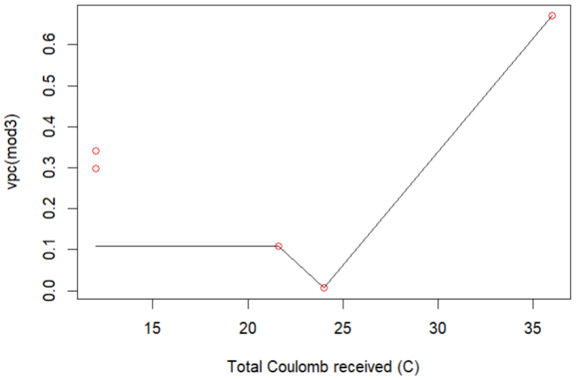

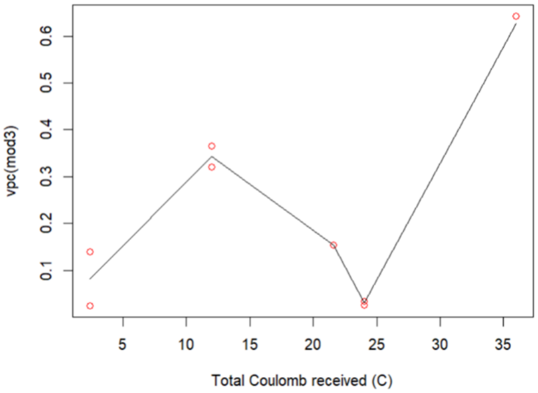

iTBS for SUD

w. LDLPFC, N=4, n=186

There is a considerable level of heterogeneity with VPCs up to 70%, but that only concerns low doses of pulses

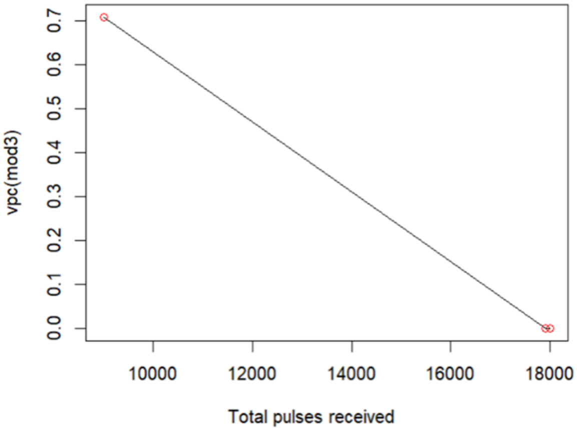

TMS for PTSD

x. LF-RDLPFC, N=3, n=49

There is a moderate level of heterogeneity with VPCs up to 45%

y. HF-RDLPFC, N=2, n=53

There is a considerable level of heterogeneity with VPCs up to 95%

© 2024 Sabé M et al. *JAMA Network Open.*

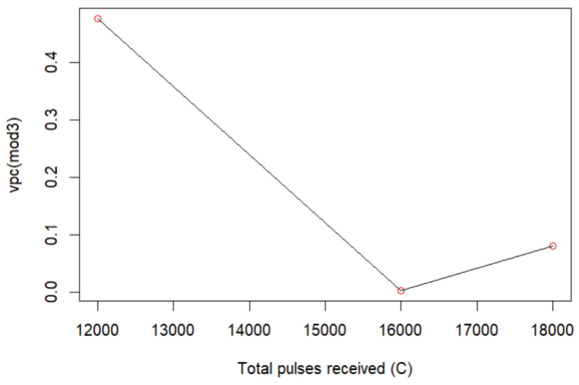

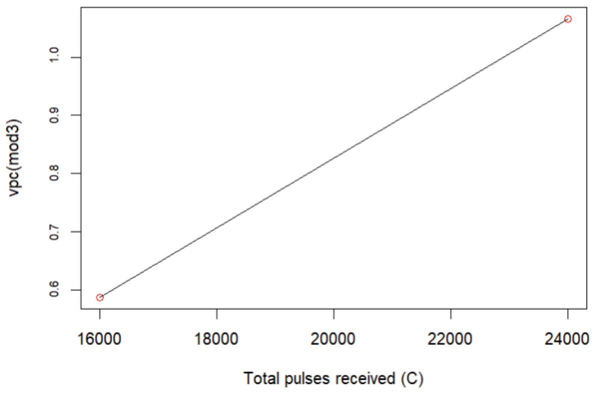

eTable 3. Risk-of-Bias Assessments

	**Risk of bias arising from the randomisation process**	**Risk of bias due deviations from the intended interventions**	**Missing outcome data**	**Risk of bias in measurement of outcome**	**Risk of bias in selection of the reported result**	**Overall risk of bias**
**1.Alizadehgoradel 2020** (Alizadehgoradel et al., 2020)	Some concerns	Low	Some concerns	Low	Some concerns	Some concerns
**2.Alizadehgoradel 2021** (Alizadehgoradel et al., 2021)	Some concerns	Low	Some concerns	Low	Some concerns	Some concerns
**3.Ahmadizadeh 2018** (Ahmadizadeh & Rezaei, 2018)	Low	Low	Low	Low	Some concerns	Some concerns
**4.Alonso 2001** (Alonso et al., 2001)	Some concerns	Low	Low	Low	Some concerns	Some concerns
**5.Anderson 2007** (Anderson et al., 2007)	Low	High	Some concerns	Low	Some concerns	High
**6.Arumugham 2018** (Arumugham et al., 2018)	Low	Low	Low	Low	Low	Low
**7.Asl 2022** (Asl & Vaghef, 2022)	Some concerns	Low	Low	Low	Low	Low
**8.Avery 1999** (Avery et al., 1999)		Low	Low	Low	Some concerns	Some concerns
**9.Badawy 2010** (Badawy, AA; ElSawy, H; ElHay, 2010)	Some concerns	Low	Low	Low	Some concerns	Some concerns
**10.Baeken 2013** (Baeken et al., 2013)	Some concerns	Low	Low	Low	Some concerns	Low
**11.Bais 2014** (Bais et al., 2014)	Some concerns	Low	Low	Low	Some concerns	Low
**12.Bakim 2012** (Bakim et al., 2012)	Low	Low	Some concerns	Low	Some concerns	Some concerns

© 2024 Sabé M et al. *JAMA Network Open.*

	Risk of bias arising from the randomisation process	Risk of bias due deviations from the intended interventions	Missing outcome data	Risk of bias in measurement of outcome	Risk of bias in selection of the reported result	Overall risk of bias
**13.Barr 2012** (Barr et al., 2012)	Some concerns	Low	Some concerns	Low	Some concerns	Some concerns
**14.Berman 2000** (Berman et al., 2000)	Some concerns	Low	Low	Low	Some concerns	Some concerns
**15.Blumberger 2012A** (Blumberger, Tran, et al., 2012)	Some concerns	Low	Low	Low	Some concerns	Some concerns
**16.Boggio 2010** (Boggio et al., 2010)	Some concerns	Low	Some concerns	Low	Some concerns	Some concerns
**17.Bortolomasi 2007** (Bortolomasi et al., 2007)	Low	Low	Low	Low	Some concerns	Some concerns
**18.Bose 2018** (Bose et al., 2018)	Low	Low	Low	Low	Some concerns	Some concerns
**19.Boutros 2002** (Boutros et al., 2002)	Low	Low	Low	Low	Some concerns	Some concerns
**20.Brunelin 2006** (Brunelin et al., 2006)	Some concerns	Low	Some concerns	Low	Some concerns	Some concerns
**21.Brunelin 2012** (Brunelin et al., 2012)	Low	Low	Low	Low	Some concerns	Some concerns
**22.Chen 2013** (S.-J. Chen et al., 2013)	Low	Low	Low	Low	Some concerns	Some concerns
**23.Chen et al. 2020** (Chen et al., 2020)	Low	Low	Low	Low	Some concerns	Some concerns
**24.Chen et al. 2021** (Chen et al., 2021)	Low	Low	Low	Low	Some concerns	Some concerns
**25.Dlabac DeLange2015** (Dlabac-de Lange et al., 2015)	Some concerns	Low	Low	Low	Some concerns	Some concerns
**26.Dharani 2021** (Dharani et al., 2021)	Low	Low	Some concerns	Low	Some concerns	Some concerns
**27.Ekhtiari 2022** (Ekhtiari et al., 2022)	Low	Low	Low	Low	Some concerns	Some concerns
**28.Elbeh 2016** (Elbeh et al., 2016)	Low	Low	Low	Low	Some concerns	Some concerns
**29.Eschweiler 2000** (Eschweiler et al., 2000)	Some concerns	Some concerns	High	Low	Some concerns	High

© 2024 Sabé M et al. *JAMA Network Open.*

**30.Fitzgerald 2003** (Fitzgerald et al., 2003)	Low	Low	Low	Low	Some concerns	Some concerns
**31.Fitzgerald 2012** (Fitzgerald et al., 2012)	Low	High	High	Low	Some concerns	High
**32.Frohlich 2016** (Fröhlich et al., 2016)	Low	Low	Low	Low	Some concerns	Some concerns
**33.Gaudreault 2021** (Gaudreault et al., 2021)	Low	High	High	Low	Some concerns	High
	**Risk of bias arising from the randomisation process**	**Risk of bias due deviations from the intended interventions**	**Missing outcome data**	**Risk of bias in measurement of outcome**	**Risk of bias in selection of the reported result**	**Overall risk of bias**
**34.George 2010** (Mark S George et al., 2010)	Low	High	High	Low	Some concerns	High
**35.George 1997** (M S George et al., 1997)	Low	Low	Low	Low	Some concerns	Some concerns
**36.George 2000** (M S George et al., 2000)	Low	Low	Low	Low	Some concerns	Some concerns
**37.Gomes 2012** (P. V. O. Gomes et al., 2012)	Some concerns	Some concerns	Low	Low	Some concerns	Some concerns
**38.Gomes 2018** (J. S. Gomes et al., 2018)	Some concerns	Some concerns	Low	Low	Some concerns	Some concerns
**39.Gonerva 2022** (Gornerova et al., 2023)	Low	Low	Low	Low	Low	Low
**40.Guan 2020** (Guan et al., 2020)	Low	Low	High	Low	Some concerns	Some concerns
**41.Haghigi 2015** (Haghighi et al., 2015)	Low	Low	Low	Low	Some concerns	Some concerns
**42.Holla 2020** (Holla et al., 2020)	Low	Low	Low	Low	Some concerns	Some concerns
**43.Holtzheimer 2004** (Holtzheimer et al., 2004)	Low	Low	Some concerns	Low	Some concerns	Some concerns
**44.Hoppner 2003** (Höppner et al., 2003)	Some concerns	Low	Low	Low	Some concerns	Some concerns
**45.Hoy 2022** (Hoy et al., 2022)	Low	Low	Low	Low	Low	Low
**46.Huang 2016** (Huang et al., 2016)	Low	Low	Some concerns	Low	Some concerns	Some concerns
**47.Januel 2006** (Januel et al., 2006)	Some concerns	Low	Low	Low	Some concerns	Some concerns

© 2024 Sabé M et al. *JAMA Network Open.*

**48.Jahangard 2016** (Jahangard et al., 2016)	Low	Low	Low	Low	Some concerns	Some concerns
**49.Jeon 2018** (Jeon et al., 2018)	Low	Some concerns	Low	Low	Some concerns	Some concerns
**50.Kauffman 2004** (Kauffmann et al., 2004)	Some concerns	Low	Low	Low	Some concerns	Some concerns
	**Risk of bias arising from the randomisation process**	**Risk of bias due deviations from the intended interventions**	**Missing outcome data**	**Risk of bias in measurement of outcome**	**Risk of bias in selection of the reported result**	**Overall risk of bias**
**51.Kang 2009** (Kang et al., 2009)	Low	Low	Low	Low	Some concerns	Some concerns
**52.Kantrowitz 2019** (Kantrowitz et al., 2019)	Low	Low	Low	Low	Low	Low
**53.Klauss 2018B** (Klauss et al., 2018)	Low	Low	Low	Low	Low	Low
**54.Klein 1999A** (Klein et al., 1999)	Some concerns	Low	Low	Low	Some concerns	Some concerns
**55. Khedr 2022** (Khedr et al., 2022)	Low	Low	Low	Low	Some concerns	Some concerns
**56.Koops 2018** (Koops et al., 2018)	Low	Low	Low	Low	Low	Low
**57.Kreuzer 2015** (Kreuzer et al., 2015)	Low	Low	High	Low	Low	High
**58.Kumar 2020** (Kumar et al., 2020)	Low	High	Low	Low	Some concerns	High
**59.Loo 2010** (Loo et al., 2010)	Low	High	High	Low	Low	High
**60.Loo 2012** (Loo et al., 2012)	Low	Low	Low	Low	Low	Low
**61.Loo 2018** (Loo et al., 2018)	Low	Low	Low	Low	Low	Low
**62.Lindenmayer 2019** (Lindenmayer et al., 2019)	Low	Low	Low	Low	Some concerns	Some concerns
**63.Lisoni 2022** (Lisoni et al., 2022)	Low	Low	Low	Low	Low	Low
**64.Ma 2014** (Ma et al., 2014)	Low	Low	Some concerns	Low	Some concerns	Some concerns

© 2024 Sabé M et al. *JAMA Network Open.*

**65.Mansur 2011** (Mansur et al., 2011)	Low	Low	High	Low	Some concerns	High
	**Risk of bias arising from the randomisation process**	**Risk of bias due deviations from the intended interventions**	**Missing outcome data**	**Risk of bias in measurement of outcome**	**Risk of bias in selection of the reported result**	**Overall risk of bias**
**66.Mantovani 2010** (Mantovani et al., 2010)	Low	Low	Low	Low	Some concerns	Some concerns
**67.Martinotti 2019** (Martinotti et al., 2019)	Low	Low	Low	Low	Some concerns	Some concerns
**68.Marquardt 2022** (Marquardt et al., 2022)	Low	Low	Low	Low	Some concerns	Some concerns
**69.Moirand 2022** (Moirand et al., 2022)	Low	Low	Low	Low	Low	Low
**70.McDonald 2006** (McDonald et al., 2006)	Some concerns	Low	High	Low	Some concerns	High
**71.Mogg 2008** (Mogg et al., 2008)	Low	Some concerns	Low	Low	Some concerns	Some concerns
**72.Nam 2013** (Nam et al., 2013)	Low	Low	Low	Low	Some concerns	Some concerns
**73. Nauczyciel 2014** (Nauczyciel et al., 2014)	Some concerns	Low	Low	Low	Some concerns	Some concerns
**74.O'reardon 2007** (O'Reardon et al., 2007)	Low	High	Low	Low	Some concerns	High
**75.Palliere-Martinot 2010** (Paillère Martinot et al., 2010)	Low	Low	Low	Low	Some concerns	Some concerns
**76.Palliere-Martinot 2017** (Paillère-Martinot et al., 2016)	Low	Low	Low	Low	Some concerns	Some concerns
**77.Padberg 2002** (Frank Padberg et al., 2002)	Low	Low	Low	Low	Some concerns	Some concerns
**78.Padberg 1999** (F Padberg et al., 1999)	Low	Low	Low	Low	Some concerns	Some concerns
**79.Pelissolo 2016** (Pelissolo et al., 2016)	Low	Low	Low	Low	Some concerns	Some concerns
**80.Prasko 2006** (Prasko et al., 2006)	Low	Low	Low	Low	Some concerns	Some concerns

© 2024 Sabé M et al. *JAMA Network Open.*

**81.Prasser 2015** (Prasser et al., 2015)	Low	Some concerns	Low	Low	Some concerns	Some concerns
	**Risk of bias arising from the randomisation process**	**Risk of bias due deviations from the intended interventions**	**Missing outcome data**	**Risk of bias in measurement of outcome**	**Risk of bias in selection of the reported result**	**Overall risk of bias**
**82.Prikryl 2007** (Prikryl et al., 2007)	Low	Low	Low	Low	Some concerns	Some concerns
**83.Prikryl 2012** (Prikryl et al., 2012)	Low	Low	Low	Low	Some concerns	Some concerns
**84.Prikryl 2014** (Prikryl et al., 2014)	Low	High	High	Low	Some concerns	High
**85.Quan 2015** (Quan et al., 2015)	Some concerns	Low	Low	Low	Some concerns	Some concerns
**86. Ruffini et al. 2009** (Ruffini et al., 2009)	Low	Low	Some concerns	Low	Some concerns	Some concerns
**87.Sachdev 2007** (Sachdev et al., 2007)	Low	Low	Low	Low	Some concerns	Some concerns
**88.Salehinejad 2015** (Salehinejad, 2015)	Some concerns	Low	Low	Low	Some concerns	Some concerns
**89.Salehinejad 2017** (Salehinejad et al., 2017)	Low	Low	Low	Low	Some concerns	Some concerns
**90.Sampaio Junior 2018** (Sampaio-Junior et al., 2018)	Low	Low	Low	Low	Low	Low
**91.Shayganfard 2016** (Shayganfard et al., 2016)	Low	Low	Low	Low	Some concerns	Some concerns
**92.Singh 2020** (Singh et al., 2020)	Low	Some concerns	Low	Low	Some concerns	Some concerns
**93.Speer 2005** (Speer et al., 2005)	Some concerns	Some concerns	Low	Low	Some concerns	Some concerns
**94. Su 2005** (Su et al., 2005)	Some concerns	Some concerns	Low	Low	Some concerns	Some concerns
**95.Su 2017** (Su et al., 2017)	Some concerns	Low	Some concerns	Low	Some concerns	Some concerns
**96. Su 2020** (Su et al., 2020)	Low	Low	Low	Low	Some concerns	Some concerns
**97.Taylor 2018** (Taylor et al., 2018)	Some concerns	Some concerns	High	Low	Some concerns	High
**98.Triggs 2010** (Triggs et al., 2010)	Low	Low	Low	Low	Some concerns	Some concerns

© 2024 Sabé M et al. *JAMA Network Open.*

	**Risk of bias arising from the randomisation process**	**Risk of bias due deviations from the intended interventions**	**Missing outcome data**	**Risk of bias in measurement of outcome**	**Risk of bias in selection of the reported result**	**Overall risk of bias**
**99.Tsai 2022** (Tsai et al., 2022)	Low	Low	Low	Low	Some concerns	Some concerns
**100.Tong 2021** (Tong et al., 2021)	Low	Low	Low	Low	Some concerns	Some concerns
**101.Van Eijndhoven 2020** (van Eijndhoven et al., 2020)	Some concerns	Low	Low	Low	Some concerns	Some concerns
**102. Wen 2021** (Wen et al., 2021)	Low	Some concerns	Some concerns	Low	Some concerns	Some concerns
**103. Watts 2012** (Watts et al., 2012)	Low	Low	Low	Low	Some concerns	Some concerns
**104. Wobrock 2015** (Wobrock et al., 2015)	Low	Some concerns	Some concerns	Low	Some concerns	Some concerns
**105.Xie 2022** (Xie et al., 2023)	Low	Low	Low	Low	Some concerns	Some concerns
**106. Xiu 2020** (Xiu et al., 2020)	Low	Low	Some concerns	Low	Low	Some concerns
**107.Zhao 2014** (Zhao et al., 2014)	Low	Low	Low	Some concerns	Some concerns	Some concerns
**108.Zheng 2010** (Zheng et al., 2010)	Low	Low	Low	Low	Some concerns	Some concerns
**109.Zheng 2012** (Zheng et al., 2012)	Low	Low	Low	Low	Some concerns	Some concerns
**110.Zhou 2019** (Zhuo et al., 2019)	Low	Low	Some concerns	Low	Low	Some concerns

© 2024 Sabé M et al. *JAMA Network Open.*

eReferences. List of all included studies

Ahmadizadeh, M.-J., & Rezaei, M. (2018). Unilateral right and bilateral dorsolateral prefrontal cortex transcranial magnetic stimulation in treatment post-traumatic stress disorder: A randomized controlled study. *Brain Research Bulletin*, *140*, 334–340. https://doi.org/10.1016/j.brainresbull.2018.06.001

Alizadehgoradel, J., Imani, S., Nejati, V., Vanderhasselt, M.-A., Molaei, B., Salehinejad, M. A., Ahmadi, S., & Taherifard, M. (2021). Improved Executive Functions and Reduced Craving in Youths with Methamphetamine Addiction: Evidence from Combined Transcranial Direct Current Stimulation with Mindfulness Treatment. *Clinical Psychopharmacology and Neuroscience : The Official Scientific Journal of the Korean College of Neuropsychopharmacology*, *19*(4), 653–668. https://doi.org/10.9758/cpn.2021.19.4.653

Alizadehgoradel, J., Nejati, V., Sadeghi Movahed, F., Imani, S., Taherifard, M., Mosayebi-Samani, M., Vicario, C. M., Nitsche, M. A., & Salehinejad, M. A. (2020). Repeated stimulation of the dorsolateral-prefrontal cortex improves executive dysfunctions and craving in drug addiction: A randomized, double-blind, parallel-group study. *Brain Stimulation: Basic, Translational, and Clinical Research in Neuromodulation*, *13*(3), 582–593. https://doi.org/10.1016/j.brs.2019.12.028

Alonso, P., Pujol, J., Cardoner, N., Benlloch, L., Deus, J., Menchón, J. M., Capdevila, A., & Vallejo, J. (2001). Right prefrontal repetitive transcranial magnetic stimulation in obsessive-compulsive disorder: a double-blind, placebo-controlled study. *The American Journal of Psychiatry*, *158*(7), 1143–1145. https://doi.org/10.1176/appi.ajp.158.7.1143

Anderson, I. M., Delvai, N. A., Ashim, B., Ashim, S., Lewin, C., Singh, V., Sturman, D., & Strickland, P. L. (2007). Adjunctive fast repetitive transcranial magnetic stimulation in depression. *The British Journal of Psychiatry : The Journal of Mental Science*, *190*, 533–534. https://doi.org/10.1192/bjp.bp.106.028019

Arumugham, S. S., Vs, S., Hn, M., B, V., Ravi, M., Sharma, E., Thirthalli, J., & Reddy, Y. C. J. (2018). Augmentation Effect of Low-Frequency Repetitive Transcranial Magnetic Stimulation Over Presupplementary Motor Area in Obsessive-Compulsive Disorder: A Randomized Controlled Trial. *The Journal of ECT*, *34*(4), 253–257. https://doi.org/10.1097/YCT.0000000000000509

Asl, F. A., & Vaghef, L. (2022). The effectiveness of high-frequency left DLPFC-rTMS on depression, response inhibition, and cognitive flexibility in female subjects with major depressive disorder. *Journal of Psychiatric Research*, *149*, 287–292.

Avery, D. H., Claypoole, K., Robinson, L., Neumaier, J. F., Dunner, D. L., Scheele, L., Wilson, L., & Roy-Byrne, P. (1999). Repetitive transcranial magnetic stimulation in the treatment of medication-resistant depression: preliminary data. *The Journal of Nervous and Mental Disease*, *187*(2), 114–117. https://doi.org/10.1097/00005053-199902000-00009

Badawy, AA; ElSawy, H; ElHay, M. (2010). Efficacy of repetitive transcranial magnetic stimulation in the management of obsessive compulsive disorder. *Egyptian Journal of Neurology, Psychiatry and Neurosurgery*, *44*, 393–397.

Baeken, C., Vanderhasselt, M.-A., Remue, J., Herremans, S., Vanderbruggen, N., Zeeuws, D., Santermans, L., & De Raedt, R. (2013). Intensive HF-rTMS treatment in refractory medication-resistant unipolar depressed patients. *Journal of Affective Disorders*, *151*(2), 625–631. https://doi.org/10.1016/j.jad.2013.07.008

Bais, L., Vercammen, A., Stewart, R., F.D., V., Visser, B., Aleman, A., & Knegtering, R. (2014). Short and Long Term Effects of Left and Bilateral Repetitive Transcranial Magnetic Stimulation in Schizophrenia Patients with Auditory Verbal Hallucinations: A Randomized Controlled Trial. *PloS*

© 2024 Sabé M et al. *JAMA Network Open.*

One, *9*, e108828. https://doi.org/10.1371/journal.pone.0108828

Bakim, B., Uzun, U. E., Karamustafalioglu, O., Ozcelik, B., Alpak, G., Tankaya, O., Cengiz, Y., & Yavuz, B. G. (2012). The Combination of Antidepressant Drug Therapy and High-Frequency Repetitive Transcranial Magnetic Stimulation in Medication-Resistant Depression. *Klinik Psikofarmakoloji Bülteni-Bulletin of Clinical Psychopharmacology*, *22*(3), 244–253. https://doi.org/10.5455/bcp.20120807092434

Barr, M. S., Farzan, F., Tran, L. C., Fitzgerald, P. B., & Daskalakis, Z. J. (2012). A randomized controlled trial of sequentially bilateral prefrontal cortex repetitive transcranial magnetic stimulation in the treatment of negative symptoms in schizophrenia. *Brain Stimulation*, *5*(3), 337–346. https://doi.org/10.1016/j.brs.2011.06.003

Berman, R. M., Narasimhan, M., Sanacora, G., Miano, A. P., Hoffman, R. E., Hu, X. S., Charney, D. S., & Boutros, N. N. (2000). A randomized clinical trial of repetitive transcranial magnetic stimulation in the treatment of major depression. *Biological Psychiatry*, *47*(4), 332–337. https://doi.org/10.1016/s0006-3223(99)00243-7

Blumberger, D. M., Tran, L. C., Fitzgerald, P. B., Hoy, K. E., & Daskalakis, Z. J. (2012). A randomized double-blind sham-controlled study of transcranial direct current stimulation for treatment-resistant major depression. *Frontiers in Psychiatry*, *3*, 74. https://doi.org/10.3389/fpsyt.2012.00074

Boggio, P. S., Rocha, M., Oliveira, M. O., Fecteau, S., Cohen, R. B., Campanhã, C., Ferreira-Santos, E., Meleiro, A., Corchs, F., Zaghi, S., Pascual-Leone, A., & Fregni, F. (2010). Noninvasive brain stimulation with high-frequency and low-intensity repetitive transcranial magnetic stimulation treatment for posttraumatic stress disorder. *The Journal of Clinical Psychiatry*, *71*(8), 992–999. https://doi.org/10.4088/JCP.08m04638blu

Bortolomasi, M., Minelli, A., Fuggetta, G., Perini, M., Comencini, S., Fiaschi, A., & Manganotti, P. (2007). Long-lasting effects of high frequency repetitive transcranial magnetic stimulation in major depressed patients. *Psychiatry Research*, *150*(2), 181–186. https://doi.org/10.1016/j.psychres.2006.04.010

Bose, A., Shivakumar, V., Agarwal, S. M., Kalmady, S. V, Shenoy, S., Sreeraj, V. S., Narayanaswamy, J. C., & Venkatasubramanian, G. (2018). Efficacy of fronto-temporal transcranial direct current stimulation for refractory auditory verbal hallucinations in schizophrenia: A randomized, double-blind, sham-controlled study. *Schizophrenia Research*, *195*, 475–480. https://doi.org/10.1016/j.schres.2017.08.047

Boutros, N. N., Gueorguieva, R., Hoffman, R. E., Oren, D. A., Feingold, A., & Berman, R. M. (2002). Lack of a therapeutic effect of a 2-week sub-threshold transcranial magnetic stimulation course for treatment-resistant depression. *Psychiatry Research*, *113*(3), 245–254. https://doi.org/10.1016/s0165-1781(02)00267-6

Brunelin, J., Mondino, M., Gassab, L., Haesebaert, F., Gaha, L., Suaud-Chagny, M.-F., Saoud, M., Mechri, A., & Poulet, E. (2012). Examining transcranial direct-current stimulation (tDCS) as a treatment for hallucinations in schizophrenia. *The American Journal of Psychiatry*, *169*(7), 719–724. https://doi.org/10.1176/appi.ajp.2012.11071091

Brunelin, J., Poulet, E., Bediou, B., Kallel, L., Daléry, J., d'Amato, T., & Saoud, M. (2006). Low frequency repetitive transcranial magnetic stimulation improves source monitoring deficit in hallucinating patients with schizophrenia. *Schizophrenia Research*, *81*, 41–45. https://doi.org/10.1016/j.schres.2005.10.009

Chen, S.-J., Chang, C.-H., Tsai, H.-C., Chen, S.-T., & Lin, C. C. (2013). Superior antidepressant effect occurring 1 month after rTMS: add-on rTMS for subjects with medication-resistant depression.

Neuropsychiatric Disease and Treatment, *9*, 397–401. https://doi.org/10.2147/NDT.S40466

Chen, T., Su, H., Li, R., Jiang, H., Li, X., Wu, Q., Tan, H., Zhang, J., Zhong, N., Du, J., Gu, H., & Zhao, M. (2020). The exploration of optimized protocol for repetitive transcranial magnetic stimulation in the treatment of methamphetamine use disorder: A randomized sham-controlled study. *EBioMedicine*, *60*. https://doi.org/10.1016/j.ebiom.2020.103027

Chen, T., Su, H., Wang, L., Li, X., Wu, Q., Zhong, N., Du, J., Meng, Y., Duan, C., Zhang, C., Shi, W., Xu, D., Song, W., Zhao, M., & Jiang, H. (2021). Modulation of Methamphetamine-Related Attention Bias by Intermittent Theta-Burst Stimulation on Left Dorsolateral Prefrontal Cortex . In *Frontiers in Cell and Developmental Biology* (Vol. 9). https://www.frontiersin.org/articles/10.3389/fcell.2021.667476

Dharani, R., Goyal, N., Mukherjee, A., & Umesh, S. (2021). Adjuvant High-Definition Transcranial Direct Current Stimulation for Negative Symptoms in Schizophrenia: A Pilot Study. *The Journal of ECT*, *37*(3), 195–201. https://doi.org/10.1097/YCT.0000000000000756

Dlabac-de Lange, J. J., Bais, L., van Es, F. D., Visser, B. G. J., Reinink, E., Bakker, B., van den Heuvel, E. R., Aleman, A., & Knegtering, H. (2015). Efficacy of bilateral repetitive transcranial magnetic stimulation for negative symptoms of schizophrenia: results of a multicenter double-blind randomized controlled trial. *Psychological Medicine*, *45*(6), 1263–1275. https://doi.org/10.1017/S0033291714002360

Ekhtiari, H., Soleimani, G., Kuplicki, R., Yeh, H.-W., Cha, Y.-H., & Paulus, M. (2022). Transcranial direct current stimulation to modulate fMRI drug cue reactivity in methamphetamine users: A randomized clinical trial. *Human Brain Mapping*, *43*(17), 5340–5357. https://ovidsp.ovid.com/ovidweb.cgi?T=JS&CSC=Y&NEWS=N&PAGE=fulltext&D=ovftz&AN=01445432-202210010-00020

Elbeh, K. A. M., Elserogy, Y. M. B., Khalifa, H. E., Ahmed, M. A., Hafez, M. H., & Khedr, E. M. (2016). Repetitive transcranial magnetic stimulation in the treatment of obsessive-compulsive disorders: Double blind randomized clinical trial. *Psychiatry Research*, *238*, 264–269. https://doi.org/10.1016/j.psychres.2016.02.031

Eschweiler, G. W., Wegerer, C., Schlotter, W., Spandl, C., Stevens, A., Bartels, M., & Buchkremer, G. (2000). Left prefrontal activation predicts therapeutic effects of repetitive transcranial magnetic stimulation (rTMS) in major depression. *Psychiatry Research*, *99*(3), 161–172. https://doi.org/10.1016/s0925-4927(00)00062-7

Fitzgerald, P. B., Brown, T. L., Marston, N. A. U., Daskalakis, Z. J., De Castella, A., & Kulkarni, J. (2003). Transcranial magnetic stimulation in the treatment of depression: a double-blind, placebo-controlled trial. *Archives of General Psychiatry*, *60*(10), 1002–1008. https://doi.org/10.1001/archpsyc.60.9.1002

Fitzgerald, P. B., Hoy, K. E., Herring, S. E., McQueen, S., Peachey, A. V. J., Segrave, R. A., Maller, J., Hall, P., & Daskalakis, Z. J. (2012). A double blind randomized trial of unilateral left and bilateral prefrontal cortex transcranial magnetic stimulation in treatment resistant major depression. *Journal of Affective Disorders*, *139*(2), 193–198. https://doi.org/10.1016/j.jad.2012.02.017

Fröhlich, F., Burrello, T. N., Mellin, J. M., Cordle, A. L., Lustenberger, C. M., Gilmore, J. H., & Jarskog, L. F. (2016). Exploratory study of once-daily transcranial direct current stimulation (tDCS) as a treatment for auditory hallucinations in schizophrenia. *European Psychiatry : The Journal of the Association of European Psychiatrists*, *33*, 54–60. https://doi.org/10.1016/j.eurpsy.2015.11.005

Gaudreault, P.-O., Sharma, A., Datta, A., Nakamura-Palacios, E. M., King, S., Malaker, P., Wagner, A., Vasa, D., Parvaz, M. A., Parra, L. C., Alia-Klein, N., & Goldstein, R. Z. (2021). A double-blind

sham-controlled phase 1 clinical trial of tDCS of the dorsolateral prefrontal cortex in cocaine inpatients: Craving, sleepiness, and contemplation to change. *The European Journal of Neuroscience*, *53*(9), 3212–3230. https://doi.org/10.1111/ejn.15172

George, M S, Nahas, Z., Molloy, M., Speer, A. M., Oliver, N. C., Li, X. B., Arana, G. W., Risch, S. C., & Ballenger, J. C. (2000). A controlled trial of daily left prefrontal cortex TMS for treating depression. *Biological Psychiatry*, *48*(10), 962–970. https://doi.org/10.1016/s0006-3223(00)01048-9

George, M S, Wassermann, E. M., Kimbrell, T. A., Little, J. T., Williams, W. E., Danielson, A. L., Greenberg, B. D., Hallett, M., & Post, R. M. (1997). Mood improvement following daily left prefrontal repetitive transcranial magnetic stimulation in patients with depression: a placebo-controlled crossover trial. *The American Journal of Psychiatry*, *154*(12), 1752–1756. https://doi.org/10.1176/ajp.154.12.1752

George, Mark S, Lisanby, S. H., Avery, D., McDonald, W. M., Durkalski, V., Pavlicova, M., Anderson, B., Nahas, Z., Bulow, P., Zarkowski, P., Holtzheimer, P. E. 3rd, Schwartz, T., & Sackeim, H. A. (2010). Daily left prefrontal transcranial magnetic stimulation therapy for major depressive disorder: a sham-controlled randomized trial. *Archives of General Psychiatry*, *67*(5), 507–516. https://doi.org/10.1001/archgenpsychiatry.2010.46

Gomes, J. S., Trevizol, A. P., Ducos, D. V, Gadelha, A., Ortiz, B. B., Fonseca, A. O., Akiba, H. T., Azevedo, C. C., Guimaraes, L. S. P., Shiozawa, P., Cordeiro, Q., Lacerda, A., & Dias, A. M. (2018). Effects of transcranial direct current stimulation on working memory and negative symptoms in schizophrenia: a phase II randomized sham-controlled trial. *Schizophrenia Research. Cognition*, *12*, 20–28. https://doi.org/10.1016/j.scog.2018.02.003

Gomes, P. V. O., Brasil-Neto, J. P., Allam, N., & Rodrigues de Souza, E. (2012). A randomized, double-blind trial of repetitive transcranial magnetic stimulation in obsessive-compulsive disorder with three-month follow-up. *The Journal of Neuropsychiatry and Clinical Neurosciences*, *24*(4), 437–443. https://doi.org/10.1176/appi.neuropsych.11100242

Gornerova, N., Brunovsky, M., Klirova, M., Novak, T., Zaytseva, Y., Koprivova, J., Bravermanova, A., & Horacek, J. (2023). The effect of low-frequency rTMS on auditory hallucinations, EEG source localization and functional connectivity in schizophrenia. *Neuroscience Letters*, *794*, 136977.

Guan, H. Y., Zhao, J. M., Wang, K. Q., Su, X. R., Pan, Y. F., Guo, J. M., Jiang, L., Wang, Y. H., Liu, H. Y., Sun, S. G., Wu, H. R., Ren, Y. P., Geng, H. S., Liu, X. W., Yu, H. J., Wei, B. C., Li, X. P., Wu, H. E., Tan, S. P., … Zhang, X. Y. (2020). High-frequency neuronavigated rTMS effect on clinical symptoms and cognitive dysfunction: a pilot double-blind, randomized controlled study in Veterans with schizophrenia. *Translational Psychiatry*, *10*(1), 79. https://doi.org/10.1038/s41398-020-0745-6

Haghighi, M., Shayganfard, M., Jahangard, L., Ahmadpanah, M., Bajoghli, H., Pirdehghan, A., Holsboer-Trachsler, E., & Brand, S. (2015). Repetitive Transcranial Magnetic Stimulation (rTMS) improves symptoms and reduces clinical illness in patients suffering from OCD--Results from a single-blind, randomized clinical trial with sham cross-over condition. *Journal of Psychiatric Research*, *68*, 238–244. https://doi.org/10.1016/j.jpsychires.2015.06.020

Holla, B., Biswal, J., Ramesh, V., Shivakumar, V., Bharath, R. D., Benegal, V., Venkatasubramanian, G., Chand, P. K., & Murthy, P. (2020). Effect of prefrontal tDCS on resting brain fMRI graph measures in Alcohol Use Disorders: A randomized, double-blind, sham-controlled study. *Progress in Neuro-Psychopharmacology & Biological Psychiatry*, *102*, 109950. https://doi.org/10.1016/j.pnpbp.2020.109950

Holtzheimer, P. E. 3rd, Russo, J., Claypoole, K. H., Roy-Byrne, P., & Avery, D. H. (2004). Shorter duration of depressive episode may predict response to repetitive transcranial magnetic stimulation. *Depression and Anxiety*, *19*(1), 24–30. https://doi.org/10.1002/da.10147

Höppner, J., Schulz, M., Irmisch, G., Mau, R., Schläfke, D., & Richter, J. (2003). Antidepressant efficacy of two different rTMS procedures. High frequency over left versus low frequency over right prefrontal cortex compared with sham stimulation. *European Archives of Psychiatry and Clinical Neuroscience, 253*(2), 103–109. https://doi.org/10.1007/s00406-003-0416-7

Hoy, K. E., Coyle, H., Gainsford, K., Hill, A., Bailey, N., & Fitzgerald, P. (2022). Investigating neurophysiological effects of a short course of tDCS for cognition in schizophrenia: a target engagement study. *MedRxiv*, 2022.03. 02.22271807.

Huang, W., Shen, F., Zhang, J., & Xing, B. (2016). Effect of Repetitive Transcranial Magnetic Stimulation on Cigarette Smoking in Patients with Schizophrenia. *Shanghai Archives of Psychiatry, 28*(6), 309–317. https://doi.org/10.11919/j.issn.1002-0829.216044

Jahangard, L., Haghighi, M., Shyayganfard, M., Ahmadpanah, M., Sadeghi Bahmani, D., Bajoghli, H., Holsboer-Trachsler, E., & Brand, S. (2016). Repetitive Transcranial Magnetic Stimulation Improved Symptoms of Obsessive-Compulsive Disorder, but Also Cognitive Performance: Results from a Randomized Clinical Trial with a Cross-Over Design and Sham Condition. *Neuropsychobiology, 73*(4), 224–232. https://doi.org/10.1159/000446287

Januel, D., Dumortier, G., Verdon, C.-M., Stamatiadis, L., Saba, G., Cabaret, W., Benadhira, R., Rocamora, J.-F., Braha, S., Kalalou, K., Vicaut, P. E., & Fermanian, J. (2006). A double-blind sham controlled study of right prefrontal repetitive transcranial magnetic stimulation (rTMS): therapeutic and cognitive effect in medication free unipolar depression during 4 weeks. *Progress in Neuro-Psychopharmacology & Biological Psychiatry, 30*(1), 126–130. https://doi.org/10.1016/j.pnpbp.2005.08.016

Jeon, D.-W., Jung, D.-U., Kim, S.-J., Shim, J.-C., Moon, J.-J., Seo, Y.-S., Jung, S.-S., Seo, B.-J., Kim, J.-E., Oh, M., & Kim, Y.-N. (2018). Adjunct transcranial direct current stimulation improves cognitive function in patients with schizophrenia: A double-blind 12-week study. *Schizophrenia Research, 197*, 378–385. https://doi.org/10.1016/j.schres.2017.12.009

Kang, J. I., Kim, C.-H., Namkoong, K., Lee, C.-I., & Kim, S. J. (2009). A randomized controlled study of sequentially applied repetitive transcranial magnetic stimulation in obsessive-compulsive disorder. *The Journal of Clinical Psychiatry, 70*(12), 1645–1651. https://doi.org/10.4088/JCP.08m04500

Kantrowitz, J. T., Sehatpour, P., Avissar, M., Horga, G., Gwak, A., Hoptman, M. J., Beggel, O., Girgis, R. R., Vail, B., Silipo, G., Carlson, M., & Javitt, D. C. (2019). Significant improvement in treatment resistant auditory verbal hallucinations after 5 days of double-blind, randomized, sham controlled, fronto-temporal, transcranial direct current stimulation (tDCS): A replication/extension study. *Brain Stimulation, 12*(4), 981–991. https://doi.org/10.1016/j.brs.2019.03.003

Kauffmann, C. D., Cheema, M. A., & Miller, B. E. (2004). Slow right prefrontal transcranial magnetic stimulation as a treatment for medication-resistant depression: a double-blind, placebo-controlled study. In *Depression and anxiety* (Vol. 19, Issue 1, pp. 59–62). https://doi.org/10.1002/da.10144

Khedr, E. M., Elbeh, K., Saber, M., Abdelrady, Z., & Abdelwarith, A. (2022). A double blind randomized clinical trial of the effectiveness of low frequency rTMS over right DLPFC or OFC for treatment of obsessive-compulsive disorder. *Journal of Psychiatric Research, 156*, 122–131.

Klauss, J., Anders, Q. S., Felippe, L. V, Nitsche, M. A., & Nakamura-Palacios, E. M. (2018). Multiple Sessions of Transcranial Direct Current Stimulation (tDCS) Reduced Craving and Relapses for Alcohol Use: A Randomized Placebo-Controlled Trial in Alcohol Use Disorder. *Frontiers in Pharmacology, 9,* 716. https://doi.org/10.3389/fphar.2018.00716

Klein, E., Kolsky, Y., Puyerovsky, M., Koren, D., Chistyakov, A., & Feinsod, M. (1999). Right prefrontal slow repetitive transcranial magnetic stimulation in schizophrenia: a double-blind sham-controlled

pilot study. *Biological Psychiatry*, *46*(10), 1451–1454. https://doi.org/10.1016/s0006-3223(99)00182-1

Koops, S., Blom, J. D., Bouachmir, O., Slot, M. I., Neggers, B., & Sommer, I. E. (2018). Treating auditory hallucinations with transcranial direct current stimulation in a double-blind, randomized trial. *Schizophrenia Research*, *201*, 329–336. https://doi.org/10.1016/j.schres.2018.06.010

Kreuzer, P. M., Schecklmann, M., Lehner, A., Wetter, T. C., Poeppl, T. B., Rupprecht, R., de Ridder, D., Landgrebe, M., & Langguth, B. (2015). The ACDC pilot trial: targeting the anterior cingulate by double cone coil rTMS for the treatment of depression. *Brain Stimulation*, *8*(2), 240–246. https://doi.org/10.1016/j.brs.2014.11.014

Kumar, N., Vishnubhatla, S., Wadhawan, A. N., Minhas, S., & Gupta, P. (2020). A randomized, double blind, sham-controlled trial of repetitive transcranial magnetic stimulation (rTMS) in the treatment of negative symptoms in schizophrenia. *Brain Stimulation*, *13*(3), 840–849. https://doi.org/10.1016/j.brs.2020.02.016

Lindenmayer, J. P., Kulsa, M. K. C., Sultana, T., Kaur, A., Yang, R., Ljuri, I., Parker, B., & Khan, A. (2019). Transcranial direct-current stimulation in ultra-treatment-resistant schizophrenia. *Brain Stimulation*, *12*(1), 54–61. https://doi.org/10.1016/j.brs.2018.10.002

Lisoni, J., Baldacci, G., Nibbio, G., Zucchetti, A., Butti Lemmi Gigli, E., Savorelli, A., Facchi, M., Miotto, P., Deste, G., Barlati, S., & Vita, A. (2022). Effects of bilateral, bipolar-nonbalanced, frontal transcranial Direct Current Stimulation (tDCS) on negative symptoms and neurocognition in a sample of patients living with schizophrenia: Results of a randomized double-blind sham-controlled trial. *J Psychiatr Res*, *155*, 430–442. https://doi.org/10.1016/j.jpsychires.2022.09.011

Loo, C. K., Alonzo, A., Martin, D., Mitchell, P. B., Galvez, V., & Sachdev, P. (2012). Transcranial direct current stimulation for depression: 3-week, randomised, sham-controlled trial. *The British Journal of Psychiatry : The Journal of Mental Science*, *200*(1), 52–59. https://doi.org/10.1192/bjp.bp.111.097634

Loo, C. K., Husain, M. M., McDonald, W. M., Aaronson, S., O'Reardon, J. P., Alonzo, A., Weickert, C. S., Martin, D. M., McClintock, S. M., Mohan, A., & Lisanby, S. H. (2018). International randomized-controlled trial of transcranial Direct Current Stimulation in depression. *Brain Stimulation*, *11*(1), 125–133. https://doi.org/10.1016/j.brs.2017.10.011

Loo, C. K., Sachdev, P., Martin, D., Pigot, M., Alonzo, A., Malhi, G. S., Lagopoulos, J., & Mitchell, P. (2010). A double-blind, sham-controlled trial of transcranial direct current stimulation for the treatment of depression. *The International Journal of Neuropsychopharmacology*, *13*(1), 61–69. https://doi.org/10.1017/S1461145709990411

Ma, X., Huang, Y., Liao, L., & Jin, Y. (2014). A randomized double-blinded sham-controlled trial of α electroencephalogram-guided transcranial magnetic stimulation for obsessive-compulsive disorder. *Chinese Medical Journal*, *127*(4), 601–606.

Mansur, C. G., Myczkowki, M. L., de Barros Cabral, S., Sartorelli, M. do C. B., Bellini, B. B., Dias, A. M., Bernik, M. A., & Marcolin, M. A. (2011). Placebo effect after prefrontal magnetic stimulation in the treatment of resistant obsessive-compulsive disorder: a randomized controlled trial. *The International Journal of Neuropsychopharmacology*, *14*(10), 1389–1397. https://doi.org/10.1017/S1461145711000575

Mantovani, A., Simpson, H. B., Fallon, B. A., Rossi, S., & Lisanby, S. H. (2010). Randomized sham-controlled trial of repetitive transcranial magnetic stimulation in treatment-resistant obsessive-compulsive disorder. *The International Journal of Neuropsychopharmacology*, *13*(2), 217–227. https://doi.org/10.1017/S1461145709990435

© 2024 Sabé M et al. *JAMA Network Open.*

Marquardt, L., Craven, A. R., Hugdahl, K., Johnsen, E., Kroken, R. A., Kusztrits, I., Specht, K., Thomassen, A. S., Weber, S., & Hirnstein, M. (2022). Pilot-RCT Finds No Evidence for Modulation of Neuronal Networks of Auditory Hallucinations by Transcranial Direct Current Stimulation. *Brain Sciences*, *12*(10), 1382.

Martinotti, G., Lupi, M., Montemitro, C., Miuli, A., Di Natale, C., Spano, M. C., Mancini, V., Lorusso, M., Stigliano, G., Tambelli, A., Di Carlo, F., Di Caprio, L., Fraticelli, S., Chillemi, E., Pettorruso, M., Sepede, G., & di Giannantonio, M. (2019). Transcranial Direct Current Stimulation Reduces Craving in Substance Use Disorders: A Double-blind, Placebo-Controlled Study. *The Journal of ECT*, *35*(3), 207–211. https://doi.org/10.1097/YCT.0000000000000580

McDonald, W. M., Easley, K., Byrd, E. H., Holtzheimer, P., Tuohy, S., Woodard, J. L., Beyer, K., & Epstein, C. M. (2006). Combination rapid transcranial magnetic stimulation in treatment refractory depression. *Neuropsychiatric Disease and Treatment*, *2*(1), 85–94.

Mogg, A., Pluck, G., Eranti, S. V, Landau, S., Purvis, R., Brown, R. G., Curtis, V., Howard, R., Philpot, M., & McLoughlin, D. M. (2008). A randomized controlled trial with 4-month follow-up of adjunctive repetitive transcranial magnetic stimulation of the left prefrontal cortex for depression. *Psychological Medicine*, *38*(3), 323–333. https://doi.org/10.1017/S0033291707001663

Moirand, R., Imbert, L., Haesebaert, F., Chesnoy, G., Bediou, B., Poulet, E., & Brunelin, J. (2022). Ten Sessions of 30 Min tDCS over 5 Days to Achieve Remission in Depression: A Randomized Pilot Study. *Journal of Clinical Medicine*, *11*(3), 782.

Nam, D.-H., Pae, C.-U., & Chae, J.-H. (2013). Low-frequency, Repetitive Transcranial Magnetic Stimulation for the Treatment of Patients with Posttraumatic Stress Disorder: a Double-blind, Sham-controlled Study. *Clinical Psychopharmacology and Neuroscience : The Official Scientific Journal of the Korean College of Neuropsychopharmacology*, *11*(2), 96–102. https://doi.org/10.9758/cpn.2013.11.2.96

Nauczyciel, C., Jeune, F., Naudet, F., Douabin, S., Esquevin, A., Vérin, M., Dondaine, T., Robert, G., Drapier, D., & Millet, B. (2014). Repetitive transcranial magnetic stimulation over the orbitofrontal cortex for obsessive-compulsive disorder: A double-blind, crossover study. *Translational Psychiatry*, *4*, e436. https://doi.org/10.1038/tp.2014.62

O'Reardon, J. P., Solvason, H. B., Janicak, P. G., Sampson, S., Isenberg, K. E., Nahas, Z., McDonald, W. M., Avery, D., Fitzgerald, P. B., Loo, C., Demitrack, M. A., George, M. S., & Sackeim, H. A. (2007). Efficacy and safety of transcranial magnetic stimulation in the acute treatment of major depression: a multisite randomized controlled trial. *Biological Psychiatry*, *62*(11), 1208–1216. https://doi.org/10.1016/j.biopsych.2007.01.018

Padberg, F, Zwanzger, P., Thoma, H., Kathmann, N., Haag, C., Greenberg, B. D., Hampel, H., & Möller, H. J. (1999). Repetitive transcranial magnetic stimulation (rTMS) in pharmacotherapy-refractory major depression: comparative study of fast, slow and sham rTMS. *Psychiatry Research*, *88*(3), 163–171. https://doi.org/10.1016/s0165-1781(99)00092-x

Padberg, Frank, Zwanzger, P., Keck, M. E., Kathmann, N., Mikhaiel, P., Ella, R., Rupprecht, P., Thoma, H., Hampel, H., Toschi, N., & Möller, H.-J. (2002). Repetitive transcranial magnetic stimulation (rTMS) in major depression: relation between efficacy and stimulation intensity. *Neuropsychopharmacology : Official Publication of the American College of Neuropsychopharmacology*, *27*(4), 638–645. https://doi.org/10.1016/S0893-133X(02)00338-X

Paillère-Martinot, M.-L., Galinowski, A., Plaze, M., Andoh, J., Bartrés-Faz, D., Bellivier, F., Lefaucheur, J.-P., Rivière, D., Gallarda, T., Martinot, J.-L., & Artiges, E. (2016). Active and placebo transcranial magnetic stimulation effects on external and internal auditory hallucinations of schizophrenia. *Acta Psychiatrica Scandinavica*, *135*. https://doi.org/10.1111/acps.12680

© 2024 Sabé M et al. *JAMA Network Open.*

Paillère Martinot, M.-L., Galinowski, A., Ringuenet, D., Gallarda, T., Lefaucheur, J.-P., Bellivier, F., Picq, C., Bruguière, P., Mangin, J.-F., Rivière, D., Willer, J.-C., Falissard, B., Leboyer, M., Olié, J.-P., Artiges, E., & Martinot, J.-L. (2010). Influence of prefrontal target region on the efficacy of repetitive transcranial magnetic stimulation in patients with medication-resistant depression: a [18F]-fluorodeoxyglucose PET and MRI study. *International Journal of Neuropsychopharmacology*, *13*(1), 45–59. https://doi.org/10.1017/S146114570900008X

Pelissolo, A., Harika-Germaneau, G., Rachid, F., Gaudeau-Bosma, C., Tanguy, M.-L., BenAdhira, R., Bouaziz, N., Popa, T., Wassouf, I., Saba, G., Januel, D., & Jaafari, N. (2016). Repetitive Transcranial Magnetic Stimulation to Supplementary Motor Area in Refractory Obsessive-Compulsive Disorder Treatment: a Sham-Controlled Trial. *The International Journal of Neuropsychopharmacology*, *19*(8). https://doi.org/10.1093/ijnp/pyw025

Prasko, J., Pasková, B., Záleský, R., Novák, T., Kopecek, M., Bares, M., & Horácek, J. (2006). The effect of repetitive transcranial magnetic stimulation (rTMS) on symptoms in obsessive compulsive disorder. A randomized, double blind, sham controlled study. *Neuro Endocrinology Letters*, *27*(3), 327–332.

Prasser, J., Schecklmann, M., Poeppl, T. B., Frank, E., Kreuzer, P. M., Hajak, G., Rupprecht, R., Landgrebe, M., & Langguth, B. (2015). Bilateral prefrontal rTMS and theta burst TMS as an add-on treatment for depression: a randomized placebo controlled trial. *The World Journal of Biological Psychiatry : The Official Journal of the World Federation of Societies of Biological Psychiatry*, *16*(1), 57–65. https://doi.org/10.3109/15622975.2014.964768

Prikryl, R., Kasparek, T., Skotakova, S., Ustohal, L., Kucerova, H., & Ceskova, E. (2007). Treatment of negative symptoms of schizophrenia using repetitive transcranial magnetic stimulation in a double-blind, randomized controlled study. *Schizophrenia Research*, *95*(1–3), 151–157. https://doi.org/10.1016/j.schres.2007.06.019

Prikryl, R., Mikl, M., Prikrylova Kucerová, H., Ustohal, L., Kasparek, T., Marecek, R., Vrzalova, M., Ceskova, E., & Vanicek, J. (2012). Does repetitive transcranial magnetic stimulation have a positive effect on working memory and neuronal activation in treatment of negative symptoms of schizophrenia? *Neuro Endocrinology Letters*, *33*(1), 90–97.

Prikryl, R., Ustohal, L., Kucerova, H. P., Kasparek, T., Jarkovsky, J., Hublova, V., Vrzalova, M., & Ceskova, E. (2014). Repetitive transcranial magnetic stimulation reduces cigarette consumption in schizophrenia patients. *Progress in Neuro-Psychopharmacology & Biological Psychiatry*, *49*, 30–35. https://doi.org/10.1016/j.pnpbp.2013.10.019

Quan, W. X., Zhu, X. L., Qiao, H., Zhang, W. F., Tan, S. P., Zhou, D. F., & Wang, X. Q. (2015). The effects of high-frequency repetitive transcranial magnetic stimulation (rTMS) on negative symptoms of schizophrenia and the follow-up study. *Neuroscience Letters*, *584*, 197–201. https://doi.org/10.1016/j.neulet.2014.10.029

Ruffini, C., Locatelli, M., Lucca, A., Benedetti, F., Insacco, C., & Smeraldi, E. (2009). Augmentation Effect of Repetitive Transcranial Magnetic Stimulation Over the Orbitofrontal Cortex in Drug-Resistant Obsessive-Compulsive Disorder Patients. *Primary Care Companion to the Journal of Clinical Psychiatry*, *11*, 226–230. https://doi.org/10.4088/PCC.08m00663

Sachdev, P. S., Loo, C. K., Mitchell, P. B., McFarquhar, T. F., & Malhi, G. S. (2007). Repetitive transcranial magnetic stimulation for the treatment of obsessive compulsive disorder: a double-blind controlled investigation. *Psychological Medicine*, *37*(11), 1645–1649. https://doi.org/10.1017/S0033291707001092

Salehinejad, M. A. (2015). Transcranial Direct Current Stimulation of Dorsolateral Prefrontal Cortex of Major Depression: Improving Visual Working Memory, Reducing Depressive Symptoms.

© 2024 Sabé M et al. *JAMA Network Open.*

Neuroregulation, 2(1). https://doi.org/10.15540/nr.2.1.37

Salehinejad, M. A., Ghanavai, E., Rostami, R., & Nejati, V. (2017). Cognitive control dysfunction in emotion dysregulation and psychopathology of major depression (MD): Evidence from transcranial brain stimulation of the dorsolateral prefrontal cortex (DLPFC). *Journal of Affective Disorders*, *210*, 241–248. https://doi.org/10.1016/j.jad.2016.12.036

Sampaio-Junior, B., Tortella, G., Borrione, L., Moffa, A. H., Machado-Vieira, R., Cretaz, E., Fernandes da Silva, A., Fraguas, R., Aparício, L. V, Klein, I., Lafer, B., Goerigk, S., Benseñor, I. M., Lotufo, P. A., Gattaz, W. F., & Brunoni, A. R. (2018). Efficacy and Safety of Transcranial Direct Current Stimulation as an Add-on Treatment for Bipolar Depression: A Randomized Clinical Trial. *JAMA Psychiatry*, *75*(2), 158–166. https://doi.org/10.1001/jamapsychiatry.2017.4040

Shayganfard, M., Jahangard, L., Nazaribadie, M., Haghighi, M., Ahmadpanah, M., Sadeghi Bahmani, D., Bajoghli, H., Holsboer-Trachsler, E., & Brand, S. (2016). Repetitive Transcranial Magnetic Stimulation Improved Symptoms of Obsessive-Compulsive Disorders but Not Executive Functions: Results from a Randomized Clinical Trial with Crossover Design and Sham Condition. *Neuropsychobiology*, *74*(2), 115–124. https://doi.org/10.1159/000457128

Singh, S., Kumar, N., Verma, R., & Nehra, A. (2020). The safety and efficacy of adjunctive 20-Hz repetitive transcranial magnetic stimulation for treatment of negative symptoms in patients with schizophrenia: A double-blinded, randomized, sham-controlled study. *Indian Journal of Psychiatry*, *62*(1), 21–29. https://doi.org/10.4103/psychiatry.IndianJPsychiatry_361_19

Soleimani, G., Towhidkhah, F., Oghabian, M. A., & Ekhtiari, H. (2022). DLPFC stimulation alters large-scale brain networks connectivity during a drug cue reactivity task: A tDCS-fMRI study. *Frontiers in Systems Neuroscience*, *16*, 17. https://doi.org/10.3389/fnsys.2022.956315

Speer, A. M., Wassermann, E. M., Benson, B. E., Herscovitch, P., & Post, R. M. (2014). Antidepressant efficacy of high and low frequency rTMS at 110% of motor threshold versus sham stimulation over left prefrontal cortex. *Brain Stimulation*, *7*(1), 36–41. https://doi.org/10.1016/j.brs.2013.07.004

Su TP, Huang CC, Wei IH. Add-on rTMS for medication-resistant depression: a randomized, double-blind, sham-controlled trial in Chinese patients. J Clin Psychiatry. 2005 Jul;66(7):930-7. doi: 10.4088/jcp.v66n0718Su, H., Liu, Y., Yin, D., Chen, T., Li, X., Zhong, N., Jiang, H., Wang, J., Du, J., Xiao, K., Xu, D., Zeljic, K., Wang, Z., & Zhao, M. (2020). Neuroplastic changes in resting-state functional connectivity after rTMS intervention for methamphetamine craving. *Neuropharmacology*, *175*, 108177. https://doi.org/https://doi.org/10.1016/j.neuropharm.2020.108177

Su, H., Zhong, N., Gan, H., Wang, J., Han, H., Chen, T., Li, X., Ruan, X., Zhu, Y., Jiang, H., & Zhao, M. (2017). High frequency repetitive transcranial magnetic stimulation of the left dorsolateral prefrontal cortex for methamphetamine use disorders: A randomised clinical trial. *Drug and Alcohol Dependence*, *175*, 84–91. https://doi.org/10.1016/j.drugalcdep.2017.01.037

Taylor, S. F., Ho, S. S., Abagis, T., Angstadt, M., Maixner, D. F., Welsh, R. C., & Hernandez-Garcia, L. (2018). Changes in brain connectivity during a sham-controlled, transcranial magnetic stimulation trial for depression. *Journal of Affective Disorders*, *232*, 143–151. https://doi.org/10.1016/j.jad.2018.02.019

Tong, J., Zhang, J., Jin, Y., Liu, W., Wang, H., Huang, Y., Shi, D., Zhu, M., Zhu, N., Zhang, T., & Sun, X. (2021). Impact of Repetitive Transcranial Magnetic Stimulation (rTMS) on Theory of Mind and Executive Function in Major Depressive Disorder and Its Correlation with Brain-Derived Neurotrophic Factor (BDNF): A Randomized, Double-Blind, Sham-Controlled Trial. *Brain Sciences*, *11*(6). https://doi.org/10.3390/brainsci11060765

Triggs, W. J., Ricciuti, N., Ward, H. E., Cheng, J., Bowers, D., Goodman, W. K., Kluger, B. M., &

© 2024 Sabé M et al. *JAMA Network Open.*

Nadeau, S. E. (2010). Right and left dorsolateral pre-frontal rTMS treatment of refractory depression: a randomized, sham-controlled trial. *Psychiatry Research*, *178*(3), 467–474. https://doi.org/10.1016/j.psychres.2010.05.009

Tsai, Y., Li, C., Liang, W., Muggleton, N. G., Tsai, C., Huang, N. E., & Juan, C. (2022). Critical role of rhythms in prefrontal transcranial magnetic stimulation for depression: A randomized sham-controlled study. *Human Brain Mapping*, *43*(5), 1535–1547.

van Eijndhoven, P. F. P., Bartholomeus, J., Möbius, M., de Bruijn, A., Ferrari, G. R. A., Mulders, P., Schene, A. H., Schutter, D. J. L. G., Spijker, J., & Tendolkar, I. (2020). A randomized controlled trial of a standard 4-week protocol of repetitive transcranial magnetic stimulation in severe treatment resistant depression. *Journal of Affective Disorders*, *274*, 444–449. https://doi.org/10.1016/j.jad.2020.05.055

Watts, B. V, Landon, B., Groft, A., & Young-Xu, Y. (2012). A sham controlled study of repetitive transcranial magnetic stimulation for posttraumatic stress disorder. *Brain Stimulation*, *5*(1), 38–43. https://doi.org/10.1016/j.brs.2011.02.002

Wen, N., Chen, L., Miao, X., Zhang, M., Zhang, Y., Liu, J., Xu, Y., Tong, S., Tang, W., Wang, M., Liu, J., Zhou, S., Fang, X., & Zhao, K. (2021). Effects of High-Frequency rTMS on Negative Symptoms and Cognitive Function in Hospitalized Patients With Chronic Schizophrenia: A Double-Blind, Sham-Controlled Pilot Trial . In *Frontiers in Psychiatry* (Vol. 12). https://www.frontiersin.org/articles/10.3389/fpsyt.2021.736094

Wobrock, T., Guse, B., Cordes, J., Wölwer, W., Winterer, G., Gaebel, W., Langguth, B., Landgrebe, M., Eichhammer, P., Frank, E., Hajak, G., Ohmann, C., Verde, P. E., Rietschel, M., Ahmed, R., Honer, W. G., Malchow, B., Schneider-Axmann, T., Falkai, P., & Hasan, A. (2015). Left prefrontal high-frequency repetitive transcranial magnetic stimulation for the treatment of schizophrenia with predominant negative symptoms: a sham-controlled, randomized multicenter trial. *Biological Psychiatry*, *77*(11), 979–988. https://doi.org/10.1016/j.biopsych.2014.10.009

Xie, Y., Guan, M., He, Y., Wang, Z., Ma, Z., Fang, P., & Wang, H. (2023). The Static and dynamic functional connectivity characteristics of the left temporoparietal junction region in schizophrenia patients with auditory verbal hallucinations during low-frequency rTMS treatment. *Frontiers in Psychiatry*, *14*.

Xiu, M. H., Guan, H. Y., Zhao, J. M., Wang, K. Q., Pan, Y. F., Su, X. R., Wang, Y. H., Guo, J. M., Jiang, L., Liu, H. Y., Sun, S. G., Wu, H. R., Geng, H. S., Liu, X. W., Yu, H. J., Wei, B. C., Li, X. P., Trinh, T., Tan, S. P., & Zhang, X. Y. (2020). Cognitive Enhancing Effect of High-Frequency Neuronavigated rTMS in Chronic Schizophrenia Patients With Predominant Negative Symptoms: A Double-Blind Controlled 32-Week Follow-up Study. *Schizophrenia Bulletin*, *46*(5), 1219–1230. https://doi.org/10.1093/schbul/sbaa035

Zhao, S., Kong, J., Li, S., Tong, Z., Yang, C., & Zhong, H. (2014). Randomized controlled trial of four protocols of repetitive transcranial magnetic stimulation for treating the negative symptoms of schizophrenia. *Shanghai Archives of Psychiatry*, *26*(1), 15–21. https://doi.org/10.3969/j.issn.1002-0829.2014.01.003

Zheng, H., Zhang, L., Li, L., Liu, P., Gao, J., Liu, X., Zou, J., Zhang, Y., Liu, J., Zhang, Z., Li, Z., & Men, W. (2010). High-frequency rTMS treatment increases left prefrontal myo-inositol in young patients with treatment-resistant depression. *Progress in Neuro-Psychopharmacology & Biological Psychiatry*, *34*(7), 1189–1195. https://doi.org/10.1016/j.pnpbp.2010.06.009

Zheng, L., Guo, Q., Li, H., Li, C., & Wang, J.-J. (2012). [Effects of repetitive transcranial magnetic stimulation with different paradigms on the cognitive function and psychotic symptoms of schizophrenia patients]. *Beijing da xue xue bao. Yi xue ban = Journal of Peking University. Health*

sciences, *44*(5), 732–736.

Zhuo, K., Tang, Y., Song, Z., Wang, Y., Wang, J., Qian, Z., Li, H., Xiang, Q., Chen, T., Yang, Z., Xu, Y., Fan, X., Wang, J., & Liu, D. (2019). Repetitive transcranial magnetic stimulation as an adjunctive treatment for negative symptoms and cognitive impairment in patients with schizophrenia: a randomized, double-blind, sham-controlled trial. *Neuropsychiatric Disease and Treatment*, *15*, 1141–1150. https://doi.org/10.2147/NDT.S196086

© 2024 Sabé M et al. *JAMA Network Open.*
