# Supplementary material for: Transcranial Magnetic Stimulation and Transcranial Direct Current Stimulation Across Mental Disorders: A Systematic Review and Dose-Response Meta-Analysis
Source: JAMA Netw Open. 2024 May 22;7(5):e2412616. doi: 10.1001/jamanetworkopen.2024.12616 (PMC11112448; doi:10.1001/jamanetworkopen.2024.12616)
Supplement: Supplement 2. — Data Sharing Statement [file jamanetwopen-e2412616-s002.pdf]

Data Sharing Statement

Sabé. Transcranial Magnetic Stimulation and Transcranial Direct Current Stimulation Across Mental Disorders. *JAMA Netw Open*. Published May 22, 2024.
doi:10.1001/jamanetworkopen.2024.12616

Data
Data available: No

Additional Information
Explanation for why data not available: Data of this meta-analysis is available after contacting the corresponding author: michel.sabe@hcuge.ch
All materials, including data, and the analysis have been made publicly available (https://osf.io/8b4yn/?view_only=6d7a45aeae70481da7c7c4a4771b1b04).
